# 1,2,3-Triazoles as leaving groups in S_N_Ar–Arbuzov reactions: synthesis of C6-phosphonated purine derivatives

**DOI:** 10.3762/bjoc.17.19

**Published:** 2021-01-20

**Authors:** Kārlis-Ēriks Kriķis, Irina Novosjolova, Anatoly Mishnev, Māris Turks

**Affiliations:** 1 Faculty of Materials Science and Applied Chemistry, Riga Technical University, P. Valdena Str. 3, LV-1048 Riga, Latvia; 2 Latvian Institute of Organic Synthesis, Aizkraukles Str. 21, LV-1006, Riga, Latvia

**Keywords:** Arbuzov reaction, 2,6-bistriazolylpurines, nucleophilic aromatic substitution, purinylphosphonates

## Abstract

A new method for C–N bond transformations into C–P bonds was developed using 1,2,3-triazoles as leaving groups in S_N_Ar–Arbuzov reactions. A series of C6-phosphonated 2-triazolylpurine derivatives was synthesized for the first time, with the isolated yields reaching up to 82% in the C–P-bond-forming event. The S_N_Ar–Arbuzov reaction of 2,6-bistriazolylpurines follows the general regioselectivity pattern of the C6-position being more reactive towards substitution, which was unambiguously proved by X-ray analysis of diethyl (9-heptyl-2-(4-phenyl-1*H*-1,2,3-triazol-1-yl)-9*H*-purin-6-yl)phosphonate.

## Introduction

Acyclic nucleoside phosphonates (ANPs) are an important compound class due to their biological activity profile [1–6]. Compounds bearing a phosphonate moiety in their N9 side chain are well known as antiviral agents, such as adefovir, tenofovir, and cidofovir [7]. Lately, it was found that ANPs possess inhibitory activity against hypoxanthine-guanine-xanthine phosphoribosyltransferase of the parasite *Plasmodium falciparum*, and several research groups are focused on the development of this topic [8–11]*.*

**Scheme 1:**
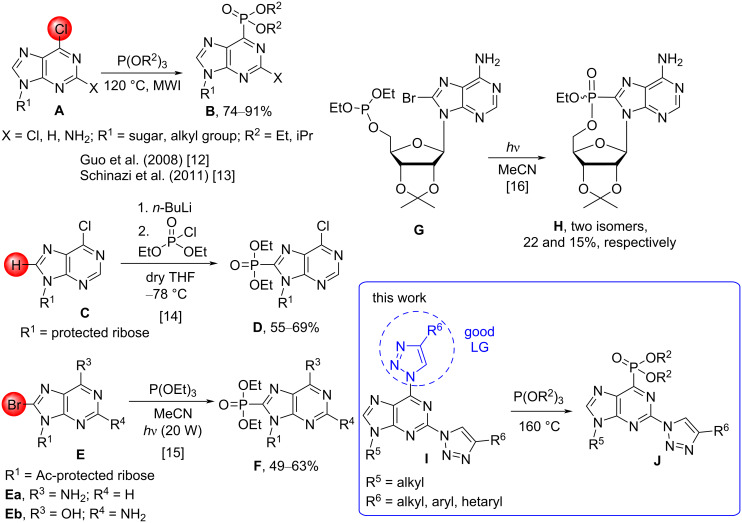
Structural diversity and synthetic methods of purinylphosphonates. MWI = microwave irradiation; LG = leaving group.

On the contrary, only a few examples can be found in the literature where a phosphorus-containing substituent is directly attached to the purine ring [12–13]. In 2008, an S_N_Ar–Arbuzov reaction was developed for 6-chloropurine derivatives under microwave irradiation (Scheme 1) [12]. In 2011, a single example of a C6-phosphonate, **B** (X = NH_2_; R^1^ = 2’-*C*-methylribose; R^2^ = Et), was synthesized among other compounds as a potential anti-hepatitis C virus agent and showed 19% inhibition at 10 μM in Huh7 cells (Scheme 1) [13]. Additionally, there are a few examples of C8-phosphonate synthesis. They can be obtained by 1) the reaction of a lithiated C8 position with diethyl chlorophosphate (**C**→**D**, Scheme 1) [14] and 2) an intermolecular [15] or intramolecular [16] photochemical reaction between 8-bromopurine derivatives and phosphite (**E**→**F** and **G→H**, respectively, Scheme 1). Further, the synthesis of C8-phosphonates of 7- and 9-deazapurines via C–H phosphonation has been reported [17].

On the other hand, azolylpurines are an important compound class that combines two recognized structural motifs of drug design – purines and azoles. Derivatives of this class are known for their activity against *Mycobacterium tuberculosis* and also as agonists and antagonists of adenosine receptors [18].

In 2013, we developed an efficient approach for the synthesis of ribo- and arabino-2,6-bistriazolylpurine nucleosides and showed that the triazolyl ring in the C6 position of purine acts as a good leaving group in S_N_Ar reactions with S- and N-nucleophiles [19–21]. It is worth to note that 2/6-amino-6/2-triazolylpurines possess high levels of fluorescence [19,22–24].

Herein, we describe an extension for S_N_Ar reactions that makes use of the 1,2,3-triazole leaving group of 2,6-bistriazolylpurines. This led to a discovery of novel C–P bond formations from C–N bonds in S_N_Ar–Arbuzov reactions (**I**→**J**, Scheme 1). The obtained series of compounds combines three structural motifs that are important in terms of medicinal chemistry in one molecule: purine, triazole, and phosphonate.

## Results and Discussion

### Synthetic approaches towards C6-phosphonated 2-triazolylpurines

**Scheme 2:**
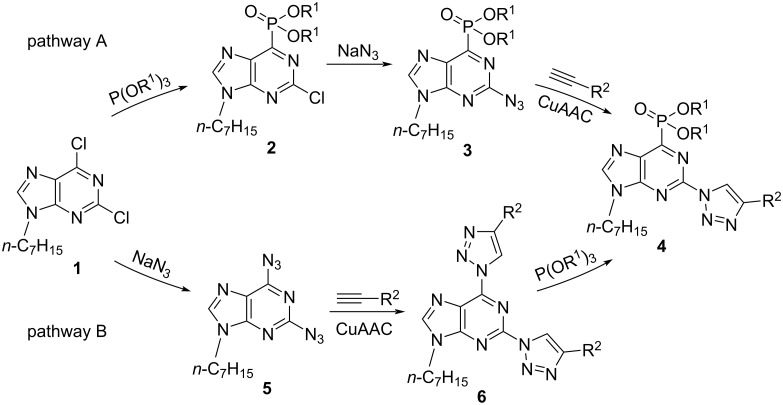
Synthetic routes for the formation of purinylphosphonates **4**.

Aiming to synthesize C6-phosphonated 2-triazolylpurines, we designed two synthetic routes (Scheme 2). Pathway A included: 1) a known S_N_Ar–Arbuzov reaction between 2,6-dichloropurine derivative **1** and P(OEt)_3_ [12], 2) substitution of chlorine at the purine C2 position by azide, and 3) copper-catalyzed azide–alkyne 1,3-dipolar cycloaddition (CuAAC) with different alkynes. Pathway B included: 1) the two-step synthesis of 2,6-bistriazolylpurine derivatives **6** from 2,6-dichloropurine derivative **1** [22] and 2) the S_N_Ar–Arbuzov reaction with phosphite.

**Scheme 3:**
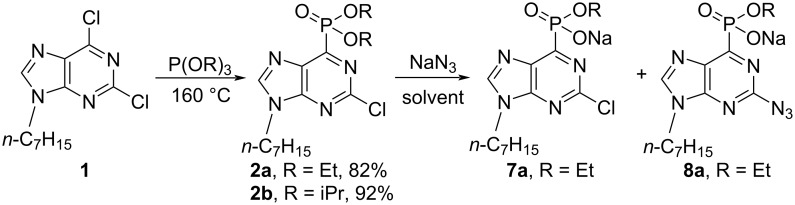
Synthesis of phosphonates **2**, **7**, and **8**.

The S_N_Ar–Arbuzov reaction between 2,6-dichloropurine derivative **1** and triethylphosphite gave product **2a** in 82% yield (Scheme 3) [12]. Next, attempts to substitute the chlorine atom at the purine C2 position were made using either NaN_3_ or TBAN_3_. Azidation experiments were tried in solvents such as EtOH, MeOH, and MeCN in temperature diapasons up to 100 °C, but no conversion of the staring material **2a** (R^1^ = Et) was observed. The change of the solvent to DMF or DMSO resulted in the cleavage of one ethyl ester group [25], but still the S_N_Ar reaction at C2 was not effective. LC–MS analysis of the crude reaction mixtures revealed the presence of the products **7a** and **8a** (Scheme 3). When the latter mixture was submitted to CuAAC with phenylacetylene (CuI/Et_3_N/AcOH/EtOH (or DCM), CuSO_4_∙5H_2_O/sodium ascorbate/EtOH (or DMF)), no triazole formation at the purine C2 position was observed.

**Scheme 4:**
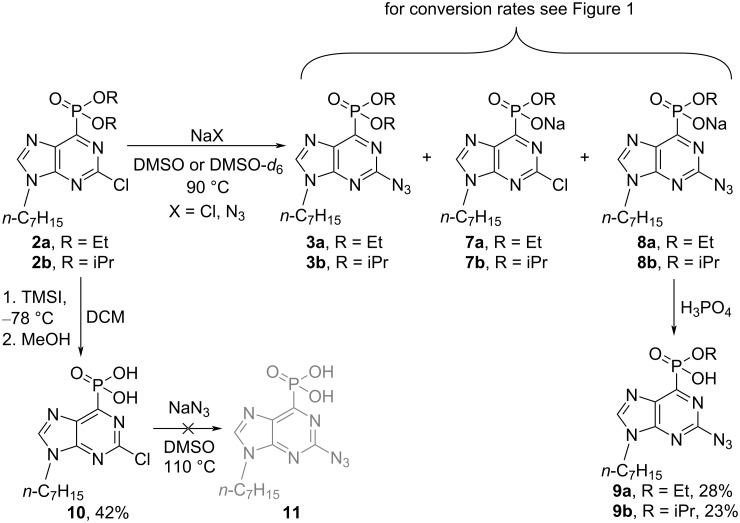
Synthesis of phosphonic acid monoesters **3** and **7**–**9** as well as phosphonic acid **10**.

**Figure 1:**
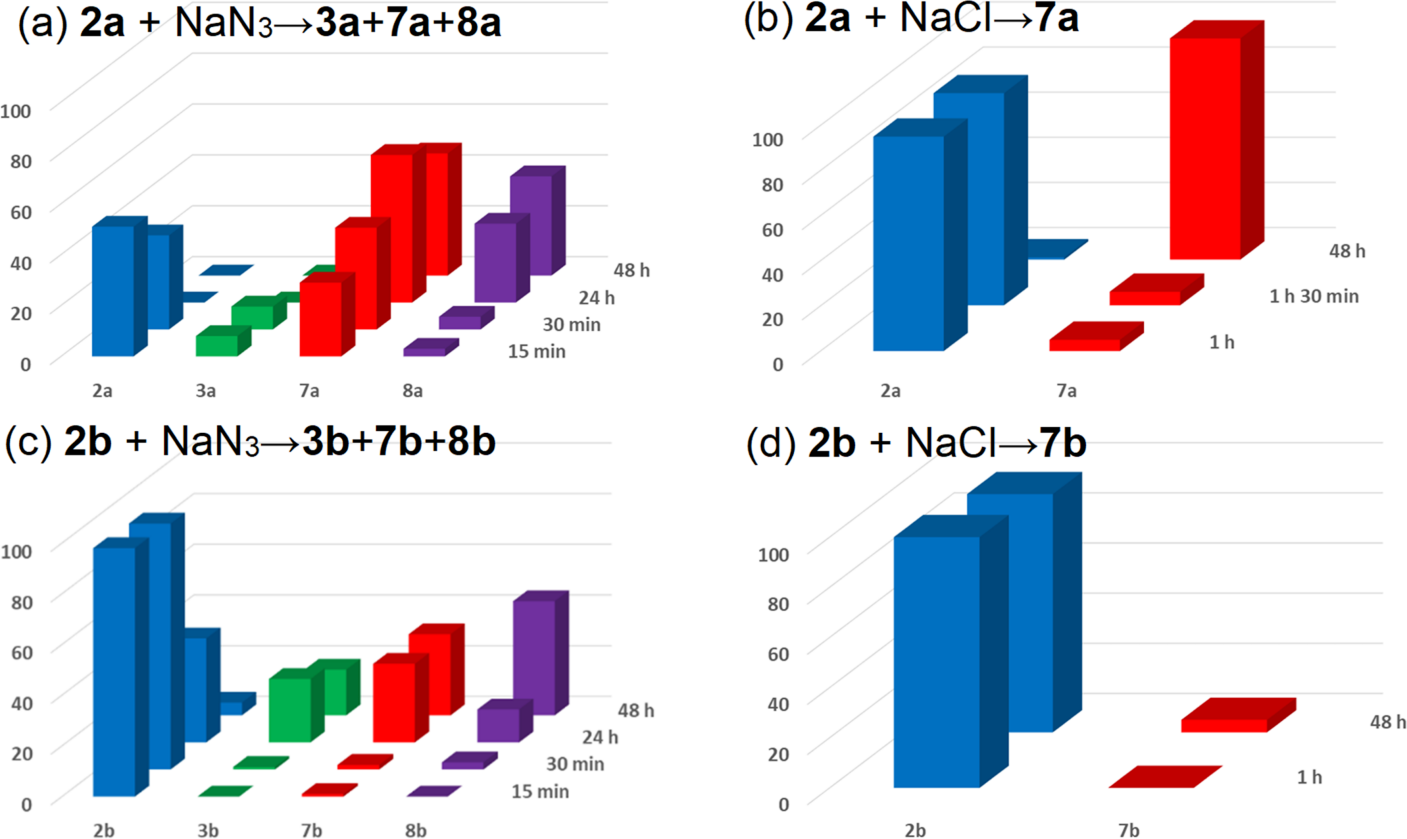
Screenings of the rate for the ester group cleavage (conversion determined by NMR spectroscopy) in the reactions between dialkyl (2-chloro-9-heptyl-9*H*-purin-6-yl)phosphonates **2a** and **2b**, respectively, with NaN_3_ (a, c) and NaCl (b, d). Reaction conditions: DMSO-*d*_6_, 90 °C.

We briefly tried to optimize the Cl→N_3_ S_N_Ar process at the purine C2 position, and that way, the isopropyl phosphonate **2b** was also obtained. It is known that both chloride and azide can cleave phosphonate esters [25–28], but the chloride source would not interfere with the S_N_Ar process at C2. Hence, we compared the reaction outcome and rates when DMSO-*d*_6_ solutions of the starting materials **2a** and **2b** were treated either with NaN_3_ or NaCl in parallel experiments. The reaction mixtures were directly analyzed by ^1^H and ^31^P NMR spectroscopy using 1,2,3-trimethoxybenzene as an internal standard (Tables S1 and S2 as well as Figures S1 and S2 in Supporting Information File 1). The reaction between the diethyl phosphonate **2a** and NaN_3_ gave a mixture of products **3a**, **7a**, and **8a** already after 15 min. A significant amount of the azido monoester **8a** (39%) was formed in only 48 h (Scheme 4, Figure 1, and Table S1 in Supporting Information File 1). The cleavage of the ester groups in the presence of NaCl was slower than in the presence of NaN_3_ (Figure 1 and Table S2 in Supporting Information File 1). Further, the cleavage of the sterically bulky isopropyl ester from phosphonate **2b** showed a similar pattern: 5% conversation to monoester **7b** was observed with NaCl after 48 h (Scheme 4, Figure 1, and Table S2 in Supporting Information File 1), but the reaction with NaN_3_ resulted in a mixture of products, which contained 45% of 2-azido monoester **8b** (Scheme 4).

Based on the previous observations, we forced the S_N_Ar reaction of the Cl atom at the C2 position of purine with an excess of NaN_3_, and after chromatographic isolation. We obtained the pure azido-substituted phosphonate monoesters **9a** and **9b** in 28 and 23% yield, respectively (Scheme 4). The products **9a** and **9b** were further submitted to CuAAC reactions, but the desired triazole derivatives were not obtained. Furthermore, the hydrolysis of the dialkyl ester groups were performed with TMSI [29–30], and phosphonic acid **10** was obtained. The latter was inert to the S_N_Ar reaction with NaN_3_ at C2 (Scheme 4).

### S_N_Ar–Arbuzov reaction between 2,6-bistriazolylpurines and P(OEt)_3_

**Scheme 5:**
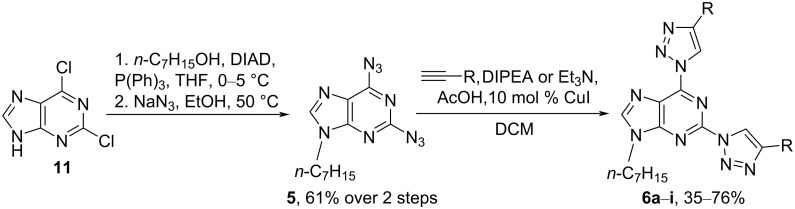
Synthesis of 2,6-bistriazolylpurine derivatives **6a**–**i**.

Next, we switched to pathway B (Scheme 2) and prepared 2,6-diazidopurine derivative **5** from 2,6-dichloropurine (**11**) via a Mitsunobu alkylation and S_N_Ar reaction with NaN_3_ (Scheme 5) [22]. 2,6-Bistriazolylpurine derivatives **6a**–**i** were obtained in CuAAC reactions with various alkynes in 35–76% yield (Table 1). We found that a combination of CuI with an amine buffer system [31–37] suites substrate **5** better than the previously used CuSO_4_∙5H_2_O and sodium ascorbate catalytic system [22]. Most probably, this is due to the solubility issues of the starting material **5** in aqueous solutions, as used in the Cu(II) and ascorbate protocol. In some cases, the use of Et_3_N lowered the yield of 2,6-bistriazolylpurines **6c** and **6f**–**i** due to the competing Glaser coupling [38–39] and the reduction of 2,6-diazide **5** by the Cu(I) species [40–41]. The bistriazolyl derivatives **6a**–**i** were easily crystalized from MeOH, EtOH, or a hexane/EtOH mixture or purified by column chromatography.

**Table 1:** Synthesis of 2,6-bistriazolylpurines **6a**–**i** (**5**→**6a**–**i**) according to Scheme 5.

entry	R	additive	*t*, h	yield of **6**, %

1	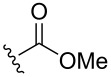	DIPEA	2	**6a**, 76
2	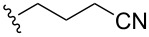	DIPEA	9	**6b**, 73
3	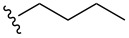	Et_3_N	3.5	**6c**, 57
4	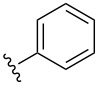	Et_3_N	3	**6d**, 70
5	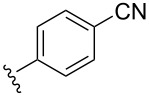	Et_3_N	12	**6e**, 70
6	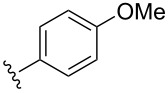	Et_3_N	1.5	**6f**, 46
7	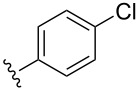	Et_3_N	15	**6g**, 35
8	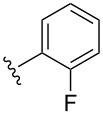	Et_3_N	3	**6h**, 58
9	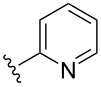	Et_3_N	9	**6i**, 35

**Scheme 6:**
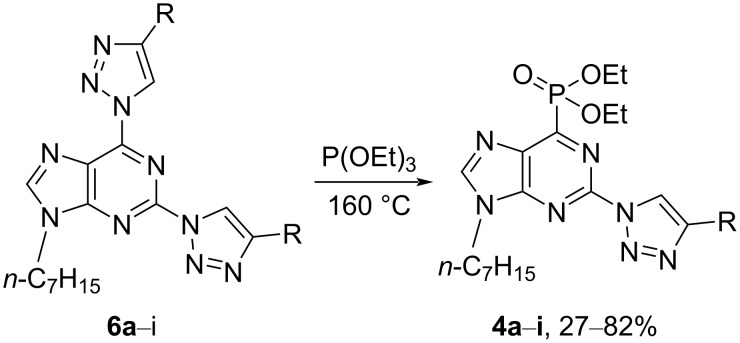
S_N_Ar–Arbuzov reaction between the bistriazolylpurines **6a**–**i** and P(OEt)_3_.

The obtained 2,6-bistriazolylpurine derivatives **6a**–**i** were explored as substrates for the S_N_Ar–Arbuzov reaction with P(OEt)_3_. In attempts to perform the S_N_Ar–Arbuzov reaction in common laboratory solvents, such as toluene, MeCN, and DCM, and in the presence of 1–20 equiv of P(OEt)_3_, the formation of the desired phosphonates **4** was not observed (Scheme 6). We started an optimization of the reaction conditions using substrate **6d**, and reactions in neat phosphite at various temperatures were tried (Table 2). The conversion of starting material **6d** was monitored by HPLC, and after completion, product **4d** was precipitated from the reaction mixture by hexane. For entries 1 and 3 in Table 2, an extra purification step by silica gel column chromatography was required. For compound **4d**, the optimal reaction conditions were 2 hours in neat P(OEt)_3_ at 160 °C.

**Table 2:** Optimization of the S_N_Ar–Arbuzov reaction conditions for **6d→4d** according to Scheme 6.

entry	*T*, °C	*t*, h	conversion of **6d**, %^a^	yield, %^b^

1	140	1	9	30
		2	19	
		3	49	
		4	75	
		5	85	
		6	91	

2	150	1	63	50
		2	81	
		3	91	

3	160	1	85	67
		2	96	

4	170	1	92	50
		2	96	

^a^The conversion was determined by HPLC analysis (column: XBridge C18, 4.6 × 150 mm, particle size 3.5 μm, flow rate 1 mL/min. Gradient: 30–95% B 5 min, 95% B 5 min, 95–30% B 2 min. Eluent A: 0.1% TFA in water with 5 vol % MeCN; eluent B: MeCN). ^b^Isolated yield after purification.

With the experimental conditions in hand, the S_N_Ar–Arbuzov reaction between 2,6-bistriazolylpurines **6a**–**i** and P(OEt)_3_ provided a library of novel purine phosphonates **4a**–**i** in 27–82% yield (Table 3). The products **4a**, **4d**, **4e**, and **4i** were easily precipitated from hexane left at −20 °C within 10 hours and were then filtered and washed with cold hexane. The product purity, if necessary, was further improved by column chromatography. Some phosphonates, for example, **4b**, **4c**, and **4f**, were reductant to precipitate from hexane and were purified solely by silica gel column chromatography. At the preparative level, the excess of P(OEt)_3_ was evaporated under vacuum (5 mbar) over 4–5 hours at 50 °C before further purification.

**Table 3:** S_N_Ar–Arbuzov reactions between 2,6-bistriazolylpurines **6a**–**i** and P(OEt)_3_ according to Scheme 6.

entry	R	*t*, h	yield of **4**, %

1	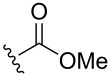	3	**4a**, 72
2	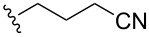	20	**4b**, 44
3	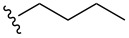	14	**4c**, 30
4	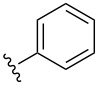	6	**4d**, 76
5	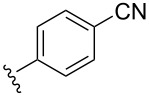	2	**4e**, 82
6	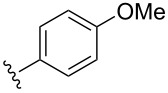	23	**4f**, 40
7	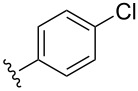	9	**4g**, 80
8	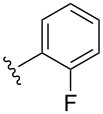	8	**4h**, 27
9	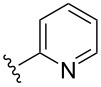	14	**4i**, 70

**Figure 2:**
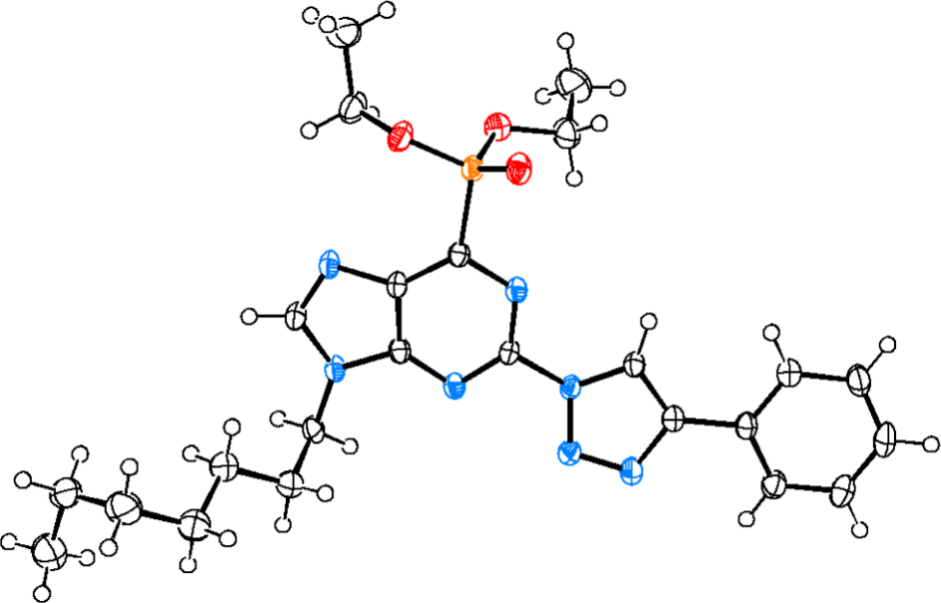
Single-crystal X-ray analysis of diethyl (9-heptyl-2-(4-phenyl-1*H*-1,2,3-triazol-1-yl)-9*H*-purin-6-yl)phosphonate (**4d**). CCDC-2044976.

The regioselectivity of the newly developed S_N_Ar–Arbuzov reaction was unambiguously established by X-ray analysis of the product **4d**, which was crystalized from a mixture of hexane and DCM using the slow-evaporation technique (Figure 2). This follows the previously reported regioselective C6-substitution of 2,6-bistriazolylpurines in S_N_Ar transformations.

## Conclusion

We have developed a novel S_N_Ar–Arbuzov transformation that makes use of 1,2,3-triazole as a leaving group. This has permitted to obtain a novel series of C6-phosphonated 2-triazolylpurine derivatives. It was also demonstrated that there is no alternative S_N_Ar protocol towards the designed products. The synthetic intermediates, (2-chloro-9*H*-purin-6-yl)phosphonates, of the alternative pathway are sluggish in substitution reactions with NaN_3_, and the burdensomely obtained (2-azido-9*H*-purin-6-yl)phosphonates fail to undergo CuAAC reactions. The developed S_N_Ar–Arbuzov reaction between 2,6-bistriazolylpurine derivatives and trialkyl phosphites is C6-regioselective, as proved by single-crystal X-ray analysis. This is similar to the previously observed substitution pattern in S_N_Ar reactions of 2,6-bistriazolylpurine derivatives with simple N- and S-nucleophiles.

## Experimental

### General information

Commercially available reagents were used as received. The reactions and the purity of the synthesized compounds were monitored by HPLC and TLC analysis using silica gel 60 F_254_ aluminum plates (Merck). Visualization was accomplished by UV light. Column chromatography was performed on silica gel (60 Å, 40−63 μm, ROCC). The yield of the products refers to chromatographically and spectroscopically homogeneous materials.

Melting points were recorded with a Fisher Digital Melting Point Analyzer Model 355 apparatus. The infrared spectra were recorded in hexachlorobutadiene (4000−2000 cm^−1^) and paraffin oil (2000−450 cm^−1^) with an FTIR Perkin-Elmer Spectrum 100 spectrometer.

^1^H, ^13^C, and ^31^P NMR spectra were recorded with Bruker Avance 300 or Bruker Avance 500 spectrometers in CDCl_3_, DMSO-*d*_6_, and MeOD-*d*_4._ Chemical shifts (δ) are reported in ppm and coupling constants (*J*) in Hz. The proton (CDCl_3_ δ = 7.26 ppm, DMSO-*d*_6_ δ = 2.50 ppm, MeOD-*d*_4_ δ = 3.31 ppm, AcOD-*d*_4_ δ = 11.65 ppm) and carbon signals (CDCl_3_ δ = 77.16 ppm, DMSO-*d*_6_ δ = 39.52 ppm, MeOD-*d*_4_ δ = 49.00 ppm, AcOD-*d*_4_ δ = 178.99 ppm) for residual nondeuterated solvents were used as an internal reference for ^1^H and ^13^C NMR spectra, respectively. ^1^H NMR were recorded at 500 and 300 MHz and ^13^C NMR spectra at 125.7 and 75.5 MHz. ^31^P NMR spectra were recorded at 121 and 202 MHz with H_3_PO_4_ (85%) as an external standard (H_3_PO_4_ δ_P_ = 0.00 ppm). The multiplicity is assigned as follows: s – singlet, d (for ^1^H NMR) and D (for ^13^C NMR) – doublet, t – triplet, q – quartet, m – multiplet. Nontrivial peak assignments were confirmed by ^1^H,^1^H–COSY, ^1^H,^1^H-HMBC, and/or ^1^H,^13^C-HSQC 2D NMR experiments for representative products of each compound class.

Crystallographic diffraction data were collected with a NoniusKappa CCD diffractometer (Mo Kα, λ = 0.71073 Å) equipped with a low-temperature Oxford Cryosystems Cryostream Plus device.

HPLC analysis was performed using an Agilent Technologies 1200 Series system equipped with an XBridge C18 column, 4.6 × 150 mm, particle size 3.5 μm, with a flow rate of 1 mL/min, using eluent A–0.1% TFA/H_2_O with 5 vol % MeCN and eluent B–MeCN as the mobile phase. The wavelength of detection was 260 nm. Gradient: 30–95% B 5 min, 95% B 5 min, 95–30% B 2 min. LC–MS spectra were recorded with a Waters Acquity UPLC system equipped with an Acquilty UPLC BEH C18 1.7 μm, 2.1 × 50 mm column, using 0.1% TFA/H_2_O and MeCN as the mobile phase. HRMS analyses were performed on an Agilent 1290 Infinity series UPLC system equipped with an Extend C18 RRHD 2.1 × 50 mm, 1.8 μm column, connected to an Agilent 6230 TOF LC–MS mass spectrometer.

### General procedures and product characterization

The synthesis and characterization of the starting materials **1** and **5** and of 2,6-bistriazolylpurine derivative **6d** have been reported earlier [22].

#### General procedure for the S_N_Ar–Arbuzov reaction: synthesis of 9-alkyl-2-chloro-9*H*-purine C6-phosphonates **2**

**Diethyl (2-chloro-9-heptyl-9*H*-purin-6-yl)phosphonate (2a):** 2,6-Dichloro-9-heptyl-9*H*-purine (**1**, 1.03 g, 3.59 mmol, 1.0 equiv) was dissolved in P(OEt)_3_ (12 mL) and stirred for 3 h at 160 °C (HPLC control). Then, the solution was cooled to room temperature, hexane (40 mL) was added, and the mixture was left in the freezer (−20 °C) for 10 h. The precipitated colorless crystals of **2a** were filtered and washed with cold hexane (4 × 5 mL). Colorless crystals (1.15 g, 82%). mp 57–59 °C; IR ν̃_max_ (cm^−1^): 2924, 2858, 1334, 1243, 1021, 981; ^1^H NMR (CDCl_3_, 300 MHz) δ 8.18 (s, 1H, H–C(8)), 4.41 (quintet, ^3^*J* = 7.1 Hz, 4H, 2×H_2_C–O–P), 4.25 (t, ^3^*J* = 7.2 Hz, 2H, –CH_2_(1’)–), 2.02–1.77 (m, 2H, –CH_2_(2’)–), 1.40 (t, ^3^*J* = 7.1 Hz, 6H, 2×(–CH_3_)), 1.35–1.10 (m, 8H, 4×(–CH_2_–)), 0.85 (t, ^3^*J* = 6.6 Hz, 3H, –CH_3_(7’)); ^13^C NMR (CDCl_3_, 75.5 MHz) δ 154.3 (D, ^3^*J*_C–P_ = 11.7 Hz), 154.2 (D, ^3^*J*_C–P_ = 7.7 Hz), 152.5 (D, ^1^*J*_C–P_ = 203.6 Hz), 147.5, 134.3 (D, ^2^*J*_C–P_ = 21.4 Hz), 64.2 (D, ^2^*J*_C–P_ = 6.1 Hz), 44.2, 31.5, 29.8, 28.6, 26.5, 22.5, 16.4 (D, ^3^*J*_C–P_ = 6.2 Hz), 14.4; ^31^P NMR (CDCl_3_, 121 MHz) δ 5.3; HRMS-ESI (*m*/*z*): [M + H]^+^ calcd for C_16_H_27_ClN_4_O_3_P, 389.1504; found, 389.1508.

#### General procedure for the synthesis of 9-alkyl-2,6-bistriazolyl-9*H*-purine derivatives **6**

**Dimethyl 1,1'-(9-heptyl-9*H*-purine-2,6-diyl)bis(1*H*-1,2,3-triazole-4-carboxylate) (6a):** CuI (0.06 g, 0.30 mmol, 0.12 equiv) was added to a stirred solution of 2,6-diazido-9-heptyl-9*H*-purine (**5**, 0.76 g, 2.53 mmol, 1.0 equiv) in DCM (35 mL), followed by the addition of triethylamine (0.39 mL, 2.78 mmol, 1.1 equiv), methyl propiolate (0.68 mL, 7.59 mmol, 3.0 equiv), and acetic acid (0.16 mL, 2.78 mmol, 1.1 equiv). The reaction mixture was stirred for 2 h at room temperature. Then, the mixture was washed with brine (1 × 7 mL) and an aqueous solution of NaHS (2 × 5 mL). The inorganic phase was back-extracted with DCM (2 × 3 mL). The organic phase was collected, dried over anhydrous Na_2_SO_4_, filtered through Celite^®^, and evaporated under reduced pressure. Silica gel column chromatography (DCM/MeCN, gradient: 20→33%) provided the product **6a** (0.91 g, 76%) as a brown amorphous solid. *R*_f_ 0.20 (DCM/MeCN 4:1); HPLC: *t*_R_ 5.72 min; IR ν̃_max_ (cm^−1^): 2953, 2930, 1728, 1434, 1223, 1025, 774; ^1^H NMR (500 MHz, CDCl_3_) δ 9.63, 9.25 (2s, 2H, 2×H–C(triazole)), 8.40 (s, 1H, H–C(8)), 4.47 (t, ^3^*J* = 7.0 Hz, 2H, H_2_–C(1’)), 4.08 (s, 6H, 2×OMe), 2.10–1.93 (m, 2H, H_2_–C(2’)), 1.49–1.15 (m, 8H, 4×(–CH_2_–)), 0.85 (t, ^3^*J* = 6.9 Hz, 3H, H_3_–C(7’)); ^13^C NMR (125.7 MHz, CDCl_3_) δ 160.8, 160.5, 156.2, 148.5, 148.0, 144.8, 140.6, 140.5, 128.4, 127.5, 122.9, 52.74, 52.66, 45.1, 31.6, 29.9, 28.7, 26.7, 22.6, 14.1; HRMS-ESI (*m*/*z*): [M + H]^+^ calcd for C_20_H_25_N_10_O_4_, 469.2055; found, 469.2022.

#### General procedure for the S_N_Ar–Arbuzov reaction: synthesis of 9-alkyl-2-triazolyl-9*H*-purine C6-phosphonates **4**

**Methyl 1-(6-(diethoxyphosphoryl)-9-heptyl-9*H*-purin-2-yl)-1*H*-1,2,3-triazole-4-carboxylate (4a):** Dimethyl 1,1'-(9-heptyl-9*H*-purine-2,6-diyl)bis(1*H*-1,2,3-triazole-4-carboxylate) (**6a**, 0.20 g, 0.43 mmol, 1.0 equiv) was dissolved in P(OEt)_3_ (2 mL) and stirred for 3 hours at 160 °C. Then, the solution was cooled to room temperature, hexane (10 mL) was added, and the mixture was left in the freezer (−20 °C) for 10 h. The brown solids were filtered, washed with cold hexane (4 × 5 mL), then dissolved from the filter with DCM (10 mL) and purified by silica gel column chromatography (DCM/MeOH, gradient: 3→5%). Orange powder (0.148 g, 72%). *R*_f_ 0.2 (DCM/MeOH 25:1); IR ν̃_max_ (cm^−1^): 2980, 2930, 1250, 1143, 1102, 990; ^1^H NMR (500 MHz, CDCl_3_) δ 9.19 (s, 1H, H–C(triazole)), 8.32 (s, 1H, H–C(8)), 4.53–4.42 (m, 4H, 2×H_2_C–O–P), 4.40 (t, ^3^*J* = 7.3 Hz, 2H, H_2_–C(1’)), 4.00 (s, 3H, H_3_C–O–CO), 2.07–1.84 (m, 2H, H_2_–C(2’)), 1.45 (t, ^3^*J* = 7.1 Hz, 6H, 2×(–CH_3_)), 1.37–1.15 (m, 8H, 4×(–CH_2_–)), 0.85 (t, ^3^*J* = 6.9 Hz, 3H, H_3_–C(7’)); ^13^C NMR (125.7 MHz, CDCl_3_) δ 160.9, 154.0 (D, ^3^*J*_C–P_ = 11.1 Hz), 152.4 (D, ^1^*J*_C–P_ = 220.6 Hz), 148.6, 148.3 (D, ^3^*J*_C–P_ = 23.4 Hz), 140.3, 135.3 (D, ^2^*J*_C–P_ = 20.8 Hz), 127.4, 64.5 (D, ^2^*J*_C–P_ = 6.2 Hz), 52.6, 44.6, 31.6, 29.9, 28.7, 26.7, 22.7, 16.6 (D, ^3^*J*_C–P_ = 5.9 Hz), 14.1; ^31^P NMR (202 MHz, CDCl_3_) δ 5.3; HRMS-ESI (*m*/*z*): [M + H]^+^ calcd for C_20_H_30_N_7_O_5_P, 480.2119; found, 480.2121.

## Supporting Information

File 1 Full experimental procedures and copies of the ^1^H, ^13^C, and ^31^P NMR spectra.

File 2 Cif file for compound **4d**.
