# Supplementary material for: 1,2,3-Triazoles as leaving groups in SNAr–Arbuzov reactions: synthesis of C6-phosphonated purine derivatives
Source: Beilstein J Org Chem. 2021 Jan 20;17:193–202. doi: 10.3762/bjoc.17.19 (PMC7849246; doi:10.3762/bjoc.17.19)
Supplement: File 1 — Full experimental procedures and copies of the 1H, 13C, and 31P NMR spectra. [file Beilstein_J_Org_Chem-17-193-s001.pdf]

BEILSTEIN JOURNAL OF ORGANIC CHEMISTRY

Supporting Information

for

1,2,3-Triazoles as leaving groups in S_NAr–Arbuzov reactions: synthesis of C6-phosphonated purine derivatives

Kārlis-Ēriks Kriķis, Irina Novosjolova, Anatoly Mishnev and Māris Turks

Beilstein J. Org. Chem. **2021,** *17,* 193–202. doi:10.3762/bjoc.17.19

Full experimental procedures and copies of the ^{1}H, ^{13}C, and ^{31}P NMR spectra

License and Terms: This is a supporting information file under the terms of the Creative Commons Attribution License (https://creativecommons.org/licenses/by/4.0). Please note that the reuse, redistribution and reproduction in particular requires that the author(s) and source are credited and that individual graphics may be subject to special legal provisions.
The license is subject to the *Beilstein Journal of Organic Chemistry* terms and conditions: (https://www.beilstein-journals.org/bjoc/terms)

Cleavage of the dialkyl (2-chloro-9-heptyl-9*H*-purin-6-yl)phosphonate 2 ester groups and analysis by ^{1}H and ^{31}P NMR spectroscopy

Reaction of diethyl (2-chloro-9-heptyl-9*H*-purin-6-yl)phosphonate (2a) with NaN_3: General procedure for the ester group cleavage from the phosphonates with NaN_3 and NaCl: Diethyl (2-chloro-9-heptyl-9*H*-purin-6-yl)phosphonate (**2a**, 8.04 mg, 0.0207 mmol, 1.0 equiv), 1,2,3-trimethoxybenzene (1.68 mg) as a standard, and NaN_3 (3.78 mg, 3.0 equiv) were weighted in an NMR tube and DMSO-d_6 (0.6 mL) was added. The NMR tube was heated at 90 °C using an oil bath. Then, ^{1}H NMR (500 MHz) spectra, with a D1 relaxation time of 20 s, and ^{31}P NMR (202 MHz) spectra, with and without ^{1}H coupling, were recorded after 15, 30, and 45 min as well as after 1, 24, and 48 h.

Reaction of diisopropyl (2-chloro-9-heptyl-9*H*-purin-6-yl)phosphonate (2b) with NaN_3:

Performed according to the general procedure for the ester group cleavage from the phosphonates with NaN_3 and NaCl: diisopropyl (2-chloro-9-heptyl-9*H*-purin-6-yl)phosphonate (**2b**, 3.76 mg, 0.0090 mmol, 1.0 equiv), 1,2,3-trimethoxybenzene (1.26 mg), and NaN_3 (1.90 mg, 3.0 equiv). NMR spectra were recorded after 15, 30, and 45 min as well as after 1, 24, and 48 h.

Reaction of diethyl (2-chloro-9-heptyl-9*H*-purin-6-yl)phosphonate (2a) with NaCl: Performed according to the general procedure for the ester group cleavage from the phosphonates with NaN_3 and NaCl: diethyl (2-chloro-9-heptyl-9*H*-purin-6-yl)phosphonate (**2a**, 7.03 mg, 0.0181 mmol, 1.0 equiv), 1,2,3-trimethoxybenzene (2.74 mg), and NaCl (3.20 mg, 3.0 equiv). NMR spectra were recorded after 15, 30, 60, 75, and 90 min as well as after 48 h.

Reaction of diisopropyl (2-chloro-9-heptyl-9*H*-purin-6-yl)phosphonate (2b) with NaCl:

Performed according to the general procedure for the ester group cleavage from the phosphonates with NaN_3 and NaCl: diisopropyl (2-chloro-9-heptyl-9*H*-purin-6-yl)phosphonate (**2b**, 4.04 mg, 0.0097 mmol, 1.0 equiv), 1,2,3-trimethoxybenzene (1.39 mg), and NaCl (1.59 mg, 3.0 equiv). NMR spectra were recorded after 15, 30, 60, 75, and 90 min as well as after 48 h.

Table S1: Reaction of diethyl (2-chloro-9-heptyl-9*H*-purin-6-yl)phosphonate **2a** and NaN_3 or NaCl.

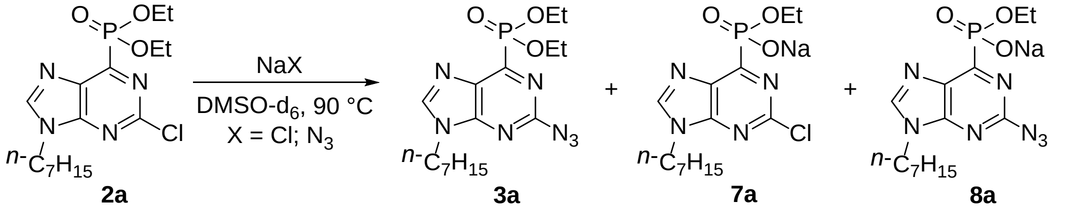

reaction with NaN_3 (3.0 equiv)			reaction with NaCl (3.0 equiv)		
time	compound	%[a]	time	compound	%[a]
15 min	**2a**	51	1 h	**2a**	95
	**3a**	8			
	**7a**	29		**7a**	5
	**8a**	3			
30 min	**2a**	37	1 h 30 min	**2a**	94
	**3a**	9			
	**7a**	40		**7a**	6
	**8a**	5			
24 h	**7a**	58	48 h	**2a**	1
	**8a**	31			
48 h	**7a**	48		**7a**	98
	**8a**	39			

[a]The percentage of the compound in the reaction mixture was calculated from the integral value of the purine H–C(8) signals (^1H NMR, 500 MHz, DMSO-d_6), using 1,2,3-trimethoxybenzene as a standard. Where the sum of the percentages of the major intermediates was less than 100%, other unidentifiable byproducts were observed in the reaction mixture.

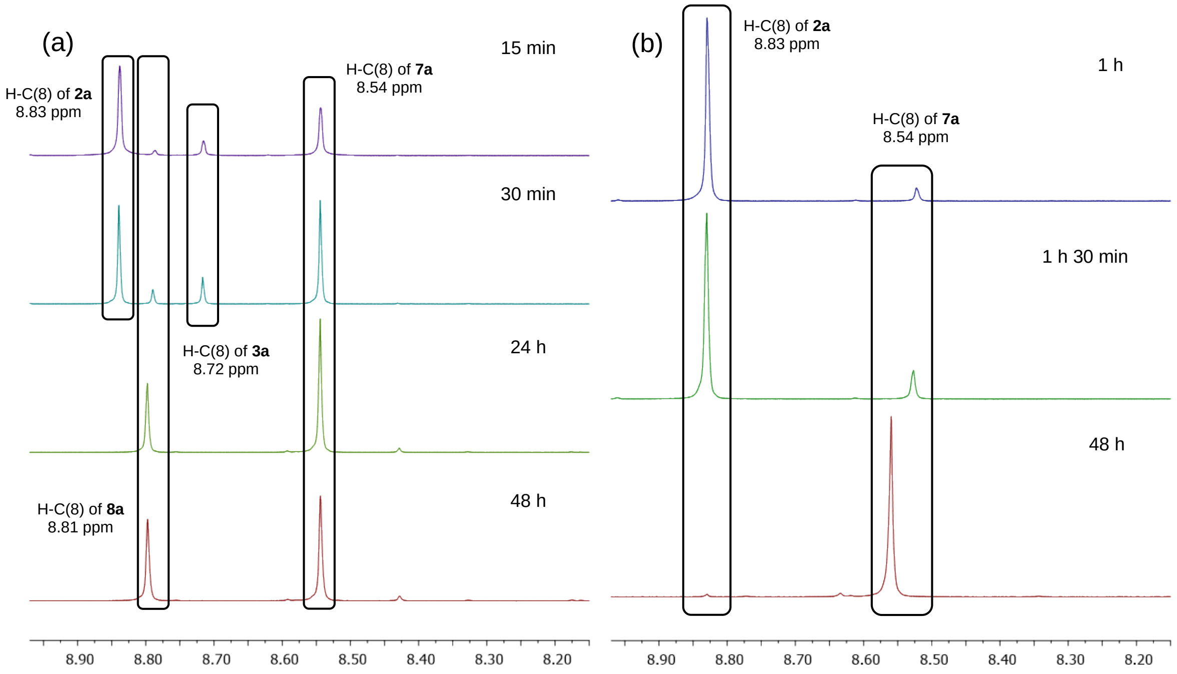

Figure S1: ^1H NMR analysis (500 MHz, DMSO-d_6) of two parallel reactions between diethyl (2-chloro-9-heptyl-9*H*-purin-6-yl)phosphonate (**2a**) and NaN_3 (a) or NaCl (b).

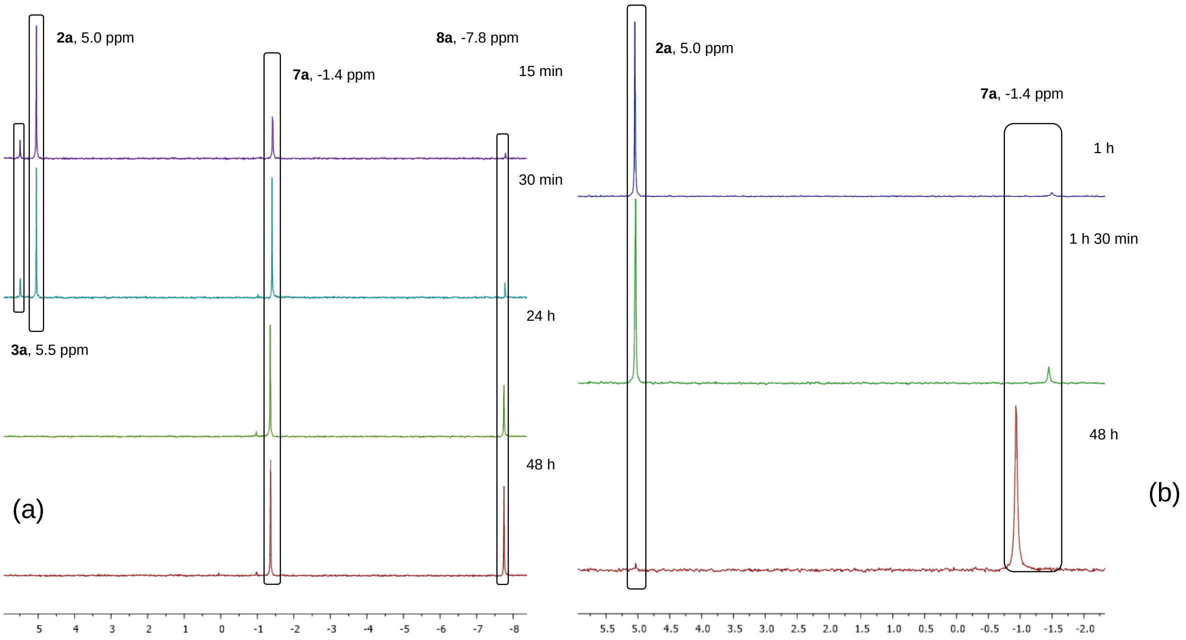

Figure S2: ^{31}P NMR analysis (202 MHz, DMSO-d_6) of two parallel reactions between diethyl (2-chloro-9-heptyl-9*H*-purin-6-yl)phosphonate (**2a**) and NaN_3 (a) or NaCl (b).

Table S2: Reaction of diisopropyl (2-chloro-9-heptyl-9*H*-purin-6-yl)phosphonate (**2b**) and NaN_3 or NaCl.

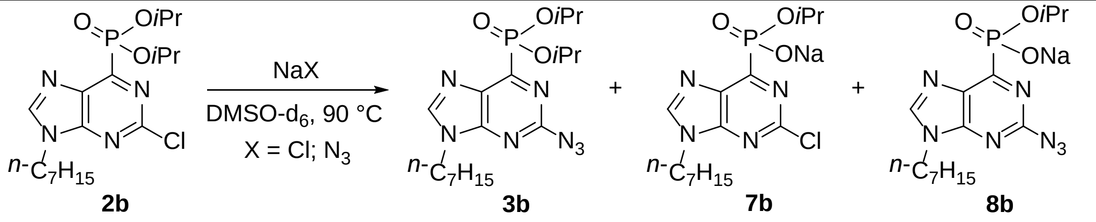

reaction with NaN_3 (3.0 equiv)			reaction with NaCl (3.0 equiv)		
time	compound	%[a]	time	compound	%[a]
15 min	**2b**	98	1 h	**2b**	100
	**7b**	1			
30 min	**2b**	97			
	**7b**	1			
24 h	**2b**	41	48 h	**2b**	95
	**3b**	25			
	**7b**	31			
	**8b**	13			
48 h	**2b**	5		**7b**	5
	**3b**	18			
	**7b**	32			
	**8b**	45			

[a]The percentage of the compound in the reaction mixture was calculated from the integral value of the purine H–C(8) signals (^1H NMR, 500 MHz, DMSO-d_6), using 1,2,3-trimethoxybenzene as a standard. Where the sum of the percentages of the major intermediates was less than 100%, other unidentifiable byproducts were observed in the reaction mixture.

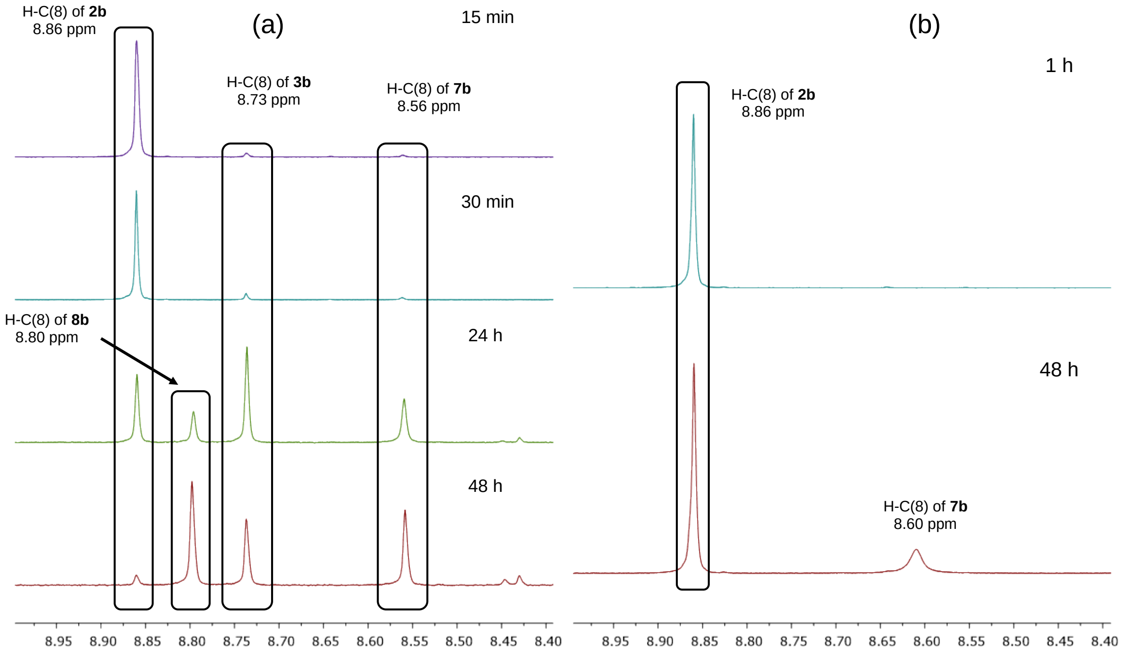

Figure S3: ^1H NMR analysis (500 MHz, DMSO-d_6) of two parallel reactions between diisopropyl (2-chloro-9-heptyl-9*H*-purin-6-yl)phosphonate (**2b**) and NaN_3 (a) or NaCl (b).

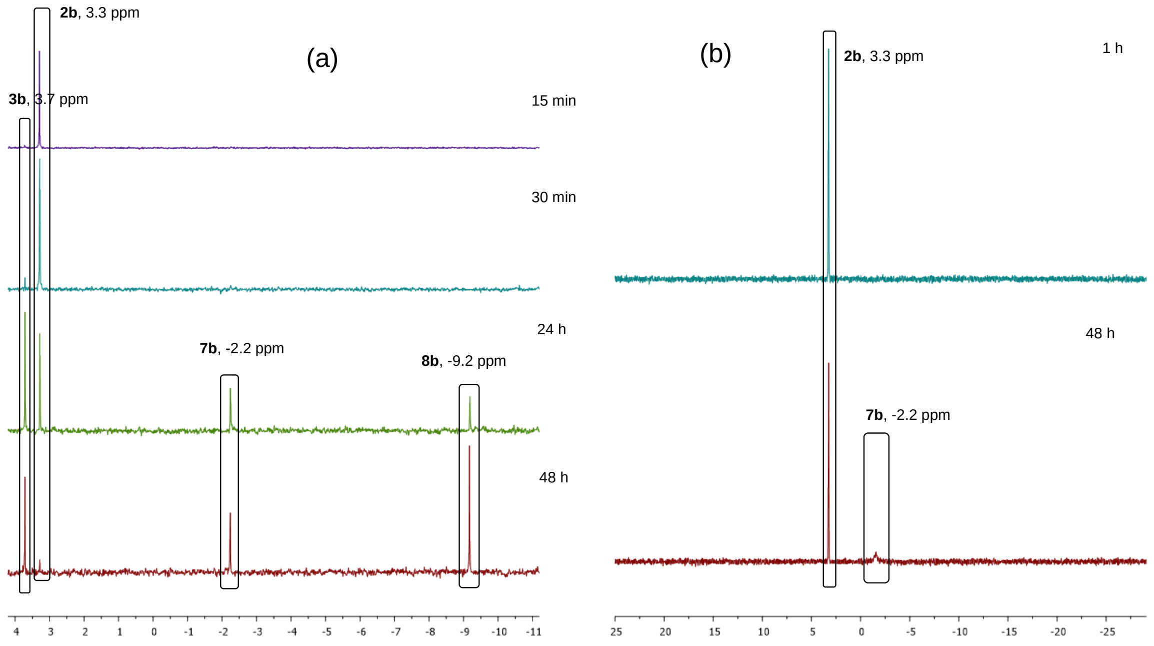

Figure S4: ^{31}P NMR analysis (202 MHz, DMSO-d_6) of two parallel reactions between diisopropyl (2-chloro-9-heptyl-9*H*-purin-6-yl)phosphonate (**2b**) and NaN_3 (a) or NaCl (b).

Experimental part

General information

Commercially available reagents were used as received. The reactions and the purity of the synthesized compounds were monitored by HPLC and TLC analysis using silica gel 60 F_{254} aluminum plates (Merck). Visualization was accomplished by UV light. Column chromatography was performed on silica gel (60 Å, 40−63 μm, ROCC). The yield of the products refers to chromatographically and spectroscopically homogeneous materials.

Melting points were recorded with a Fisher Digital Melting Point Analyzer Model 355 apparatus. The infrared spectra were recorded in hexachlorobutadiene (4000−2000 cm^{-1}) and paraffin oil (2000−450 cm^{-1}) with an FTIR Perkin-Elmer Spectrum 100 spectrometer.

^{1}H, ^{13}C, and ^{31}P NMR spectra were recorded with Bruker Avance 300 or Bruker Avance 500 spectrometers in $CDCl_3$, DMSO-d_6, and MeOD-d_4. Chemical shifts (δ) are reported in ppm and coupling constants (*J*) in Hz. The proton ($CDCl_3$ δ = 7.26 ppm, DMSO-d_6 δ = 2.50 ppm, MeOD-d_4 δ = 3.31 ppm, AcOD-d_4 δ = 11.65 ppm) and carbon signals ($CDCl_3$ δ = 77.16 ppm, DMSO-d_6 δ = 39.52 ppm, MeOD-d_4 δ = 49.00 ppm, AcOD-d_4 δ□= 178.99 ppm) for residual nondeuterated solvents were used as an internal reference for ^{1}H and ^{13}C NMR spectra, respectively. ^{1}H NMR were recorded at 500 and 300 MHz and ^{13}C NMR spectra at 125.7 and 75.5 MHz. ^{31}P NMR spectra were recorded at 121 and 202 MHz with H_3PO_4 (85%) as an external standard (H_3PO_4 δ_P = 0.00 ppm). The multiplicity is assigned as follows: s–singlet, d (for ^{1}H NMR) and D (for ^{13}C NMR)–doublet, t–triplet, q–quartet, m–multiplet. Nontrivial peak assignments were confirmed by ^{1}H,^{1}H–COSY, ^{1}H,^{1}H-HMBC, and/or ^{1}H,^{13}C-HSQC 2D NMR experiments for representative products of each compound class.

Crystallographic diffraction data were collected with a NoniusKappa CCD diffractometer (Mo Kα, λ = 0.71073 Å) equipped with a low-temperature Oxford Cryosystems Cryostream Plus device.

HPLC analysis was performed using an Agilent Technologies 1200 Series system equipped with an XBridge C18 column, 4.6×150 mm, particle size 3.5 μm, with a flow rate of 1 mL/min, using eluent A–0.1% TFA/H_2O with 5 vol % MeCN and eluent B–MeCN as the mobile phase. The wavelength of detection was 260 nm. Gradient: 30–95% B 5 min, 95% B 5 min, 95–30% B 2 min. LC–MS spectra were recorded with a Waters Acquity UPLC system equipped with an Acquilty UPLC BEH C18 1.7 μm,

2.1×50 mm column, using 0.1% TFA/H_2O and MeCN as the mobile phase. HRMS analyses were performed on an Agilent 1290 Infinity series UPLC system equipped with an Extend C18 RRHD 2.1×50 mm, 1.8 µm column, connected to an Agilent 6230 TOF LC–MS mass spectrometer.

General procedures and product characterization

The synthesis and characterization of the starting materials **1** and **5** and of the 2,6-bistriazolylpurine derivative **6d** have been reported earlier [1].

Synthesis of the 9-alkyl 2-chloro-9*H*-purine C6-phosphonates 2

Diethyl (2-chloro-9-heptyl-9*H*-purin-6-yl)phosphonate (2a)

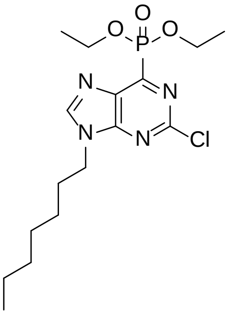

<u>General procedure for the S_NAr–Arbuzov reaction</u>: 2,6-Dichloro-9-heptyl-9*H*-purine (**1**, 1.03 g, 3.59 mmol, 1.0 equiv) was dissolved in $P(OEt)_3$ (12 mL) and stirred for 3 h at 160 °C (HPLC control). Then, the solution was cooled to room temperature, hexane (40 mL) was added, and the mixture was left in the freezer (−20 °C) for 10 h. Precipitated colorless crystals were filtered and washed with cold hexane (4×5 mL). Colorless crystals (1.15 g, 82%). M_p = 57–59 °C. IR ṽ (cm^{-1}): 2924, 2858, 1334, 1243, 1021, 981. ^1H NMR (300 MHz, $CDCl_3$) δ (ppm): 8.18 (s, 1H, H-C(8)), 4.41 (quintet, 4H, ^3J = 7.1 Hz, 2×H_2C-O-P), 4.25 (t, 2H, ^3J = 7.2 Hz, -CH_2-(1')), 2.02–1.77 (m, 2H, -CH_2-(2')), 1.40 (t, 6H, ^3J = 7.1 Hz, 2×(-CH_3)), 1.35–1.10 (m, 8H, 4×(-CH_2-)), 0.85 (t, 3H, ^3J = 6.6 Hz, -CH_3(7')). ^{13}C NMR (75.5 MHz, $CDCl_3$) δ (ppm): 154.3 (D, $^3J_{C-P}$ = 11.7 Hz), 154.2 (D, $^3J_{C-P}$ = 7.7 Hz), 152.5 (D, $^1J_{C-P}$ = 203.6 Hz), 147.5, 134.3 (D, $^2J_{C-P}$ = 21.4 Hz), 64.2 (D, $^2J_{C-P}$ = 6.1 Hz), 44.2, 31.5, 29.8, 28.6, 26.5, 22.5, 16.4 (D, $^3J_{C-P}$ = 6.2 Hz), 14.4. ^{31}P NMR (121 MHz, $CDCl_3$) δ (ppm): 5.3. HRMS ESI (m/z): calculated [$C_{16}H_{26}ClN_4O_3P+H^+$] 389.1504, found 389.1508.

Diisopropyl (2-chloro-9-heptyl-9*H*-purin-6-yl)phosphonate (2b)

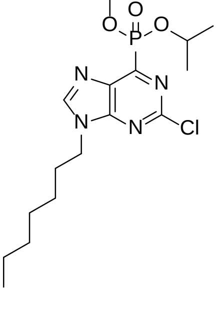

Obtained according to the general procedure of the S_NAr–Arbuzov reaction: 2,6-dichloro-9-heptyl-9*H*-purine (0.47 g, 1.64 mmol, 1.0 equiv), $P(OiPr)_3$ (5 mL, *c* = 0.1 g/1.0 mL). Reaction conditions: 12 h, 160 °C. An excess of phosphite was distilled under reduced pressure (3 mbar) at 50 °C. Silica gel column chromatography (DCM/MeCN, gradient: 10%→50%) provided the product **2b** (0.63 g, 92%) as a

greenish oil. R_f = 0.14 (DCM/MeCN 5:1). M_p = 56–58 °C. IR ṽ (cm^{-1}): 2980, 2930, 1250, 1143, 1102, 990. ^1H NMR (500 MHz, $CDCl_3$) δ (ppm): 8.17 (s, 1H, H-C(8)), 5.02 (octet, 2H, ^3J = 6.1 Hz, 2×(-HC-O-P)), 4.25 (t, 2H, ^3J = 7.2 Hz, H_2-C(1’)), 1.90 (quintet, 2H, ^3J = 6.2 Hz, H_2-C(2’)), 1.45 (d, 6H, ^3J = 7.1 Hz, 2×(-CH_3)), 1.38 (d, 6H, ^3J = 6.2 Hz, 2×(-CH_3)), 1.35–1.19 (m, 8H, 4×(-CH_2-)), 0.86 (t, 3H, ^3J = 6.9 Hz, H_3-C(7’)). ^{13}C NMR (125.7 MHz, $CDCl_3$) δ (ppm): 154.4 (D, $^3J_{C-P}$ = 11.3 Hz), 154.1 (D, $^3J_{C-P}$ = 25.4 Hz), 153.8 (D, $^1J_{C-P}$ = 220.6 Hz), 147.2, 134.4 (D, $^2J_{C-P}$ = 21.4 Hz), 73.3 (D, $^2J_{C-P}$ = 6.1 Hz), 44.3, 31.7, 29.7, 28.6, 26.7, 24.4 (D, $^3J_{C-P}$ = 3.5 Hz), 23.9 (D, $^3J_{C-P}$ = 5.5 Hz), 22.6, 14.4. ^{31}P NMR (202 MHz, $CDCl_3$) δ (ppm): 3.5. HRMS ESI (m/z): calculated [$C_{18}H_{30}ClN_4O_3P+H^+$] 417.1817; found 417.1825.

Synthesis of monoethyl (2-azido-9-heptyl-9*H*-purin-6-yl)phosphonate (9a)

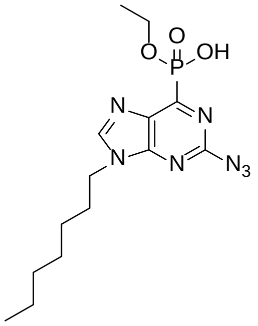

A solution of diethyl (2-chloro-9-heptyl-9*H*-purin-6-yl)phosphonate (**2a**, 0.40 g, 1.03 mmol, 1.0 equiv) and NaN_3 (0.50 g, 7.70 mmol, 7.5 equiv) in DMSO (5 mL) was stirred for 48 h at 90 °C. Then, it was cooled to room temperature and lyophilized. The obtained foam was dissolved in water (10 mL), conc H_3PO_4 was added (1 mL), and this was put in the refrigerator (5 °C) for 12 h. The formed precipitate was filtered through Celite® and washed with cold water (2×1 mL). Then, the residue was washed off the filter with MeOH (20 mL) and evaporated under reduced pressure. An orange powder (0.106 g, 28%). IR ṽ (cm^{-1}): 3094, 2928, 2135, 1602, 1337, 1200, 1084, 1027, 997, 954. ^1H NMR (500 MHz, MeOD-d_4) δ (ppm): 9.07, (s, 1H, H-C(8)), 4.43 (t, 2H, ^3J = 7.2 Hz, -CH_2-(1’)) 4.22 (quintet, 2H, ^3J = 7.1 Hz, H_2C-O-P), 1.93 (quintet, 2H, ^3J = 6.9 Hz, -CH_2-(2’)), 1.40–1.19 (m, 8H, 4×(-CH_2-)) 1.29 (t, 3H, ^3J = 7.1 Hz, (-CH_3)), 0.85 (t, 3H, ^3J = 6.6 Hz, -CH_3(7’)). ^{13}C NMR (125.7 MHz, MeOD-d_4) δ (ppm): 159.1 (D, $^3J_{C-P}$ = 20.8 Hz), 157.3 (D, $^1J_{C-P}$ = 208.3 Hz), 154.1 (D, $^3J_{C-P}$ = 8.9 Hz), 147.9, 128.1 (D, $^2J_{C-P}$ = 20.1 Hz), 64.4 (D, $^2J_{C-P}$ = 6.0 Hz), 46.1, 32.8, 30.2, 29.7, 27.5, 23.6, 16.9 (D, $^3J_{C-P}$ = 6.3 Hz), 14.4. ^{31}P NMR (202 MHz, MeOD-d_4) δ (ppm): 1.8. HRMS ESI (m/z): calculated [$C_{14}H_{22}N_7O_3P+H^+$] 368.1595; found 368.1608.

Monoisopropyl (2-azido-9-heptyl-9*H*-purin-6-yl)phosphonate (9b)

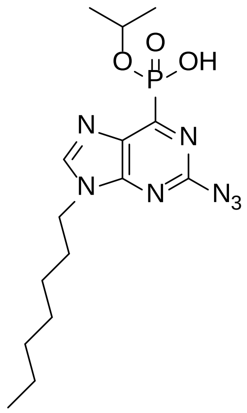

A solution of diisopropyl (2-chloro-9-heptyl-9*H*-purin-6-yl)phosphonate (**2b**, 0.56 g, 1.34 mmol, 1.0 equiv) and NaN_3 (0.88 g, 13.4 mmol, 10.0 equiv) in DMSO (5 mL) was stirred for 48 h at 100 °C. Then, it was cooled to room temperature and lyophilized. The obtained foam was dissolved in water (15 mL), conc H_3PO_4 was added (0.4 mL), and this was put in the refrigerator (5 °C) for 12 h. The cloudy solution was filtered through Celite® and the precipitate washed with cold water (2×1 mL). Then, the residue was washed off the filter with MeOH (20 mL), evaporated under reduced pressure, and purified by silica gel column chromatography (DCM/MeOH/TFA 50:1:0.2→15:1:0.05). An orange solid (0.12 g, 23%). R_f = 0.14 (DCM:MeOH:TFA = 15:1:0.06). IR ṽ (cm^{-1}): 2930, 2862, 2123, 1604, 1345, 1164, 1050, 1002, 842, 704. ^{1}H NMR (500 MHz, MeOD-d_4) δ (ppm): 8.96, (s, 1H, H-C(8)), 4.83 (octet, 1H, ^3J = 6.6 Hz, (-HC-O-P)), 4.31 (t, 2H, ^3J = 7.2 Hz, H_2-C(1')), 1.91 (quintet, 2H, ^3J = 7.2 Hz, H_2-C(2')), 1.39–1.21 (m, 8H, 4×(-CH_2-)), 1.28 (d, 6H, ^3J = 6.2 Hz, 2×(-CH_3)), 0.85 (t, 3H, ^3J = 6.8 Hz, H_3-C(7')). ^{13}C NMR (125.7 MHz, MeOD-d_4) δ (ppm): 158.8 (D, $^3J_{C\text{-}P}$ = 21.4 Hz), 157.7 (D, $^1J_{C\text{-}P}$ = 209.5 Hz), 154.3 (D, $^3J_{C\text{-}P}$ = 10.4 Hz), 148.1, 129.1 (D, $^2J_{C\text{-}P}$ = 19.5 Hz), 73.9 (D, $^2J_{C\text{-}P}$ = 6.0 Hz), 45.8, 32.7, 30.3, 29.7, 27.5, 24.5 (D, $^3J_{C\text{-}P}$ = 3.8 Hz), 23.6, 14.3. ^{31}P NMR (202 MHz, MeOD-d_4) δ (ppm): 1.9. HRMS ESI (m/z): calculated [$C_{15}H_{24}N_7O_3P+H^+$] 382.1751; found 382.1753.

(2-Chloro-9-heptyl-9*H*-purin-6-yl)phosphonic acid (10)

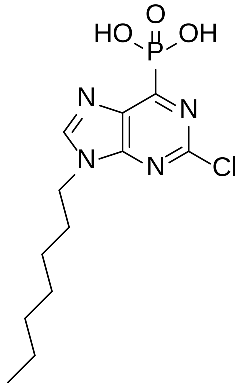

Diethyl (2-chloro-9-heptyl-9*H*-purin-6-yl)phosphonate (**2a**, 100 mg, 0.26 mmol, 1.0 equiv) was dissolved in absolute DCM (7 mL) and cooled to −78 °C. Trimethylsilyl iodide (0.07 mL, 0.52 mmol, 2.0 equiv) was added, and the mixture was stirred for 3 min, after which the flask was lifted out of the acetone–dry ice bath, and the mixture was stirred for 15 min at room temperature. Methanol (3 mL) was added to quench the reaction and the solution was evaporated under reduced pressure. The orange residue was recrystallized from methanol (15 mL), and the product **10** (36 mg, 42%) was obtained as colorless crystals. M_p ≥ 216 $^{°C}$ starts to decompose. IR ṽ (cm^{-1}): 3089, 2927, 2860, 1584, 1375, 1240, 1230, 1060, 908, 866. ^{1}H NMR (300 MHz, AcOD-d_4) δ (ppm): 9.82, (s, 1H, H-C(8)), 4.63 (t, 2H, ^3J = 7.4 Hz, H_2-C(1')), 2.21–2.15 (m, 2H, H_2-C(2')), 1.58–1.30 (m,

8H, 4×(-CH_2-)), 0.96 (t, 3H, ^3J = 6.8 Hz, H_3-C(7')). ^{13}C NMR (75.5 MHz, AcOD-d_4) δ (ppm): 160.7 (D, $^1J_{C\text{-}P}$ = 207.5 Hz), 158.9 (D, $^3J_{C\text{-}P}$ = 21.5 Hz), 152.1 (D, $^3J_{C\text{-}P}$ = 8.5 Hz), 147.2, 124.9 (D, $^2J_{C\text{-}P}$ = 17.0 Hz), 48.0, 33.1, 30.2, 30.1, 27.7, 24.0, 14.9. ^{31}P NMR (202 MHz, AcOD-d_4) δ (ppm): -0.9. HRMS ESI (m/z): calculated [$C_{12}H_{18}ClN_4O_3P+H^+$] 333.0878; found 333.0877.

Synthesis of the 9-alkyl 2,6-bistriazolyl-9*H*-purine derivatives 6

Dimethyl 1,1'-(9-heptyl-9*H*-purine-2,6-diyl)bis(1*H*-1,2,3-triazole-4-carboxylate) (6a)

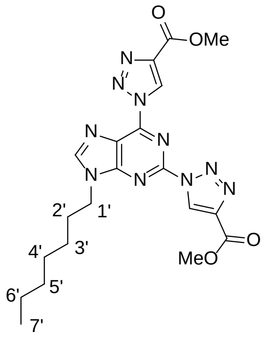

<u>General procedure for the synthesis of the 2,6-bistriazolylpurine derivatives **6**</u>: CuI (0.06 g, 0.30 mmol, 0.12 equiv) was added to a stirred solution of 2,6-diazido-9-heptyl-9*H*-purine (**5**, 0.76 g, 2.53 mmol, 1.0 equiv) in DCM (35 mL), followed by an addition of triethylamine (0.39 mL, ρ = 0.73 g/mL, 2.78 mmol, 1.1 equiv), methylpropiolate (0.68 mL, ρ = 0.95 g/mL, 7.59 mmol, 3.0 equiv), and acetic acid (0.16 mL, ρ = 1.05 g/mL, 2.78 mmol, 1.1 equiv). The reaction mixture was stirred for 2 h at room temperature. Then, the mixture was washed with brine (1×7 mL) and an aqueous solution of NaHS (2×5 mL). The inorganic phase was back-extracted with DCM (2×3 mL). The organic phase was collected, dried over anhydrous Na_2SO_4, filtered through Celite®, and evaporated under reduced pressure. Silica gel column chromatography (DCM/MeCN, gradient: 5:1→3:1) provided the product **6a** (0.91 g, 76%) as a brown amorphous solid. R_f = 0.20 (DCM/MeCN = 4:1). HPLC: t_R = 5.72 min. IR ṽ (cm^{-1}): 2953, 2930, 1728, 1434, 1223, 1025, 774. ^{1}H NMR (500 MHz, $CDCl_3$) δ (ppm): 9.63, 9.25 (2s, 2H, 2×H-C(triazole)), 8.40 (s, 1H, H-C(8)), 4.47 (t, 2H, ^3J = 7.0 Hz, H_2-C(1')), 4.08 (s, 6H, 2×OMe), 2.10–1.93 (m, 2H, H_2-C(2')), 1.49–1.15 (m, 8H, 4×(-CH_2-)), 0.85 (t, 3H, ^3J = 6.9 Hz, H_3-C(7')). ^{13}C NMR (125.7 MHz, $CDCl_3$) δ (ppm): 160.8, 160.5, 156.2, 148.5, 148.0, 144.8, 140.6, 140.5, 128.4, 127.5, 122.9, 52.74, 52.66, 45.1, 31.6, 29.9, 28.7, 26.7, 22.6, 14.1. HRMS ESI (m/z): calculated [$C_{20}H_{24}N_{10}O_4+H^+$] 469.2055; found 469.2022.

2,6-Bis(4-(3-cyanopropyl)-1*H*-1,2,3-triazol-1-yl)-9-heptyl-9*H*-purine (6b)

Obtained according to the general procedure for the synthesis of the 2,6-bistriazolylpurine derivatives: diazide **5** (0.70 g, 2.33 mmol, 1.0 equiv), 5-hexynenitrile (0.84 mL, ρ = 0.81 g/mL, 7.00 mmol, 3.0 equiv), CuI (0.06 g, 0.29 mmol, 0.12 equiv), triethylamine (0.39 mL, ρ = 0.73 g/mL, 2.80 mmol, 1.1 equiv), acetic acid (0.17 mL, ρ

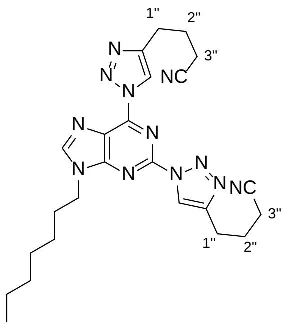

= 1.05 g/mL, 2.80 mmol, 1.1 equiv), and DCM (35 mL). Reaction time: 9 h. Recrystallization from MeOH gave the product **6b** (0.83 g, 73%) as a colorless powder. HPLC: t_R = 5.36 min. IR ṽ (cm^{-1}): 3150, 3082, 2952, 2242, 1618, 1474, 1215, 1031, 989. ^{1}H NMR (300 MHz, $CDCl_3$) δ (ppm): 8.90, 8.54 (2s, 2H, 2×H-C(triazole)), 8.30 (s, 1H, H-C(8)), 4.42 (t, 2H, ^3J = 7.2 Hz, H_2-C(1')), 3.06–3.03 (m, 4H, 2×H_2-C(1'')), 2.50 (t, 4H, ^3J = 7.3 Hz, 2×H_2-C(3'')), 2.30–2.19 (m, 4H, 2×H_2-C(2'')), 2.08–1.87 (m, 2H, H_2-C(2')), 1.47–1.15 (m, 8H, 4×(-CH_2-)), 0.86 (t, 3H, ^3J = 6.9 Hz, H_3-C(7')). ^{13}C NMR (75.5 MHz, $CDCl_3$) δ (ppm): 155.9, 148.6, 147.4, 146.6, 146.4, 145.4, 122.2, 121.7, 121.2, 119.4, 119.3, 44.9, 31.7, 29.9, 28.8, 26.7, 24.90, 24.87, 24.3 (2C),[1] 22.6, 16.67, 16.65, 14.1. HRMS ESI (m/z): calculated [$C_{24}H_{30}N_{12}+H^+$] 487.2789; found 487.2789.

2,6-Bis(4-butyl-1*H*-1,2,3-triazol-1-yl)-9-heptyl-9*H*-purine (6c)

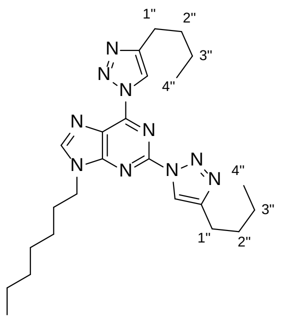

Obtained according to the general procedure for the synthesis of the 2,6-bistriazolylpurine derivatives: diazide **5** (0.613 g, 2.04 mmol, 1.0 equiv), 1-hexyne (0.84 mL, ρ = 0.81 g/mL, 7.00 mmol, 3.0 equiv), CuI (0.08 g, 0.40 mmol, 0.12 equiv), triethylamine (0.41 mL, ρ = 0.73 g/mL, 2.93 mmol, 1.1 equiv), acetic acid (0.17 mL, ρ = 1.05 g/mL, 2.93mmol, 1.1 equiv), and DCM (35 mL). Reaction time: 3.5 h. Recrystallization from a hexane/EtOH mixture gave the product **6c** (0.54 g, 57%) as a sand-colored powder. HPLC: t_R = 5.36 min. IR ṽ (cm^{-1}): 2957, 2925, 2859, 1607, 1589, 1448, 1222, 1032. ^{1}H NMR (500 MHz, $CDCl_3$) δ (ppm): 8.79, 8.44 (2s, 2H, 2×H-C(triazole)), 8.26 (s, 1H, H-C(8)), 4.41 (t, 2H, ^3J = 7.3 Hz, H_2-C(1')), 2.88, 2.84 (2t, 4H, ^3J = 7.7 Hz, 2×H_2-C(1'')), 2.04–1.94 (m, 2H, H_2-C(2')), 1.82–1.70 (m, 4H, 2×H_2-C(2'')), 1.44 (sextet, 4H, ^3J = 7.5 Hz, 2×H_2-C(3'')), 1.40–1.19 (m, 8H, 4×(-CH_2-)), 0.96 (t, 6H, ^3J = 7.5 Hz, 2×H_3-C(4'')), 0.85 (t, 3H, ^3J = 6.8 Hz, H_3-C(7')). ^{13}C NMR (125.7 MHz, $CDCl_3$) δ (ppm): 155.9, 149.4, 149.2, 148.9, 147.0, 145.6, 122.0, 120.9, 120.4, 44.8, 31.7, 31.5, 31.4, 29.9, 28.8, 26.7, 25.5, 25.4, 22.6, 22.42, 22.41, 14.1, 13.94, 13.93. HRMS ESI (m/z): calculated [$C_{24}H_{36}N_{10}+H^+$] 465.3197; found 465.3192.

[1] The signal was assigned from the HSQC spectrum.

2,6-Bis(4-(4-cyanophenyl)-1*H*-1,2,3-triazol-1-yl)-9-heptyl-9*H*-purine (6e)

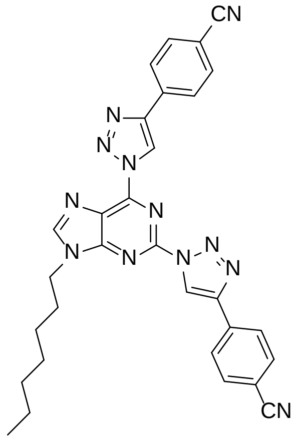

Obtained according to the general procedure for the synthesis of the 2,6-bistriazolylpurine derivatives: diazide **5** (0.50 g, 1.67 mmol, 1.0 equiv), 4-cyanophenylacetylene (0.64 g, 5.00 mmol, 3.0 equiv), CuI (0.04 g, 0.20 mmol, 0.12 equiv), triethylamine (2.55 mL, ρ = 0.74 g/mL, 1.83 mmol, 1.1 equiv), acetic acid (0.84 mL, ρ = 1.05 g/mL, 1.83 mmol, 1.1 equiv), and DCM (35 mL). Reaction time: 12 h. Recrystallization from EtOH provided the product **6e** (0.65 g, 70%) as a slightly yellow powder. HPLC: t_R = 7.73 min. ^{1}H NMR (500 MHz, $CDCl_3$) δ (ppm): 9.51 (s, 1H, H-C(triazole)), 9.08 (s, 1H, H-C(triazole)), 8.35 (s, 1H, H-C(8)), 8.17 (d, 2H, ^3J = 8.4 Hz, 2×H-C(Ar)), 8.14 (d, 2H, ^3J = 8.4 Hz, 2×H-C(Ar)), 7.81, 7.79 (2d, 4H, ^3J = 8.4 Hz, 4×H-C(Ar)), 4.48 (t, 2H, ^3J = 7.3 Hz, H_2-C(1')), 2.05 (quintet, 2H, ^3J = 7.3 Hz, H_2-C(2')), 1.46–1.38 (m, 4H, 2×(-CH_2-)), 1.33–1.27 (m, 4H, 2×(-CH_2-)), 0.88 (t, 3H, ^3J = 7.0 Hz, H_3-C(7')). ^{13}C NMR (126 MHz, $CDCl_3$) δ (ppm): 156.1, 148.6, 147.7, 146.9, 146.6, 145.2, 134.3, 133.9, 133.03, 133.01, 126.9, 126.7, 122.4, 121.3, 120.3, 118.8, 118.7, 112.6, 112.4, 45.1, 31.7, 23.0, 28.8, 26.8, 22.7, 14.2. HRMS ESI (m/z): calculated [$C_{30}H_{26}N_{12}+H^+$] 555.2476, found 555.2469.

9-Heptyl-2,6-bis(4-(4-methoxyphenyl)-1*H*-1,2,3-triazol-1-yl)-9*H*-purine (6f)

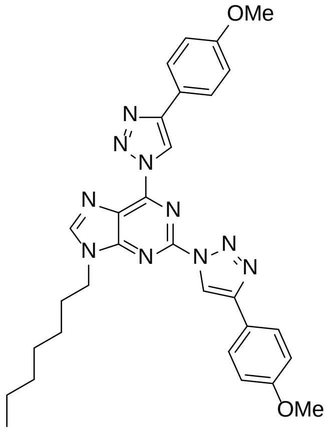

Obtained according to the general procedure for the synthesis of the 2,6-bistriazolylpurine derivatives: diazide **5** (0.3 g, 1.00 mmol, 1.0 equiv), 4-methoxyphenylacetylene (0.40 g, 3.00 mmol, 3.0 equiv), CuI (0.02 g, 0.12 mmol, 0.12 equiv), triethylamine (0.20 mL, ρ = 0.73 g/mL, 1.1 mmol, 1.1 equiv), acetic acid (0.08 mL, ρ = 1.05 g/mL, 1.1 mmol, 1.1 equiv), and DCM (35 mL). Reaction time: 1.5 h. Recrystallization from MeOH provided the product **6f** (0.26 g, 46%) as a slightly yellow powder. IR ṽ (cm^{-1}): 2950, 2930, 1617, 1470, 1448, 1250, 1003. ^{1}H NMR (500 MHz, $CDCl_3$) δ (ppm): 9.31, 8.92 (2s, 2H, H-C(triazole)), 8.30 (s, 1H, H-C(8)), 7.92, 7.89 (2d, 4H, ^3J = 8.6 Hz, 4×H-C(Ar)), 6.99, 6.97 (2d, 4H, ^3J = 8.6 Hz, 4×H-C(Ar)), 4.41 (t, 2H, ^3J = 7.3 Hz, H_2-C(1')), 3.85 (s, 6H, 2×-OMe), 2.01 (quintet, 2H, ^3J = 7.0 Hz, H_2-C(2')), 1.44–1.22 (m, 8H, 4×(-CH_2-)), 0.87 (t, 3H, ^3J = 6.9 Hz, H_3-C(7')). ^{13}C NMR (125.7 MHz, $CDCl_3$) δ (ppm): 160.3, 160.1, 155.8, 148.7, 148.2,

148.0, 147.2, 145.3, 127.7, 127.5, 122.6, 122.2, 122.1, 118.5, 118.1, 114.5, 114.4, 55.5 (2C),[2] 44.9, 31.7, 29.9, 28.8, 26.7, 22.6, 14.1. HRMS ESI (m/z): calculated [$C_{30}H_{32}N_{10}O_2$+H^+] 565.2782; found 565.2754.

2,6-Bis(4-(4-chlorophenyl)-1*H*-1,2,3-triazol-1-yl)-9-heptyl-9*H*-purine (6g)

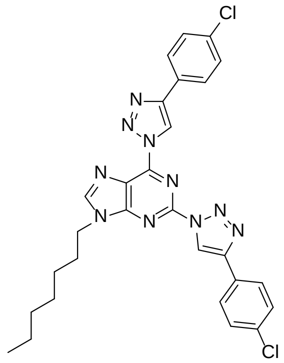

Obtained according to the general procedure for the synthesis of the 2,6-bistriazolylpurine derivatives: diazide **5** (0.65 g, 2.17 mmol, 1.0 equiv), 4-chlorophenylacetylene (0.88 g, 6.50 mmol, 3.0 equiv), CuI (0.05 g, 0.28 mmol, 0.12 equiv), triethylamine (0.33 mL, ρ = 0.74 g/mL, 2.38 mmol, 1.1 equiv), acetic acid (0.08 mL, ρ = 1.05 g/mL, 2.38 mmol, 1.1 equiv), and DCM (35 mL). Reaction time: 15 h. Silica gel column chromatography (MeCN in DCM, gradient 2%→11%) provided the product **6g** (0.43 g, 35%) as a colorless powder. R_f = 0.2 (DCM/MeCN = 10:1). IR ṽ (cm^{-1}): 2960, 2923, 1590, 1445, 1406, 1228, 1093, 1004, 808. ^{1}H NMR (500 MHz, $CDCl_3$) δ (ppm): 9.31, 8.92 (2s, 2H, 2×H-C(triazole)), 8.30 (s, 1H, H-C(8)), 7.96, 7.93 (2d, 4H, ^3J = 8.4 Hz, 4×H-C(Ar)), 7.46, 7.45 (2d, 4H, ^3J = 8.4 Hz, 4×H-C(Ar)), 4.45 (t, 2H, ^3J = 7.3 Hz, H_2-C(1’)), 2.03 (quintet, 2H, ^3J = 7.3 Hz, H_2-C(2’)), 1.53–1.18 (m, 8H, 4×(-CH_2-)), 0.88 (t, 3H,^3J = 6.9 Hz, H_3-C(7’)). ^{13}C NMR (125.7 MHz, $CDCl_3$) δ (ppm): 155.9, 148.6, 147.5, 147.4, 147.3, 145.3, 135.0, 134.7, 129.4, 129.3, 128.4, 128.1, 127.6, 127.5, 122.3, 119.8, 119.1, 45.0, 31.7, 30.0, 28.8, 26.8, 22.7, 14.1. HRMS ESI (m/z): calculated [$C_{28}H_{26}Cl_2N_{10}$+H^+] 573.1792; found 573.1783.

2,6-Bis(4-(2-fluorophenyl)-1*H*-1,2,3-triazol-1-yl)-9-heptyl-9*H*-purine (6h)

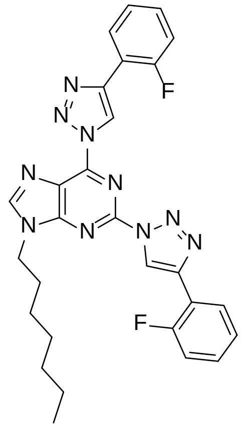

Obtained according to the general procedure for the synthesis of the 2,6-bistriazolylpurine derivatives: diazide **5** (0.30 g, 1.00 mmol, 1.0 equiv), 2-fluorophenylacetylene (0.34 mL, ρ = 1.06 g/mL 3.00 mmol, 3.0 equiv), CuI (0.02 g, 0.12 mmol, 0.12 equiv), triethylamine (0.20 mL, ρ = 0.73 g/mL, 1.1 mmol, 1.1 equiv), acetic acid (0.08 mL, ρ = 1.05 g/mL, 1.1 mmol, 1.1 equiv), and DCM (35 mL). Reaction time: 3 h. Recrystallization from MeOH provided the product **6h** (0.313 g, 58%) as a grey powder. HPLC: t_R = 8.31 min. IR ṽ (cm^{-1}): 2927, 2856, 1620, 1472, 1228, 1008. ^{1}H NMR (500 MHz, $CDCl_3$) δ (ppm): 9.38 (brs,

[2] The signal was assigned using the HSQC spectra.

1H, H-C(triazole)), 9.04 (d, 1H, $^{5}J_{H-F}$ = 3.4 Hz, H-C (triazole)), 8.51–8.37 (m, 2H, 2×H-C(Ar)), 8.33 (s, 1H, H-C(8)), 7.46–7.34 (m, 2H, 2×H-C(Ar)), 7.34–7.28 (m, 2H, 2×H-C (Ar)), 7.24–7.16 (m, 2H, 2×H-C (Ar)), 4.46 (t, 2H ^{3}J = 7.4 Hz, H_2-C(2')), 2.17–1.95 (m, 2H, H_2-C(2')), 1.49–1.22 (m, 8H, 4×(-CH_2-)), 0.87 (t, 3H, ^{3}J = 6.9 Hz, H_3-C(7')). ^{13}C NMR (125.7 MHz, $CDCl_3$) δ (ppm): 159.7 (D, $^{1}J_{C-F}$ = 249.4 Hz), 159.6 (D, $^{1}J_{C-F}$ = 249.4 Hz), 156.0, 148.7, 147.5, 145.5, 141.9, 141.8 (D, $^{3}J_{C-F}$ = 2.2 Hz), 130.3 (D, $^{3}J_{C-F}$ = 8.3 Hz), 130.1 (D, $^{3}J_{C-F}$ = 8.3 Hz), 128.6 (D, $^{3}J_{C-F}$ = 2.8 Hz), 128.4 (D, $^{3}J_{C-F}$ = 2.9 Hz), 124.8 (2C),[3] 122.5, 122.4 (D, $^{4}J_{C-F}$ = 13.1 Hz), 122.0 (D, $^{4}J_{C-F}$ = 13.0 Hz), 118.1 (D, $^{2}J_{C-F}$ = 12.9 Hz), 117.8 (D,$^{2}J_{C-F}$ = 12.7 Hz), 116.0 (D, $^{2}J_{C-F}$ = 21.3 Hz), 115.9 (D, $^{2}J_{C-F}$ = 22.0 Hz), 44.9, 31.7, 30.0, 28.8, 26.7, 22.6, 14.1. HRMS ESI (m/z): calculated [$C_{28}H_{26}F_2N_{10}+H^+$] 541.2383; found 565.2754.

9-Heptyl-2,6-bis(4-(pyridn-2-yl)-1*H*-1,2,3-triazol-1-yl)-9*H*-purine (6i)

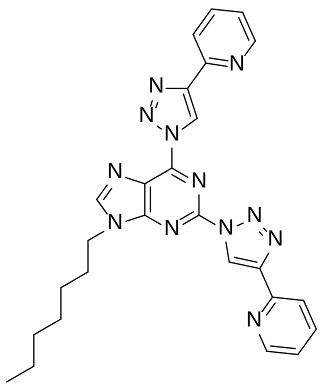

Obtained according to the general procedure for the synthesis of the 2,6-bistriazolylpurine derivatives: diazide **5** (0.40 g, 1.33 mmol, 1.0 equiv), 2-ethynyl pyridine (0.40 mL, ρ = 1.02 g/mL, 4.00 mmol, 3.0 equiv), CuI (0.03 g, 0.16 mmol, 0.12 equiv), triethylamine (0.20 mL, ρ = 0.74 g/mL, 1.48 mmol, 1.1 equiv), acetic acid (0.09 mL, ρ = 1.05 g/mL, 1.48 mmol, 1.1 equiv), and DCM (35 mL). Reaction time: 9 h. Silica gel column chromatography (DCM/MeCN, gradient 2%→11%) provided the product **6i** (0.235 g, 35%) as a grey powder. R_f = 0.20 (DCM/MeCN, 10:1). HPLC: t_R = 5.31 min. IR ṽ (cm^{-1}): 2958, 2932, 1618, 1474, 1460, 1215, 1031, 1004, 989. ^{1}H NMR (500 MHz, $CDCl_3$) δ (ppm): 9.59, 9.30 (2s, 2H, 2×H-C(triazole)), 8.72 – 8.60 (m, 2H, 2×H-C(Py)) 8.34 (s, 1H, H-C(8)), 8.23 (t, 2H, ^{3}J = 7.8 Hz, 2×H-C(Py)), 7.82 (t, 2H, ^{3}J = 7.8 Hz, 2×H-C(Py)), 7.34–7.27 (m, 2H, 2×H-C(Py)), 4.44 (t, 2H, ^{3}J = 7.3 Hz, H_2-C(1')), 2.01 (quintet, 2H, ^{3}J = 7.3 Hz, H_2-C(2')), 1.43–1.19 (m, 8H, 4×(-CH_2-)), 0.85 (t, 3H, ^{3}J = 6.9 Hz, H_3-C(7')). ^{13}C NMR (125.7 MHz, $CDCl_3$) δ (ppm): 156.1, 149.9, 149.7, 149.6, 149.3, 148.8, 148.7, 148.5, 147.7, 145.4, 137.1, 137.0, 123.6, 123.4, 122.6, 121.8, 121.5, 121.1, 120.9, 44.9, 31.7, 29.9, 28.8, 26.8, 22.7, 14.1. HRMS ESI (m/z): calculated [$C_{26}H_{26}N_{12}+H^+$] 507.2476; found 507.2499.

[3] The signal was assigned using HSQC and HMBC spectra.

Synthesis of the 9-alkyl 2-triazolyl-9*H*-purine C6-phosphonates 4

Methyl 1-(6-(diethoxyphosphoryl)-9-heptyl-9*H*-purin-2-yl)-1*H*-1,2,3-triazole-4-carboxylate (4a)

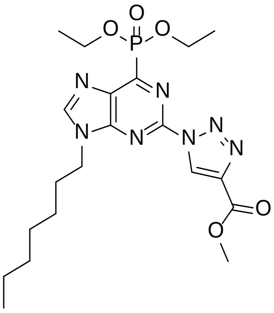

General procedure for the S_NAr–Arbuzov reaction: Dimethyl 1,1'-(9-heptyl-9*H*-purine-2,6-diyl)bis(1*H*-1,2,3-triazole-4-carboxylate) (**6a**, 0.20 g, 0.43 mmol, 1.0 equiv) was dissolved in $P(OEt)_3$ (2 mL), and this was stirred for 3 h at 160 °C. Then, the solution was cooled to room temperature, hexane (10 mL) was added, and the mixture was left in the freezer (−20 °C) for 10 h. Brown solids were filtered, washed with cold hexane (4×5 mL), then dissolved from the filter with DCM (10 mL), and purified by silica gel column chromatography (DCM/MeOH, gradient 3→5%). Orange powder (0.148 g, 72%). R_f = 0.2 (DCM/MeOH, 20:1). IR ṽ (cm^{-1}): 2980, 2930, 1250, 1143, 1102, 990. ^1H NMR (500 MHz, $CDCl_3$) δ (ppm): 9.19 (s, 1H, H-C(triazole)), 8.32 (s, 1H, H-C(8)), 4.53–4.42 (m, 4H, 2×H_2C-O-P), 4.40 (t, 2H ^3J = 7.3 Hz, H_2-C(1’)), 4.00 (s, 3H, H_3C-O-CO), 2.07–1.84 (m, 2H, H_2-C(2’)), 1.45 (t, 6H, ^3J = 7.1 Hz, 2×(-CH_3)), 1.37–1.15 (m, 8H, 4×(-CH_2-)), 0.85 (t, 3H, ^3J = 6.9 Hz, H_3-C(7’)). ^{13}C NMR (125.7 MHz, $CDCl_3$) δ (ppm): 160.9, 154.0 (D, $^3J_{C-P}$ = 11.1 Hz), 152.4 (D, $^1J_{C-P}$ = 220.6 Hz), 148.6, 148.3 (D, $^3J_{C-P}$ = 23.4 Hz), 140.3, 135.3 (D, $^2J_{C-P}$ = 20.8 Hz), 127.4, 64.5 (D, $^2J_{C-P}$ = 6.2 Hz), 52.6, 44.6, 31.6, 29.9, 28.7, 26.7, 22.7, 16.6 (D, $^3J_{C-P}$ = 5.9 Hz), 14.1. ^{31}P NMR (202 MHz, $CDCl_3$) δ (ppm): 5.3. HRMS ESI (m/z): calculated [$C_{20}H_{30}N_7O_5P+H^+$] 480.2119, found 480.2121.

Diethyl (2-(4-(3-cyanopropyl)-1*H*-1,2,3-triazol-1-yl)-9-heptyl-9*H*-purin-6-yl)phosphonate (4b)

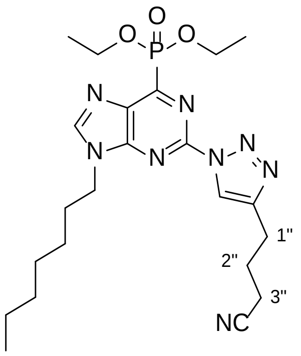

Obtained according to the general procedure of the S_NAr–Arbuzov reaction: 2,6-bis(4-(3-cyanopropyl)-1*H*-1,2,3-triazol-1-yl)-9-heptyl-9*H*-purine (**6b**, 150 mg, 0.31 mmol, 1.0 equiv) and $P(OEt)_3$ (1.50 mL). Reaction time: 20 h. An excess of $P(OEt)_3$ was distilled under a vacuum of 5 mbar for 4–5 hours at 50 °C. Then, this was purified using preparative reversed-phase chromatography. A colorless oil (66 mg, 44%). IR ṽ (cm^{-1}): 2955, 2931, 2859, 2246, 1579, 1465, 1252, 1238, 1020. ^1H NMR (300 MHz, $CDCl_3$) δ (ppm): 8.50 (s, 1H, H-C(triazole)), 8.28 (s, 1H, H-C(8)), 4.47 (quintet, 4H, ^3J = 7.4 Hz, 2×H_2C-O-P), 4.38 (t, 2H ^3J = 7.2 Hz, H_2-C(1’)), 3.00 (t, 2H, ^3J = 7.3 Hz, H_2-C(1’’)), 2.48 (t, 2H, ^3J = 7.3 Hz, H_2-C(3’’)), 2.17 (quintet, 2H, ^3J = 7.3 Hz, H_2-C(2’’)), 2.04–1.88 (m,

2H, H_2-C(2')), 1.45 (t, 6H, ^3J = 7.4 Hz, 2×H_3-C), 1.40–1.16 (m, 8H, 4×(-CH_2-)), 0.85 (t, 3H, ^3J = 6.7 Hz, H_3-C(7')). ^{13}C NMR (125.7 MHz, $CDCl_3$) δ (ppm): 154.0 (D, $^3J_{C-P}$ = 11.1 Hz), 152.1 (D, $^1J_{C-P}$ = 221.1 Hz), 148.8 (D, $^3J_{C-P}$ = 23.4 Hz), 148.7, 148.2, 134.9 (D, $^2J_{C-P}$ = 21.4 Hz), 121.2, 119.4 64.4 (D, $^2J_{C-P}$ = 6.0 Hz), 44.5, 31.7, 31.5, 29.9, 28.8, 26.7, 24.9, 24.3, 22.6, 16.6 (D, $^3J_{C-P}$ = 5.9 Hz), 14.1. ^{31}P NMR (202 MHz, $CDCl_3$) δ (ppm): 5.7. HRMS ESI (m/z): calculated [$C_{22}H_{33}N_8O_3P+H^+$] 489.2486; found 489.2478.

Diethyl (2-(4-buthyl-1*H*-1,2,3-triazol-1-yl)-9-heptyl-9*H*-purin-6-yl)phosphonate (4c)

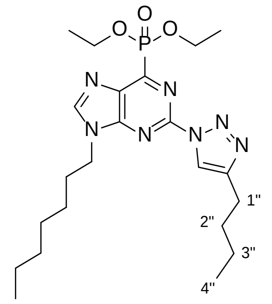

Obtained according to the general procedure of the S_NAr–Arbuzov reaction: 2,6-bis(4-butyl-1*H*-1,2,3-triazol-1-yl)-9-heptyl-9*H*-purine (**6c**, 150 mg, 0.32 mmol, 1.0 equiv) and $P(OEt)_3$ (1.50 mL). Reaction time: 14 h. An excess of $P(OEt)_3$ was distilled under a vacuum of 5 mbar for 4-5 hours at 50 °C. Then, purified using preparative reverse phase chromatography. A colorless oil (46 mg, 30%). IR ṽ (cm^{-1}): 2980, 2930, 1457, 1254, 1017. ^{1}H NMR (500 MHz, $CDCl_3$) δ (ppm): 8.41 (s, 1H, H-C(triazole)), 8.26 (s, 1H, H-C(8)), 4.51–4.41 (m, 4H, 2×H_2C-O-P), 4.38 (t, 2H ^3J = 7.2 Hz, H_2-C(1')), 2.82 (t, 2H, ^3J = 7.6 Hz, H_2-C(1'')), 2.01–1.89 (m, 2H, H_2-C(2')), 1.73 (quintet, 2H, ^3J = 7.6 Hz, H_2-C(2'')), 1.50–1.38 (m, 8H, 2×-H_3C, H_2-C(3'')), 1.38–1.19 (m, 8H, 4×(-CH_2-)), 0.95 (t, 3H, ^3J = 7.4 Hz, 2×H_2-C(4'')), 0.85 (t, 3H, ^3J = 6.8 Hz, H_3-C(7')). ^{13}C NMR (125.7 MHz, $CDCl_3$) δ (ppm): 154.0 (D, $^3J_{C-P}$ = 11.1 Hz), 152.4 (D, $^1J_{C-P}$ = 221.4 Hz), 149.1 (D, $^3J_{C-P}$ = 22.4 Hz), 149.0, 148.1, 134.7 (D, $^2J_{C-P}$ = 21.2 Hz), 120.5, 64.4 (D, $^2J_{C-P}$ = 5.9 Hz), 44.5, 31.7, 31.5, 29.9, 28.8, 26.7, 25.4, 22.6, 22.4, 16.6 (D, $^3J_{C-P}$ = 5.9 Hz), 14.1, 13.9. ^{31}P NMR (121 MHz, $CDCl_3$) δ (ppm): 5.9. HRMS ESI (m/z): calculated [$C_{22}H_{36}N_7O_3P+H^+$] 478.2690, found 478.2718.

Diethyl (9-heptyl-2-(4-phenyl-1*H*-1,2,3-triazol-1-yl)-9*H*-purin-6-yl)phosphonate (4d)

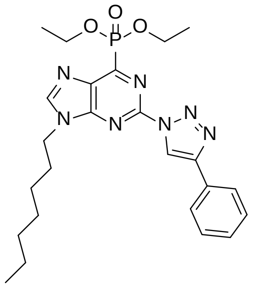

Obtained according to the general procedure of the S_NAr–Arbuzov reaction: 2,6-bis(4-phenyl-1*H*-1,2,3-triazol-1-yl)-9-heptyl-9*H*-purine (**6d**, 0.267 g, 0.53 mmol, 1.0 equiv) and $P(OEt)_3$ (4 mL). Reaction time: 6 h. Recrystallization from EtOH/hexane gave the product **4d** (0.20 g, 76%) as a sand-colored powder. For X-ray analysis, **4d** was crystallized from a hexane/DCM mixture by the slow-evaporation technique. M_p = 134–136 °C. IR ṽ (cm^{-1}): 2958, 2926, 1620, 1468, 1236, 1010. ^{1}H NMR

(500 MHz, $CDCl_3$) δ (ppm): 8.89 (s, 1H, H-C(triazole)), 8.29 (s, 1H, H-C(8)), 7.97–7.95 (m, 2H, 2×H-C(Ph)), 7.47 (t, 2H, ^3J = 7.6 Hz, 2×H-C(Ph)), 7.37 (t, 1H, ^3J = 7.4 Hz, H-C(Ph)), 4.49 (quintet, 4H, ^3J = 7.1 Hz, 2×(-H_2C-O-P)), 4.40 (t, 2H, ^3J = 7.4 Hz, H_2-C(1')), 2.04–1.91 (m, 2H, -CH_2- (2')), 1.46 (t, 6H, ^3J = 7.1 Hz, 2×(-CH_3)), 1.42–1.16 (m, 8H, 4×(-CH_2-)), 0.85 (t, 3H, ^3J = 6.9 Hz, -CH_3(7')). ^{13}C NMR (125.7 MHz, $CDCl_3$) δ (ppm): 154.0 (D, $^3J_{C\text{-}P}$ = 11.4 Hz), 152.1 (D, $^1J_{C\text{-}P}$ = 220.7 Hz), 148.9 (D, $^3J_{C\text{-}P}$ = 23.5 Hz), 148.2, 148.1, 134.9 (D, $^2J_{C\text{-}P}$ = 21.6 Hz), 130.1, 129.0, 128.7, 126.2, 119.1, 64.4 (D, $^2J_{C\text{-}P}$ = 5.9 Hz), 44.5, 31.7, 29.9, 28.8, 26.7, 22.6, 16.6 (D, $^3J_{C\text{-}P}$ = 6.1 Hz), 14.1. ^{31}P NMR (202 MHz, $CDCl_3$) δ (ppm): 5.8. HRMS ESI (m/z): calculated [$C_{24}H_{32}N_7O_3P+H^+$] 498.2377, found 498.2372.

Diethyl (2-(4-(4-cyanophenyl)-1*H*-1,2,3-triazol-1-yl)-9-heptyl-9*H*-purin-6-yl)phosphonate (4e)

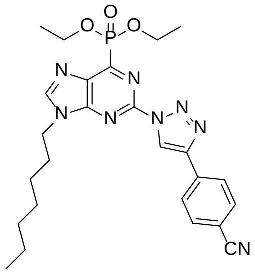

Obtained according to the general procedure of the S_NAr–Arbuzov reaction: 2,6-bis(4-(4-cyanophenyl)-1*H*-1,2,3-triazol-1-yl)-9-heptyl-9*H*-purine (**6e**, 1.05 g, 1.89 mmol, 1.0 equiv) and P(OEt)$_3$ (9 mL). Reaction time: 2 h. Recrystallization from hexane gave the product **4e** (0.81 g, 82%) as an orange powder. M_p = 146–147 °C. IR ṽ (cm^{-1}): 2933, 2857, 2223, 1459, 1242; 1018. ^{1}H NMR (300 MHz, $CDCl_3$) δ (ppm): 9.01 (s, 1H, H-C(triazole)), 8.31 (s, 1H, H-C(8)), 8.08 (d, 2H, ^3J = 8.4 Hz, 2×H-C(Ar)), 7.76 (d, 2H, ^3J = 8.4 Hz, 2×H-C(Ar)), 4.48 (quintet, 4H, ^3J = 7.3 Hz, 2×(-H_2C-O-P)), 4.41 (t, 2H, ^3J = 7.3 Hz, H_2-C(1')), 2.13–1.91 (m, 2H, H_2-C(2')), 1.46 (t, 6H, ^3J = 7.3 Hz, 2×(-CH_3)), 1.43–1.17 (m, 8H, 4×(-CH_2-)), 0.85 (t, 3H, ^3J = 7.0 Hz, H_3-C(7')). ^{13}C NMR (75.5 MHz, $CDCl_3$) δ (ppm): 154.0 (D, $^3J_{C\text{-}P}$ = 11.3 Hz), 152.1 (D, $^1J_{C\text{-}P}$ = 222.5 Hz), 148.6 (D, $^3J_{C\text{-}P}$ = 23.3 Hz), 148.4, 146.2, 135.1 (D, $^2J_{C\text{-}P}$ = 21.2 Hz), 134.5, 132.9, 126.5, 120.4, 118.8, 112.1, 64.4 (D, $^2J_{C\text{-}P}$ = 5.9 Hz), 44.6, 31.7, 29.9, 28.8, 26.7, 22.6, 16.6 (D, $^3J_{C\text{-}P}$ = 6.0 Hz), 14.1. ^{31}P NMR (202 MHz, $CDCl_3$) δ (ppm): 5.8. HRMS ESI (m/z): calculated [$C_{25}H_{31}N_8O_3P+H^+$] 523.2329, found 523.2319.

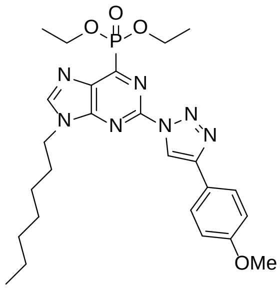

Diethyl (9-heptyl-2-(4-(4-methoxyphenyl)-1*H*-1,2,3-triazol-1-yl)-9*H*-purin-6-yl)phosphonate (4f)

Obtained according to the general procedure of the S_NAr–Arbuzov reaction: 9-heptyl-2,6-bis(4-(4-methoxyphenyl)-1*H*-1,2,3-triazol-1-yl)-9*H*-purine (**6f**, 90 mg, 0.16 mmol, 1.0 equiv) and P(OEt)$_3$ (2 mL). Reaction time: 23 h. Precipitated from

hexane (30 mL), then purified by silica gel column chromatography (DCM/MeCN, gradient: 8:1→4:1). An orange powder (34 mg, 40%). R_f = 0.17 (DCM/MeCN = 4: 1). IR ṽ (cm^{-1}): 2933, 2857, 2223, 1459, 1242; 1018. ^{1}H NMR (500 MHz, $CDCl_3$) δ (ppm): 8.81 (s, 1H, H-C(triazole)), 8.28 (s, 1H, H-C(8)), 7.90 (d, 2H, ^3J = 8.6 Hz, 2×H-C(Ar)), 7.01 (d, 2H, ^3J = 8.6 Hz, 2×H-C(Ar)), 4.49 (quintet, 4H, ^3J = 7.1 Hz, 2×(-H_2C-O-P)), 4.41 (t, 2H, ^3J = 7.2 Hz, H_2-C(1')), 3.87 (s, 3H, -OMe), 2.05–1.91 (m, 2H, H_2-C (2')), 1.47 (t, 6H, ^3J = 7.1 Hz, 2×(-CH_3)), 1.42–1.19 (m, 8H, 4×(-CH_2-)), 0.87 (t, 3H, ^3J = 6.9 Hz, -CH_3(7')). ^{13}C NMR (125.5 MHz, $CDCl_3$) δ (ppm): 160.1, 154.1 (D, $^3J_{C\text{-}P}$ = 10.9 Hz), 152.0 (D, $^1J_{C\text{-}P}$ = 220.7 Hz), 149.0 (D, $^3J_{C\text{-}P}$ = 23.2 Hz), 148.2, 148.0, 134.8 (D, $^2J_{C\text{-}P}$ = 21.4 Hz), 127.5, 122.7, 118.2, 114.5, 64.4 (D, $^2J_{C\text{-}P}$ = 5.7 Hz), 55.5, 44.5, 31.7, 29.9, 28.8, 26.7, 22.6, 16.6 (D, $^3J_{C\text{-}P}$ = 6.1 Hz), 14.1. ^{31}P NMR (202 MHz, $CDCl_3$) δ (ppm): 5.8. HRMS ESI (m/z): calculated [$C_{25}H_{34}N_7O_4P+H^+$] 528.2483; found 528.2465.

Diethyl (2-(4-(4-chlorophenyl)-1*H*-1,2,3-triazol-1-yl)-9-heptyl-9*H*-purin-6-yl)phosphonate (4g)

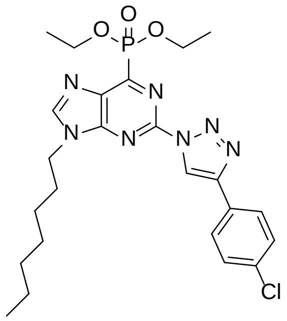

Obtained according to the general procedure of the S_NAr–Arbuzov reaction: 2,6-bis(4-(4-chlorophenyl)-1*H*-1,2,3-triazol-1-yl)-9-heptyl-9*H*-purine (**6g**, 90 mg, 0.16 mmol, 1.0 equiv) and $P(OEt)_3$ (9 mL). Reaction time: 9 h. Precipitated from hexane (20 mL) and then purified by silica gel column chromatography (DCM/MeCN, gradient: 5:1 → 2:1). An orange powder (68 mg, 80%). IR ṽ (cm^{-1}): 2933, 2857, 2223, 1459, 1242; 1018. ^{1}H NMR (500 MHz, $CDCl_3$) δ (ppm): 8.88 (s, 1H, H-C(triazole)), 8.33 (s, 1H, H-C(8)), 7.89 (d, 2H, ^3J = 8.0 Hz, 2×H-C(Ar)), 7.43 (d, 2H, ^3J = 8.0 Hz, 2×H-C(Ar)), 4.48 (quintet, 4H, ^3J = 7.1 Hz, 2×(-H_2C-O-P)), 4.40 (t, 2H, ^3J = 7.0 Hz, H_2-C(1')), 2.05–1.91 (m, 2H, -CH_2- (2')), 1.46 (t, 6H, ^3J = 7.1 Hz, 2×(-CH_3)), 1.40–1.19 (m, 8H, 4×(-CH_2-)), 0.85 (t, 3H, ^3J = 6.9 Hz, -CH_3(7')). ^{13}C NMR (125.5 MHz, $CDCl_3$) δ (ppm): 154.0 (D, $^3J_{C\text{-}P}$ = 11.1 Hz), 152.0 (D, $^1J_{C\text{-}P}$ = 220.4 Hz), 148.8 (D, $^3J_{C\text{-}P}$ = 23.8 Hz), 148.3, 147.0, 134.8 (D, $^2J_{C\text{-}P}$ = 21.1 Hz), 134.5, 129.2, 128.6, 127.4, 119.2, 64.4 (D, $^2J_{C\text{-}P}$ = 5.7 Hz), 44.6, 31.6, 29.9, 28.8, 26.7, 22.6, 16.6 (D, $^3J_{C\text{-}P}$ = 6.1 Hz), 14.1. ^{31}P NMR (202 MHz, $CDCl_3$) δ (ppm): 5.6. HRMS ESI (m/z): calculated [$C_{24}H_{31}ClN_7O_3P+H^+$] 532.1987; found 532.1994.

Diethyl (2-(4-(2-fluorophenyl)-1*H*-1,2,3-triazol-1-yl)-9-heptyl-9*H*-purin-6-yl)phosphonate (4h)

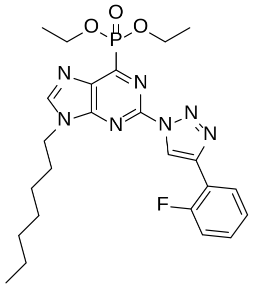

Obtained according to the general procedure of the S_NAr–Arbuzov reaction: 2,6-bis(4-(2-fluorophenyl)-1*H*-triazol-1-yl)-9-heptyl-9*H*-purine (**6h**, 120 mg, 0.22 mmol, 1.0 equiv) and $P(OEt)_3$ (2 mL). Reaction time: 8 h. Crystallization from hexane gave the product **4h** (31 mg, 27%) as an orange powder. IR (KBr) ṽ (cm^{-1}): 2958, 2926, 1620, 1468, 1236, 1010. ^1H NMR (500 MHz, $CDCl_3$) δ (ppm): 9.01 (d, 1H, $^5J_{H\text{-}F}$ = 3.6 Hz, H-C (triazole)), 8.40 (dt, 1H, $^3J_{H\text{-}H}$ = 7.6 Hz, $^4J_{H\text{-}F}$ = 1.7 Hz H-C(Ar)), 8.30 (s, 1H, H-C(8)), 7.41–7.32 (m, 1H, H-C(Ar)), 7.29 (td, 1H, $^3J_{H\text{-}H}$ = 7.6 Hz, $^4J_{H\text{-}H}$ = 1.0 Hz, H-C(Ar)), 7.19 (ddd, 1H, $^3J_{H\text{-}F}$ = 11.1 Hz, $^3J_{H\text{-}H}$ = 8.2 Hz, $^4J_{H\text{-}H}$ = 0.7 Hz, H-C(Ar)), 4.51 (quintet, 4H, ^3J = 7.1 Hz, 2×(-H_2C-O-P)), 4.41 (t, 2H, ^3J = 7.2 Hz, H_2-C(1’)), 1.97 (quintet, 2H, ^3J = 7.2 Hz H_2-C(2’)), 1.46 (t, 6H, ^3J = 7.1 Hz, 2×(-CH_3)), 1.40–1.19 (m, 8H, 4×(-CH_2-)), 0.85 (t, 3H, ^3J = 6.9 Hz, -CH_3(7’)).^{13}C NMR (125.7 MHz, $CDCl_3$) δ (ppm): 159.6 (D, $^1J_{C\text{-}F}$ = 248.9 Hz), 154.0 (D, $^3J_{C\text{-}P}$ = 11.0 Hz), 152.1 (D, $^1J_{C\text{-}P}$ = 221.2 Hz), 148.9 (D, $^3J_{C\text{-}P}$ = 23.5 Hz), 148.2, 141.7 (D, $^3J_{C\text{-}F}$ = 2.2 Hz), 135.0 (D, $^2J_{C\text{-}P}$ = 21.1 Hz), 130.0 (D, $^3J_{C\text{-}F}$ = 8.3 Hz), 128.3 (D, $^3J_{C\text{-}F}$ = 3.2 Hz), 124.8 (D, $^3J_{C\text{-}F}$ = 3.2 Hz), 122.1 (D, $^4J_{C\text{-}F}$ = 13.4 Hz), 118.2 (D, $^2J_{C\text{-}F}$ = 13.0 Hz), 115.9 (D, $^2J_{C\text{-}F}$ = 21.7 Hz), 64.6 (D, $^2J_{C\text{-}P}$ = 6.1 Hz), 44.6, 31.7, 29.9, 28.8, 26.7, 22.6, 16.6 (D, $^3J_{C\text{-}P}$ = 6.2 Hz), 14.1. ^{31}P NMR (202 MHz, $CDCl_3$) δ (ppm): 5.6. HRMS ESI (m/z): calculated [$C_{24}H_{31}FN_7O_3P+H^+$] 516.2283, found 516.2268.

Diethyl (9-heptyl-2-(4-pyrdin-2-yl)-1*H*-1,2,3-triazol-1-yl)-9*H*-purin-6-yl)phosphonate (4i)

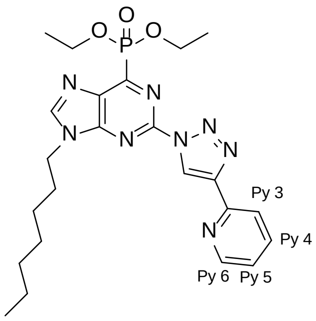

Obtained according to the general procedure of the S_NAr–Arbuzov reaction: 9-heptyl-2,6-bis(4-pyridin-2-yl)-1*H*-1,2,3-triazol-1-yl)-9*H*-purine (**6i**, 100 mg, 0.20 mmol, 1.0 equiv) and $P(OEt)_3$ (1.5 mL). Reaction time: 14 h. Crystallization from hexane gave the product **4i** (70 mg, 70%) as an orange powder. M_p = 126–128 °C. IR (KBr) ṽ (cm^{-1}): 2958, 2926, 1620, 1468, 1236, 1010. ^1H NMR (500 MHz, DMSO-d_6) δ (ppm): 9.21 (s, 1H, H-C(triazole)), 8.93 (s, 1H, H-C(8)), 8.68 (ddd, 1H, ^3J = 4.8 Hz, ^4J = 1.9 Hz, ^5J = 0.9 Hz, H-C(Py(3))), 8.18 (dt, 1H, ^3J = 7.7 Hz, ^4J = ^5J = 0.9 Hz, H-C(Py(6))), 7.97 (td, 1H, ^3J = 7.7 Hz, ^4J =1.9 Hz, H-C(Py(5))), 7.43 (ddd, 1H, ^3J = 7.7 Hz, 4.8 Hz, ^4J = 0.9 Hz, H-C(Py(4))), 4.50–4.26 (m, 6H, H_2-C(1’), 2×(-CH_2-O-P)), 1.93 (quintet, 2H ^3J= 6.9 Hz, H_2-C(2’)),

1.37 (t, 6H, ^3J = 7.0 Hz, 2×(-CH_3)), 1.34–1.19 (m, 8H, 4×(-CH_2-)), 0.82 (t, 3H, ^3J = 6.9 Hz, H_3-C(7')). ^{13}C NMR (125.7 MHz, DMSO-d_6) δ (ppm): 153.7 (D, $^3J_{C-P}$ = 11.3 Hz), 150.6 (D, $^1J_{C-P}$ = 217.9 Hz), 150.4, 149.9, 149.0, 147.7 (D, $^3J_{C-P}$= 23.9 Hz), 147.68, 137.4, 134.6 (D, $^2J_{C-P}$ = 21.8 Hz), 123.6, 121.5, 120.0, 63.7 (D, $^2J_{C-P}$ = 6.0 Hz), 43.6, 31.1, 28.9, 28.1, 25.9, 22.0, 16.3 (D, $^3J_{C-P}$ = 6.2 Hz), 13.8. ^{31}P NMR (202 MHz, DMSO-d_6) δ (ppm): 5.5. HRMS ESI (m/z): calculated [$C_{23}H_{31}N_8O_3P+H^+$] 499.2329, found 499.2299.

Copies of ^{1}H, ^{13}C, and ^{31}P NMR spectra

Diethyl (2-chloro-9-heptyl-9*H*-purin-6-yl)phosphonate (2a)

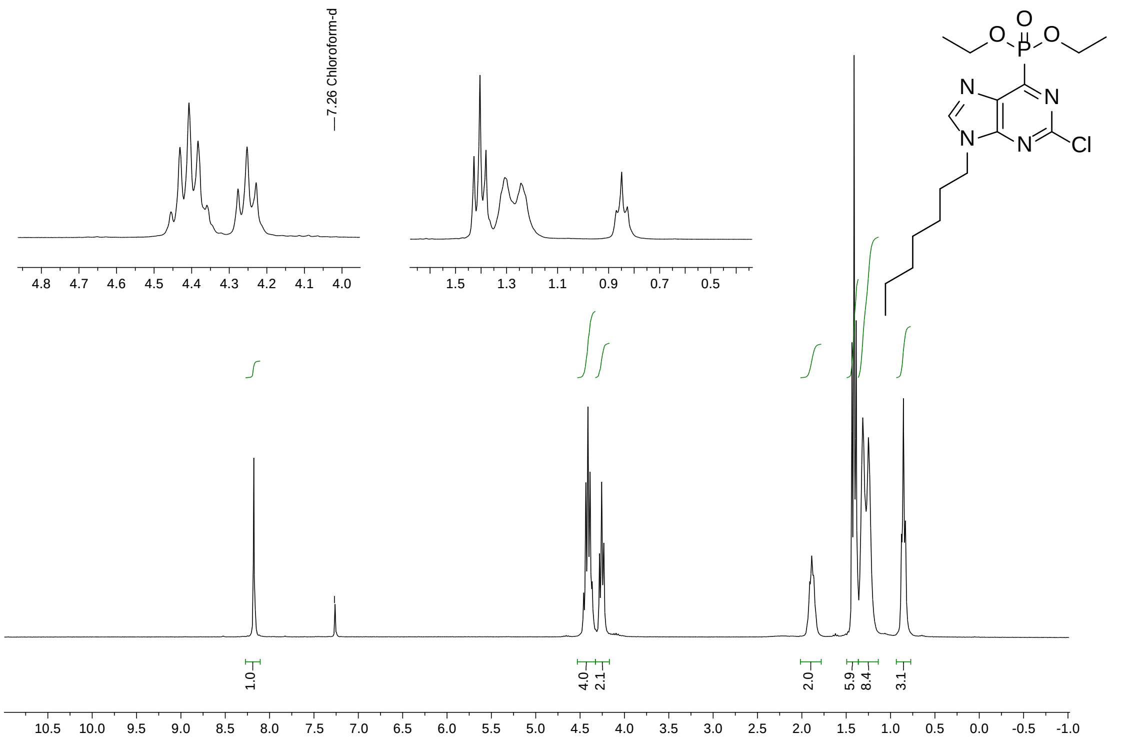

Figure S5: ^{1}H NMR (300 MHz, $CDCl_3$) spectrum.

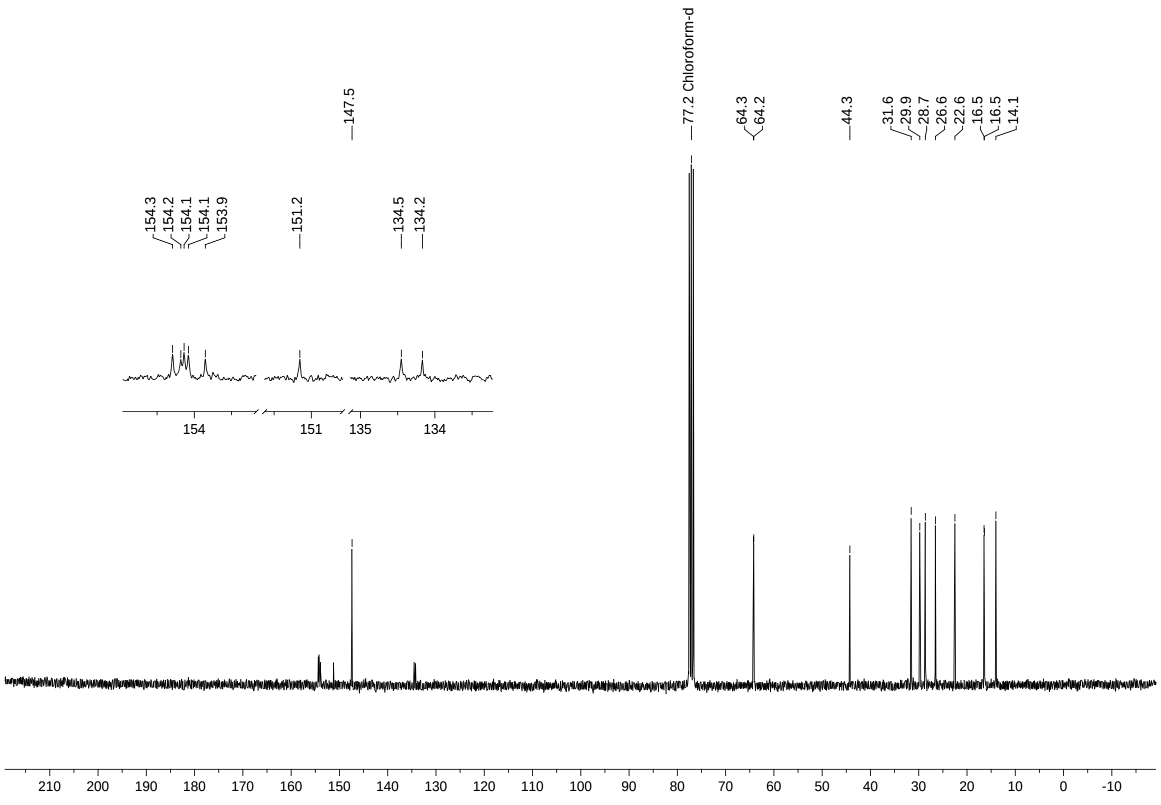

Figure S6: ^{13}C NMR (75.5 MHz, $CDCl_3$) spectrum.

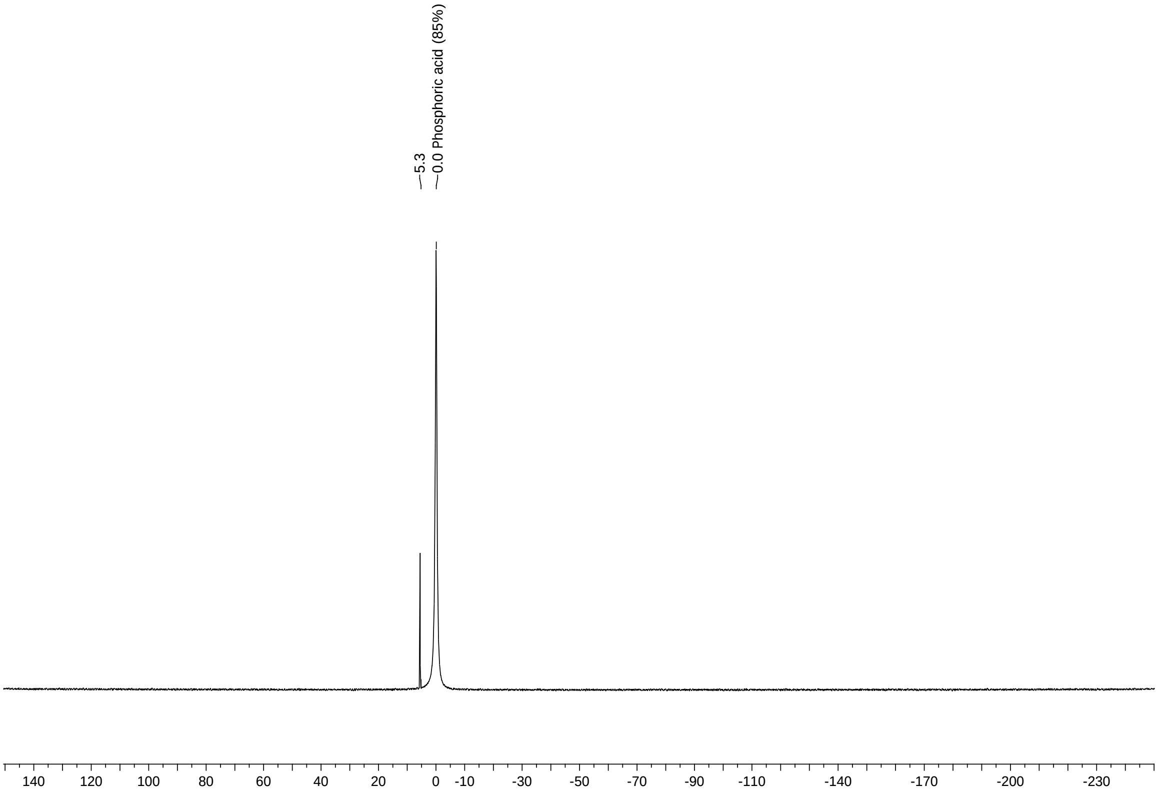

Figure S7: ^{31}P NMR (121 MHz, $CDCl_3$) spectrum.

Diisopropyl (2-chloro-9-heptyl-9*H*-purin-6-yl)phosphonate (2b)

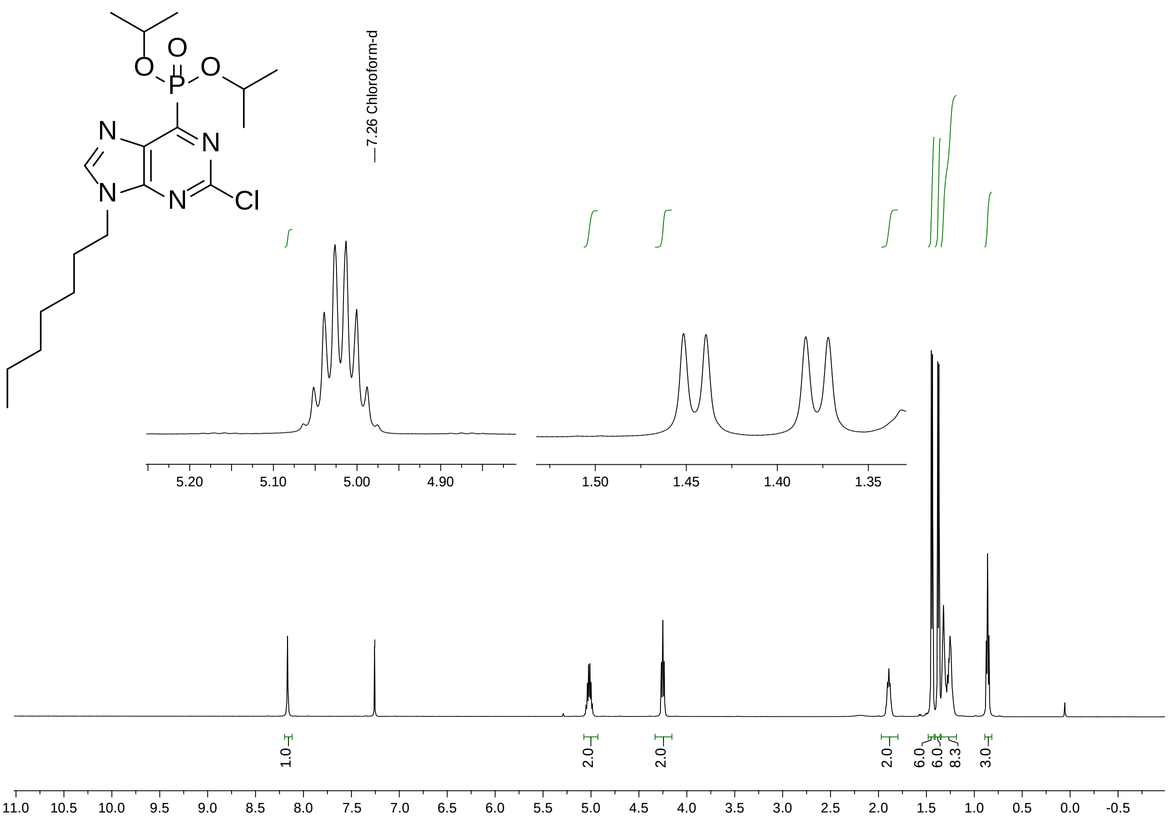

Figure S8: ^{1}H NMR (500 MHz, $CDCl_3$) spectrum.

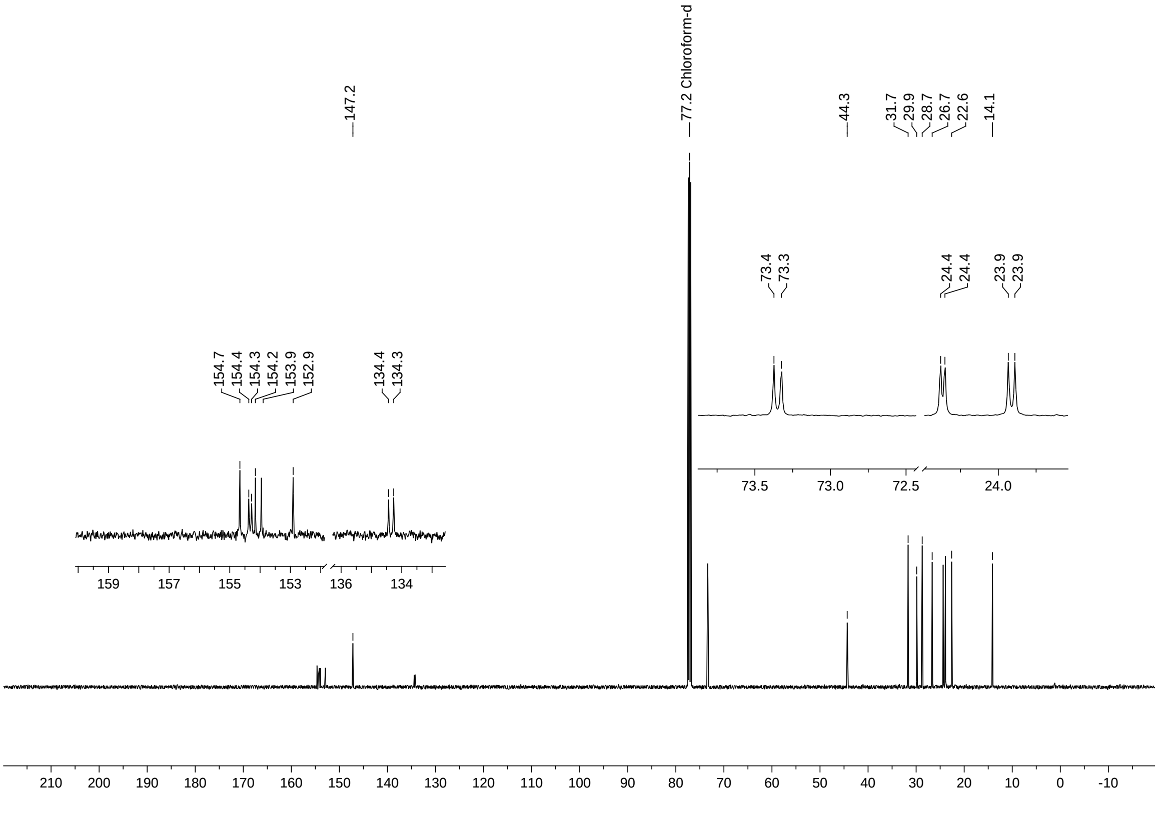

Figure S9: ^{13}C NMR (125.7 MHz, $CDCl_3$) spectrum.

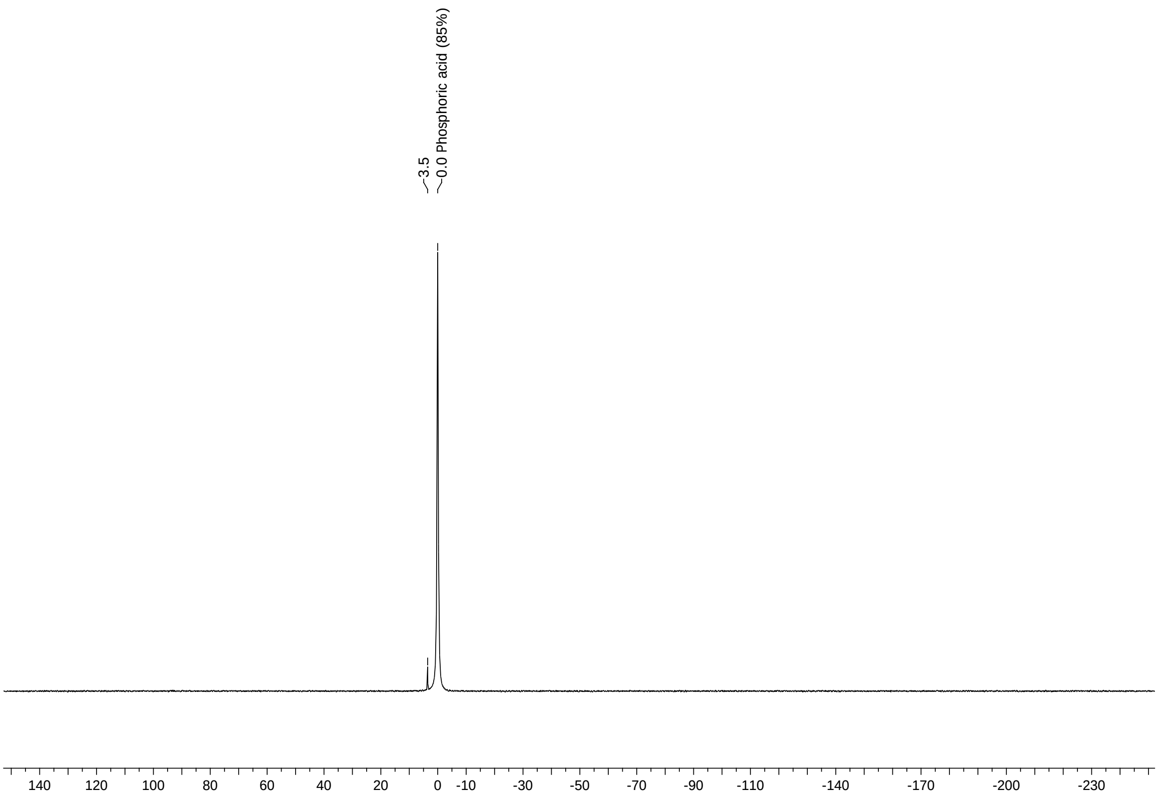

Figure S10: ^{31}P NMR (121 MHz, $CDCl_3$) spectrum.

Monoethyl (2-azido-9-heptyl-9*H*-purin-6-yl)phosphonate (9a)

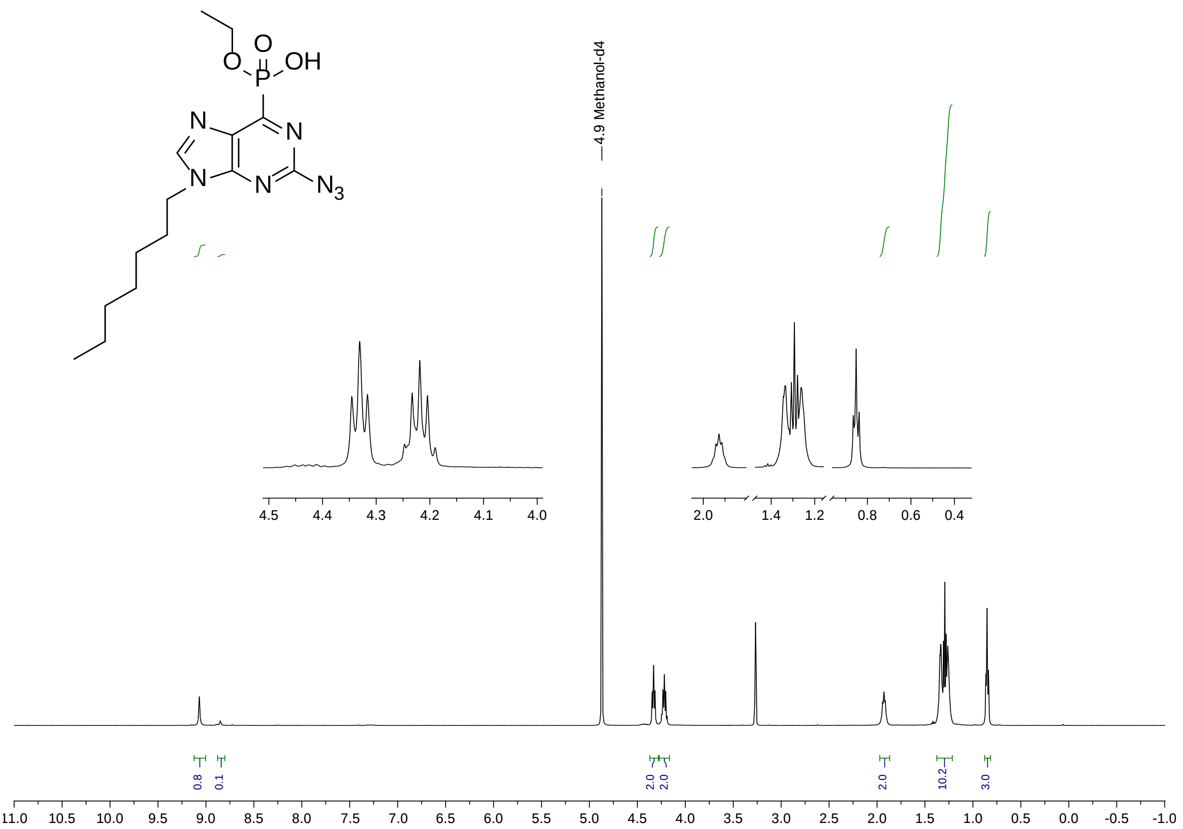

Figure S11: ^{1}H NMR (500 MHz, MeOD-d$_4$) spectrum.

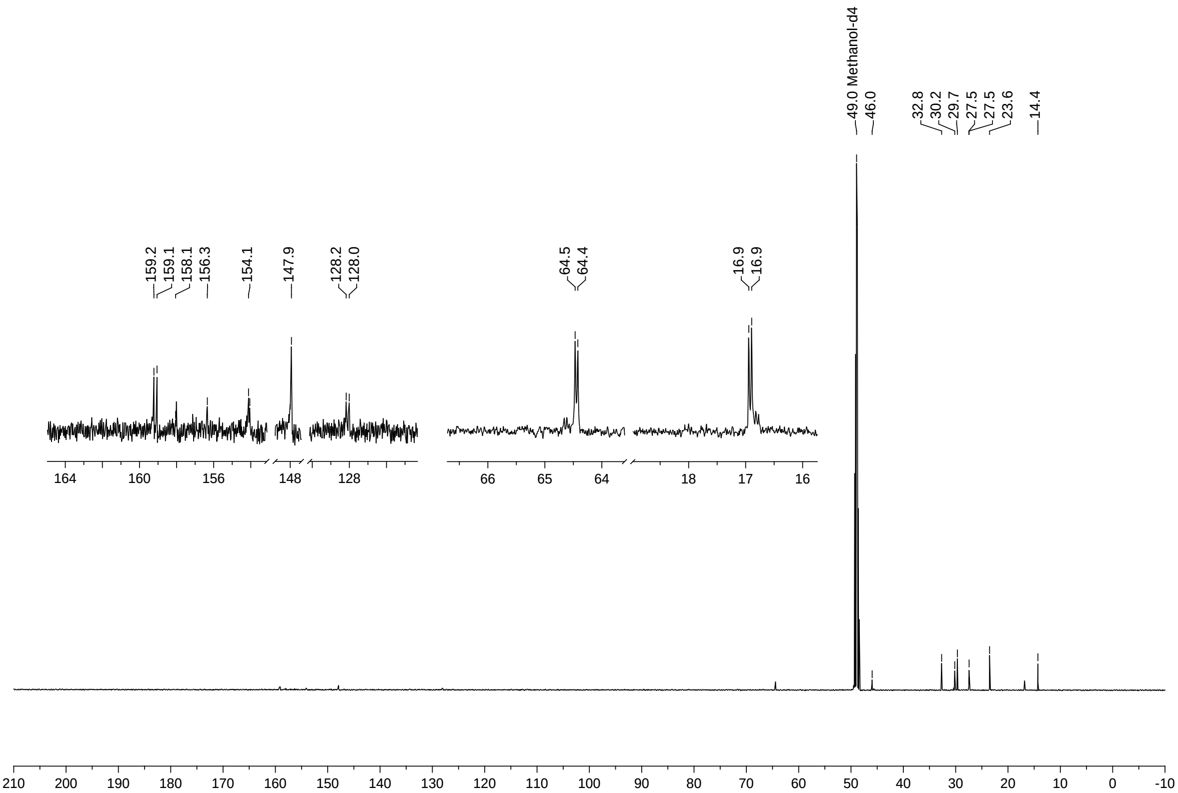

Figure S12: ^{13}C NMR (125.7 MHz, MeOD-d$_4$) spectrum.

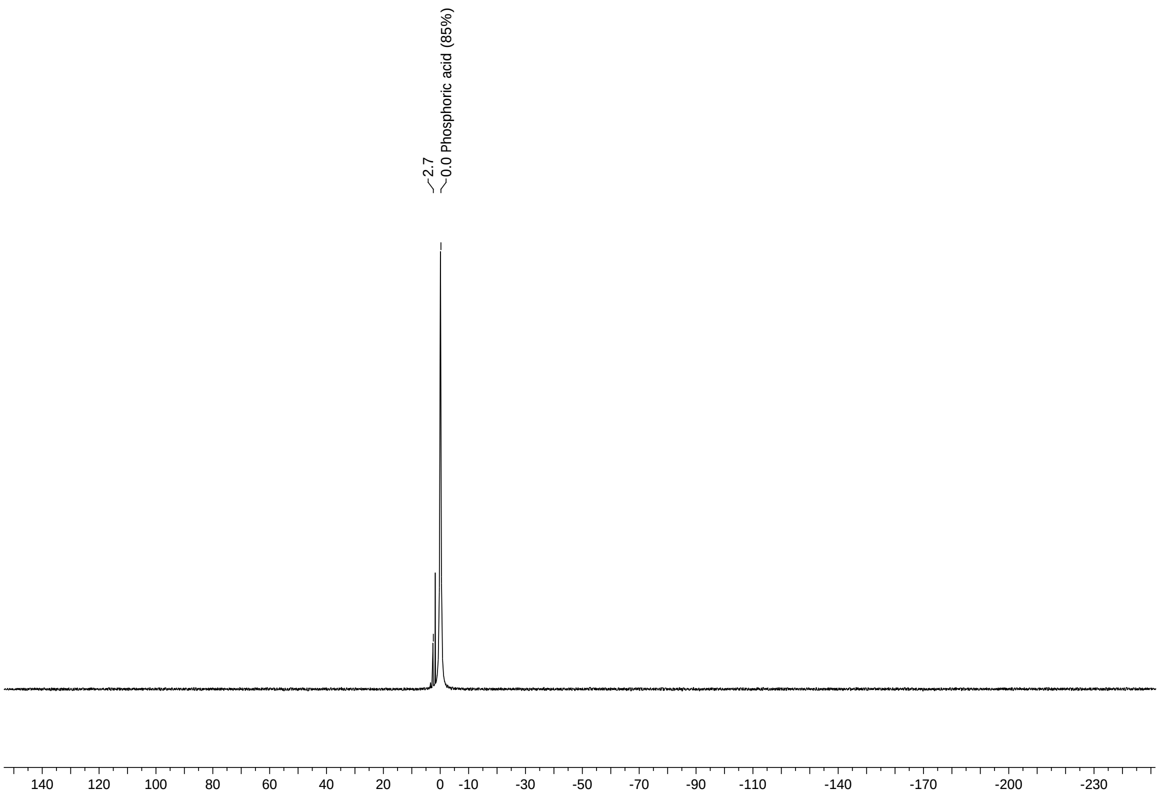

Figure S13: 3^{1}P NMR (202 MHz, MeOD-d_4) spectrum.

Monoisopropyl (2-azido-9-heptyl-9*H*-purin-6-yl)phosphonate (9b)

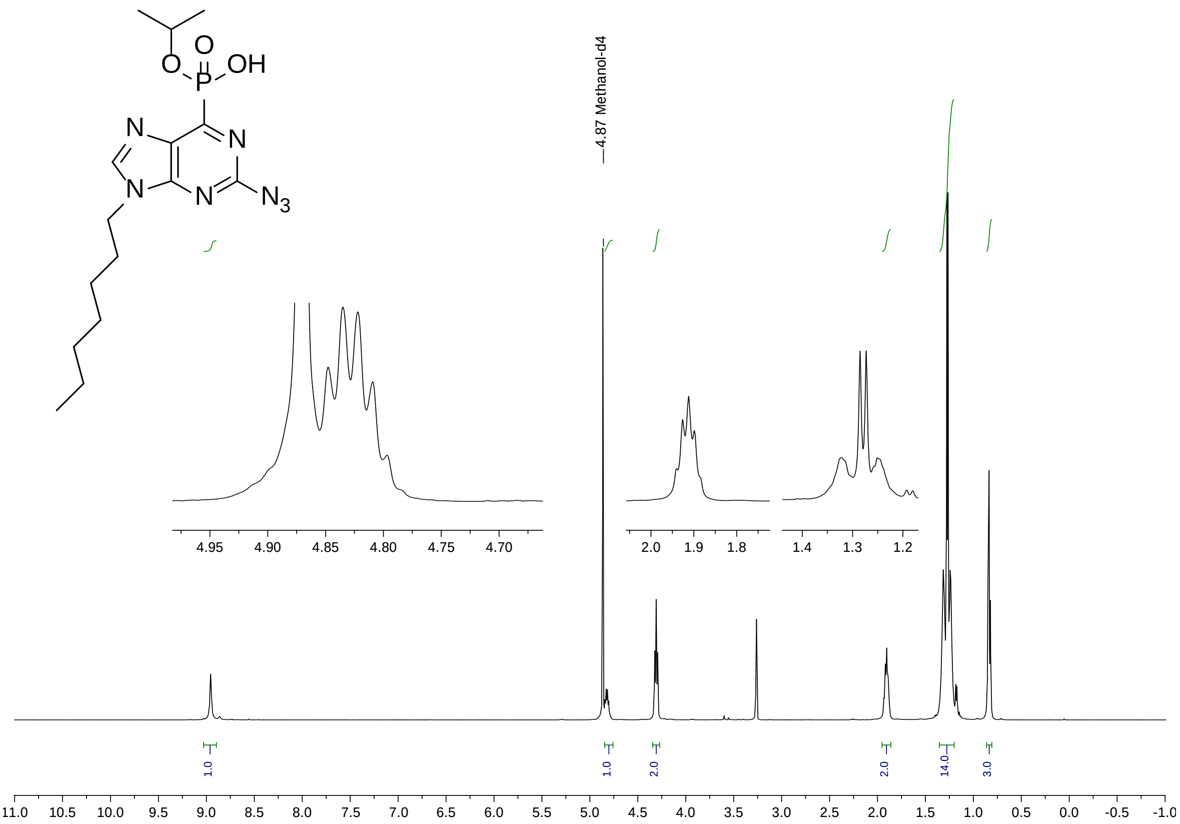

Figure S14: ^{1}H NMR (500 MHz, MeOD-d$_4$) spectrum.

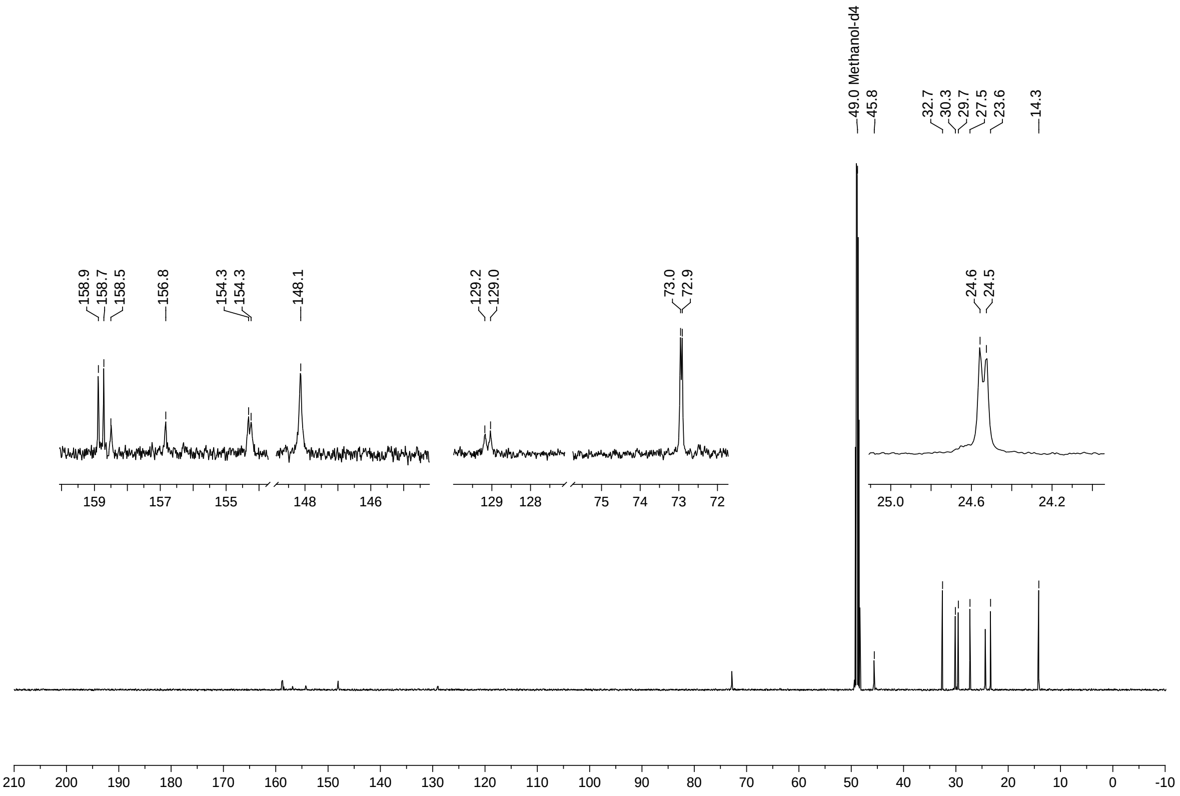

Figure S15: ^{13}C NMR (125.7 MHz, MeOD-d$_4$) spectrum.

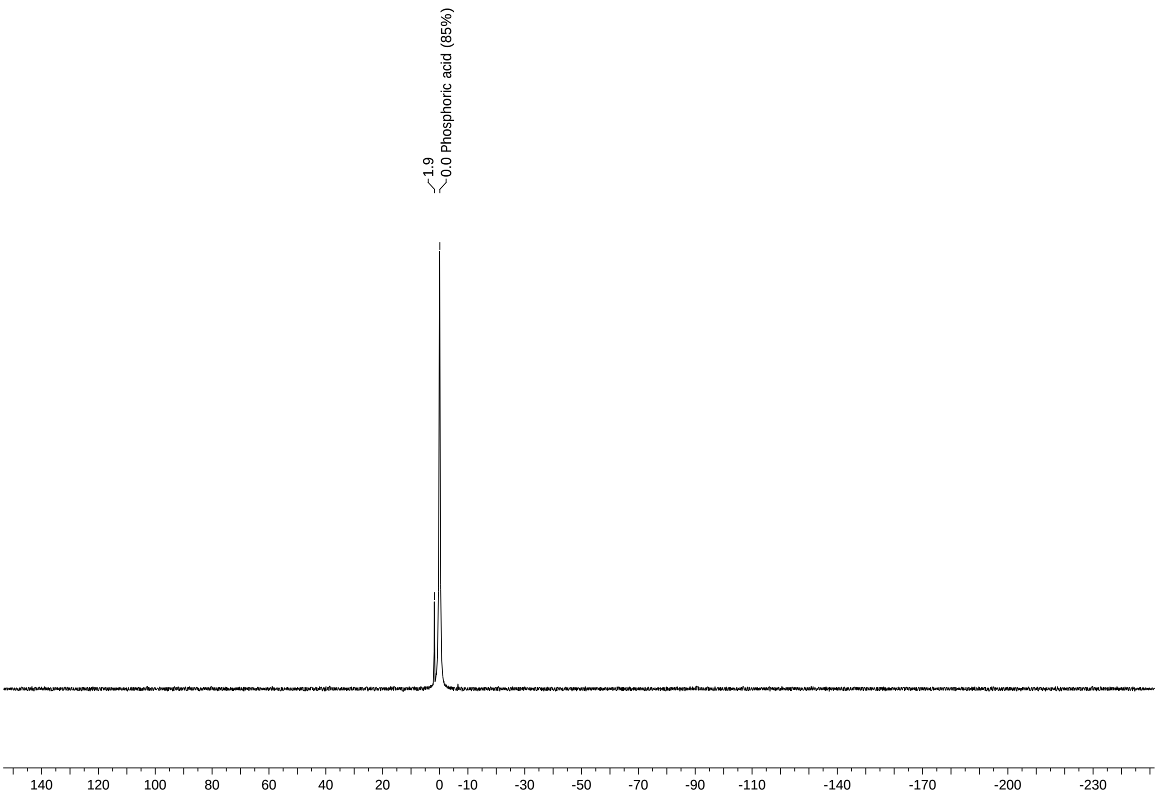

Figure S16: ^{31}P NMR (202 MHz, MeOD-d_4) spectrum.

(2-Chloro-9-heptyl-9*H*-purin-6-yl)phosphonic acid (10)

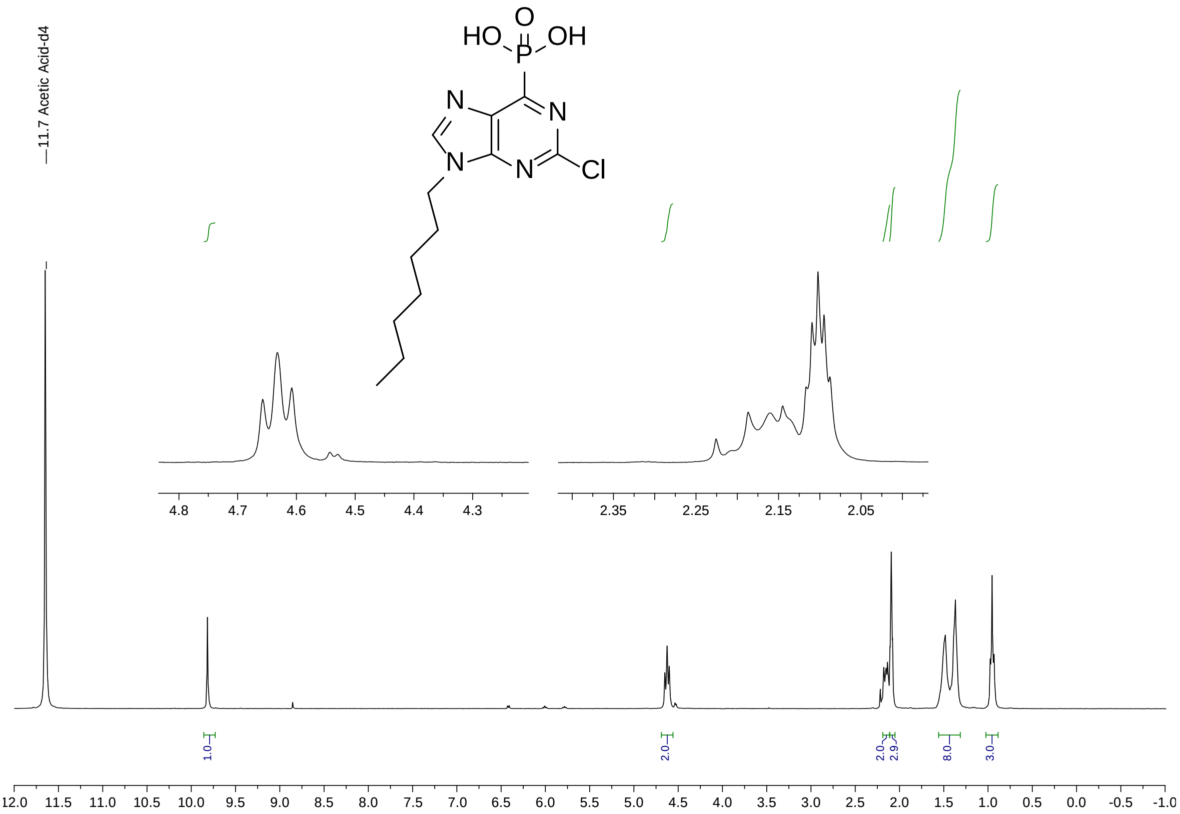

Figure S17: ^{1}H NMR (300 MHz, AcOD-d$_4$) spectrum.

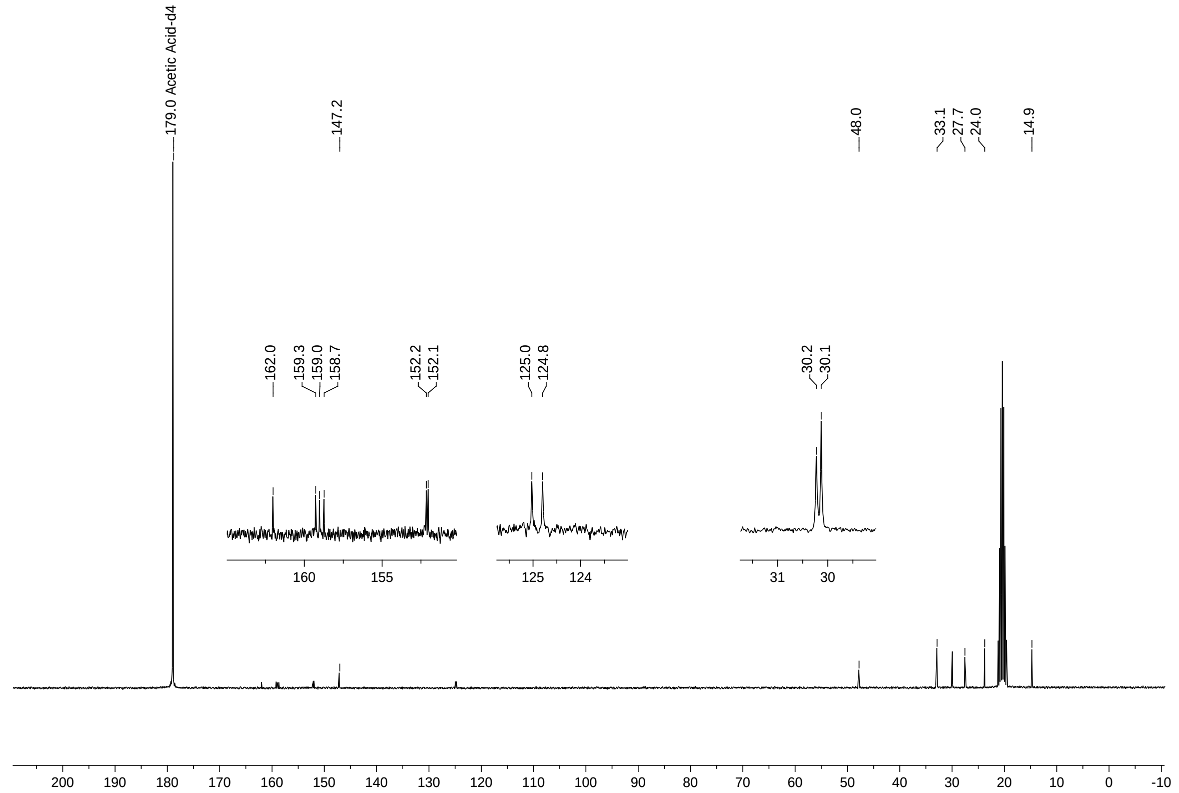

Figure S18: ^{13}C NMR (75.5 MHz, AcOD-d$_4$) spectrum.

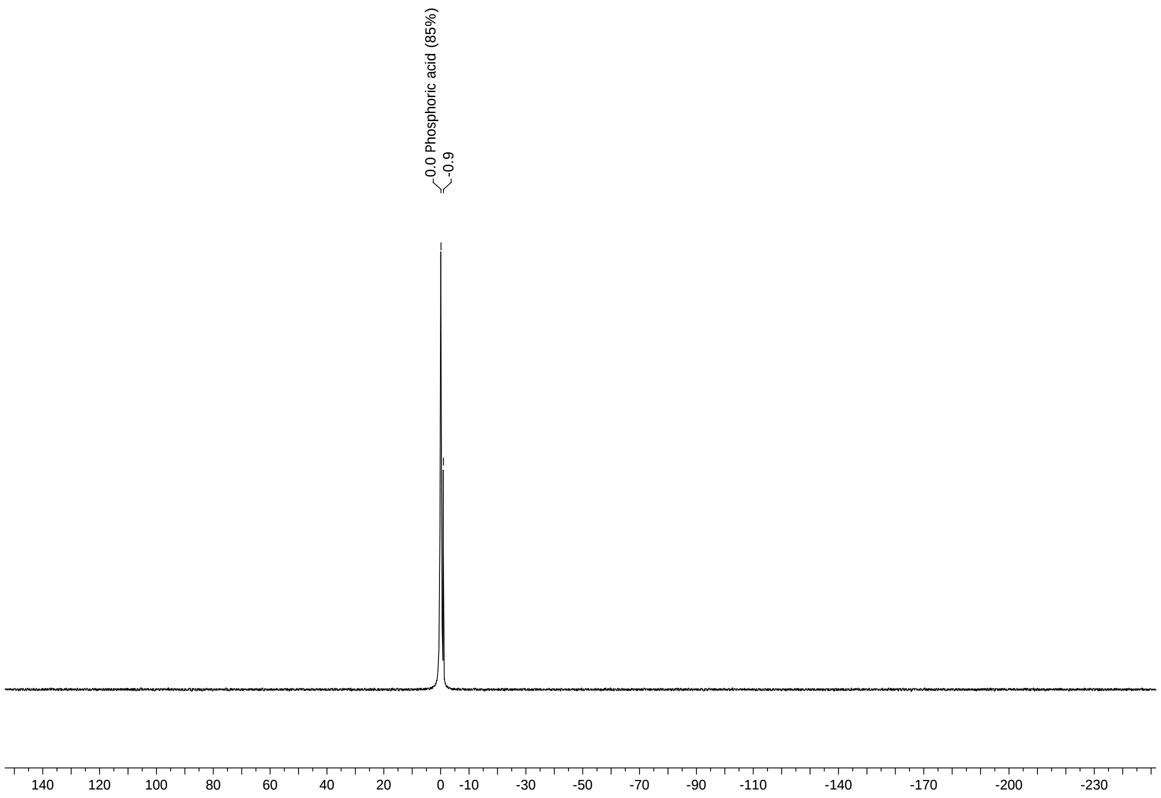

Figure S19: ^{31}P NMR (202 MHz, AcOD-d_4) spectrum.

Dimethyl 1,1'-(9-heptyl-9*H*-purine-2,6-diyl)bis(1*H*-1,2,3-triazole-4-carboxylate) (6a)

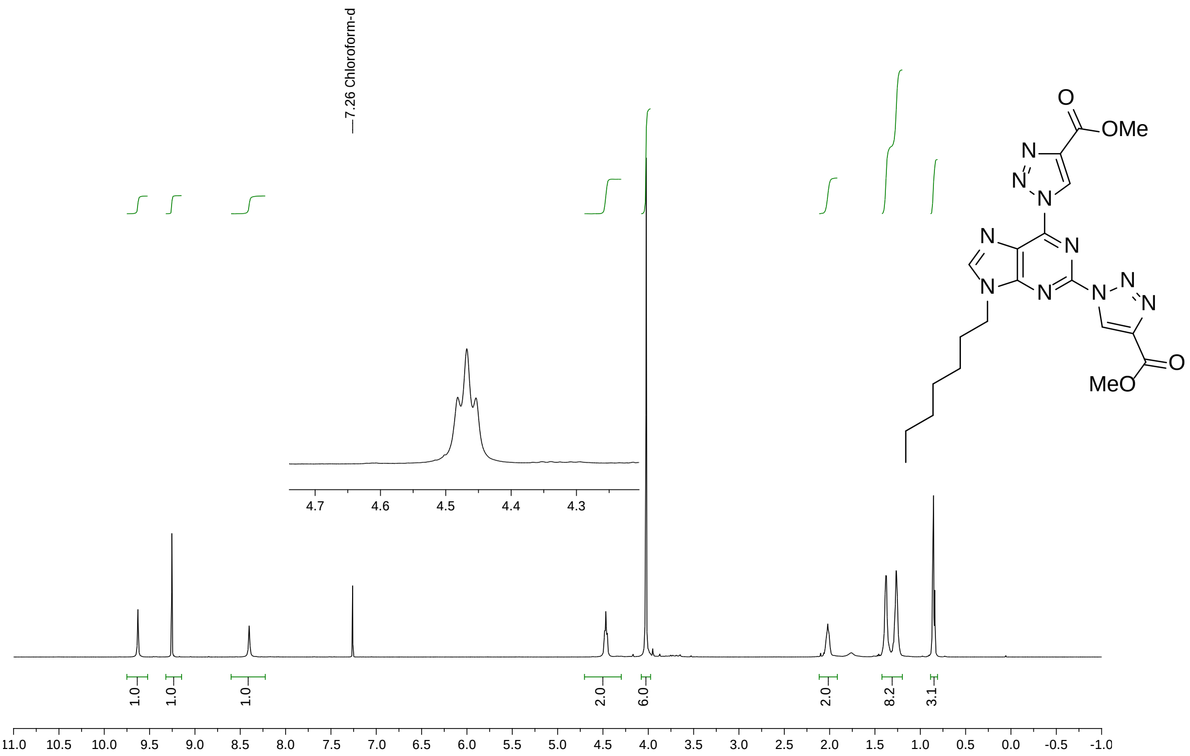

Figure S20: ^{1}H NMR (500 MHz, $CDCl_3$) spectrum.

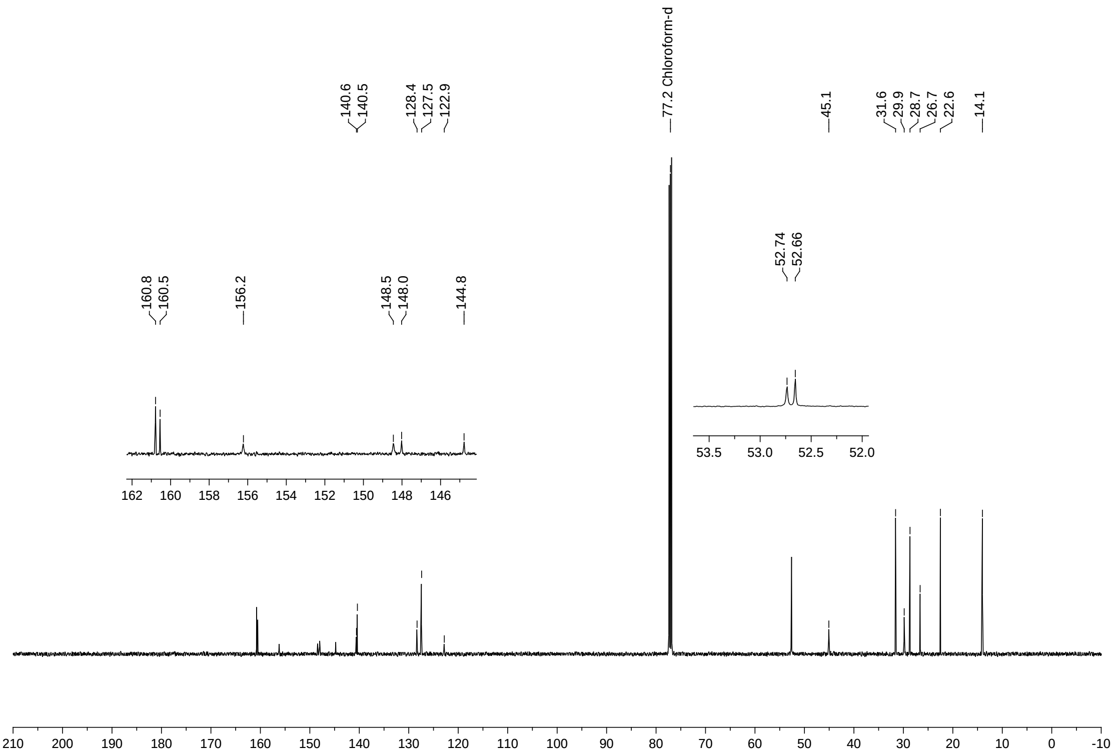

Figure S21: ^{13}C NMR (125.7 MHz, $CDCl_3$) spectrum.

2,6-Bis(4-(3-cyanopropyl)-1*H*-1,2,3-triazol-1-yl)-9-heptyl-9*H*-purine (6b)

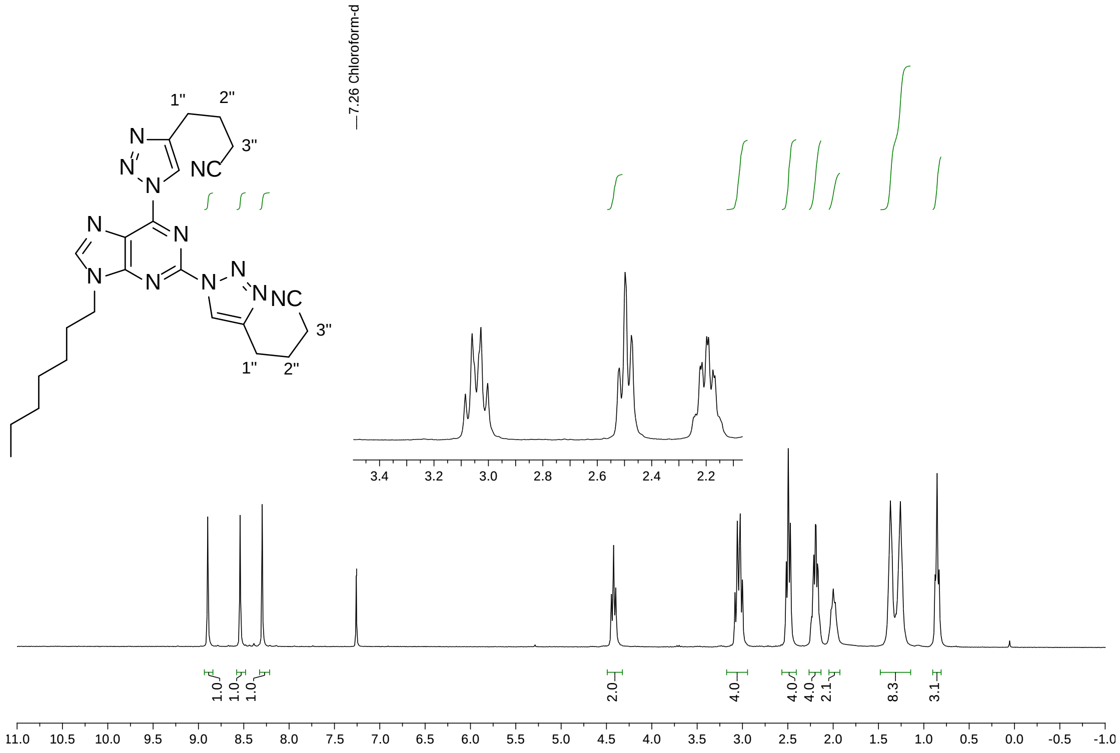

Figure S22: ^{1}H NMR (300 MHz, $CDCl_3$) spectrum.

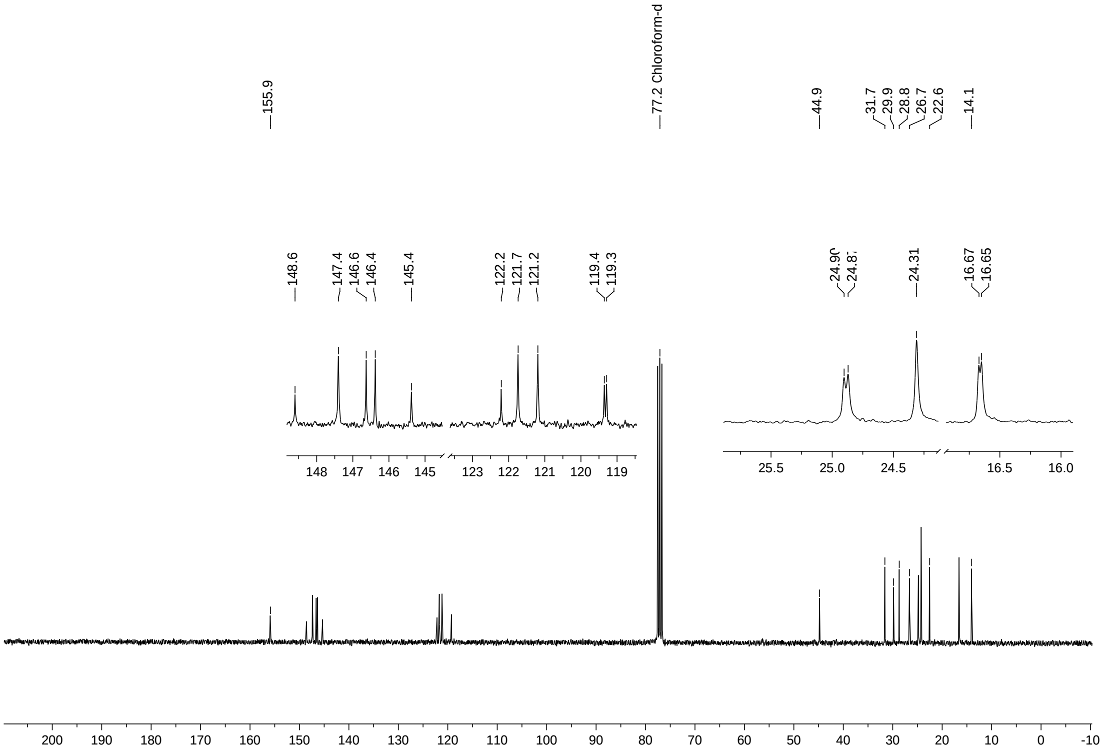

Figure S23: ^{13}C NMR (75.5 MHz, $CDCl_3$) spectrum.

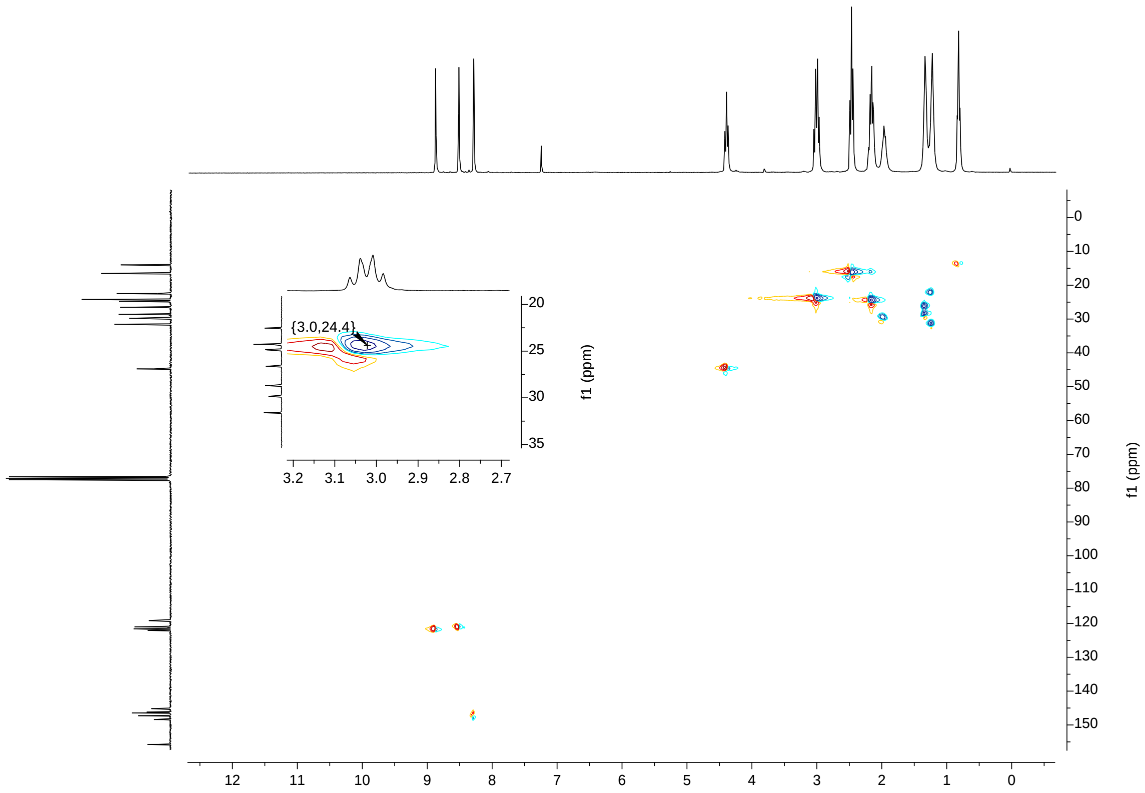

Figure S24: ^{1}H-^{13}C HSQC spectrum of **6b**.

2,6-Bis(4-butyl-1*H*-1,2,3-triazol-1-yl)-9-heptyl-9*H*-purine (6c)

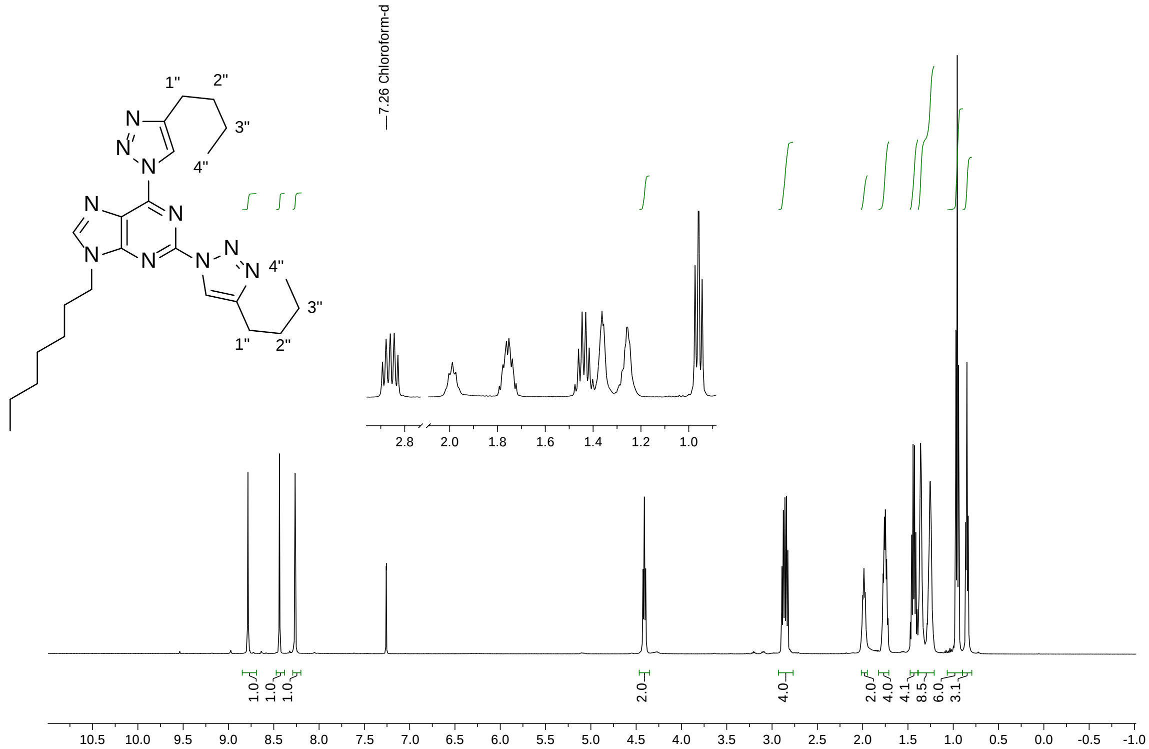

Figure S25: ^{1}H NMR (500 MHz, $CDCl_3$) spectrum.

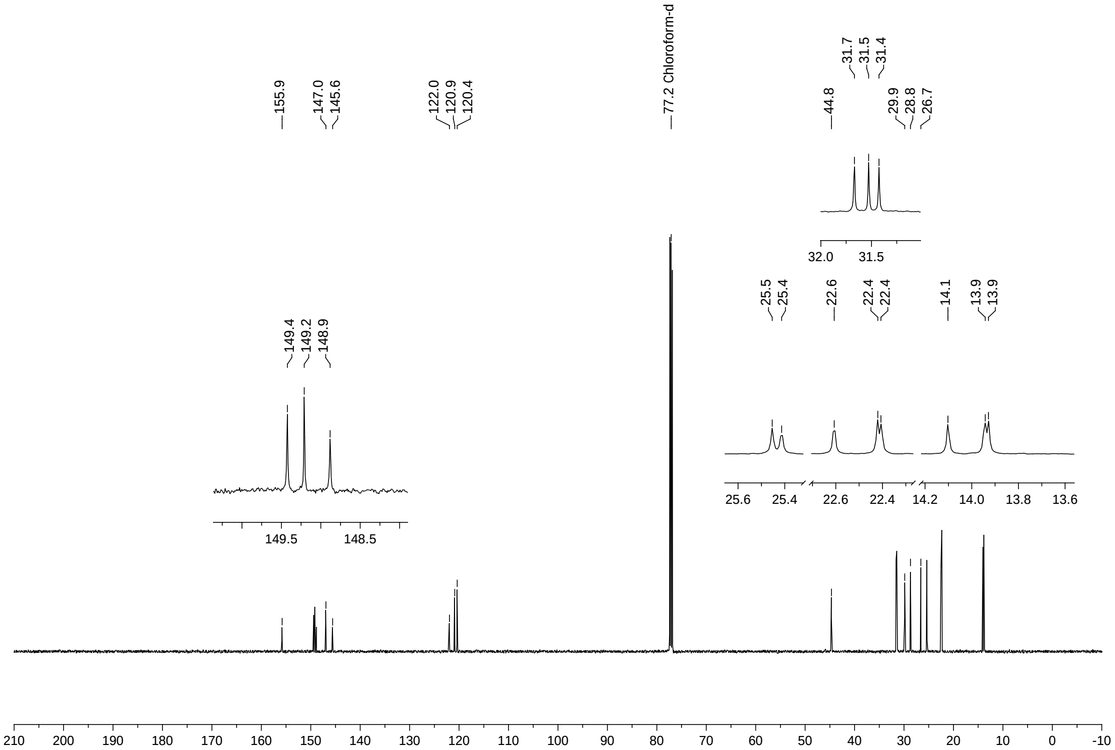

Figure S26: ^{13}C NMR (125.7 MHz, $CDCl_3$) spectrum.

2,6-Bis(4-(4-cyanophenyl)-1*H*-1,2,3-triazol-1-yl)-9-heptyl-9*H*-purine (6e)

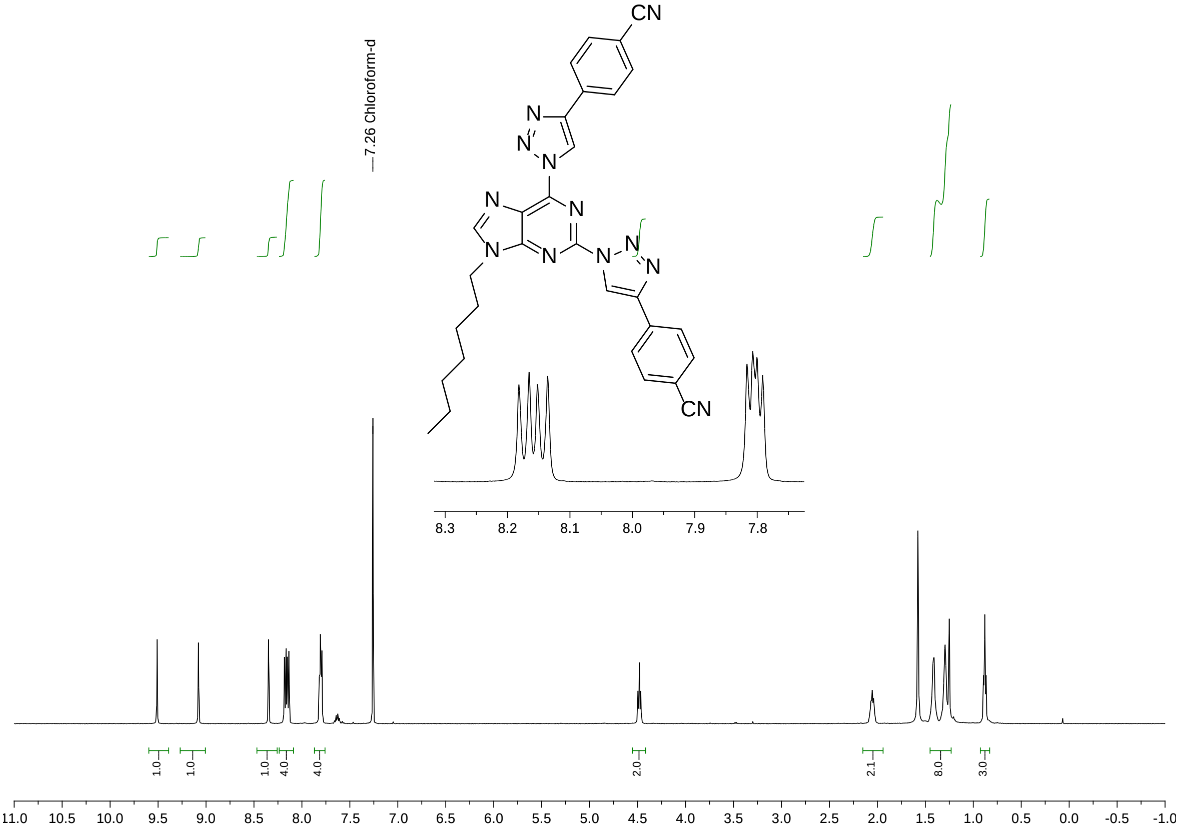

Figure S27: ^{1}H NMR (500 MHz, $CDCl_3$) spectrum.

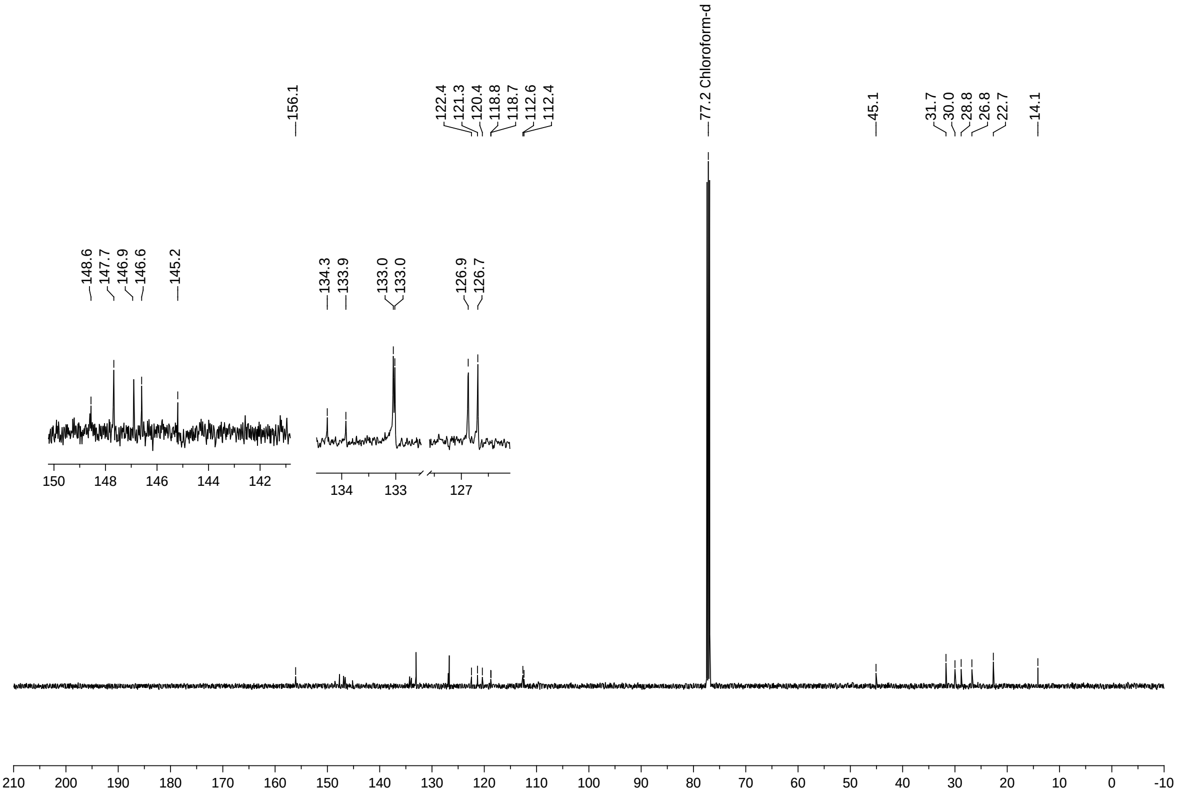

Figure S28: ^{13}C NMR (125.7 MHz, $CDCl_3$) spectrum

9-Heptyl-2,6-bis(4-(4-methoxyphenyl)-1*H*-1,2,3-triazol-1-yl)-9*H*-purine (6f)

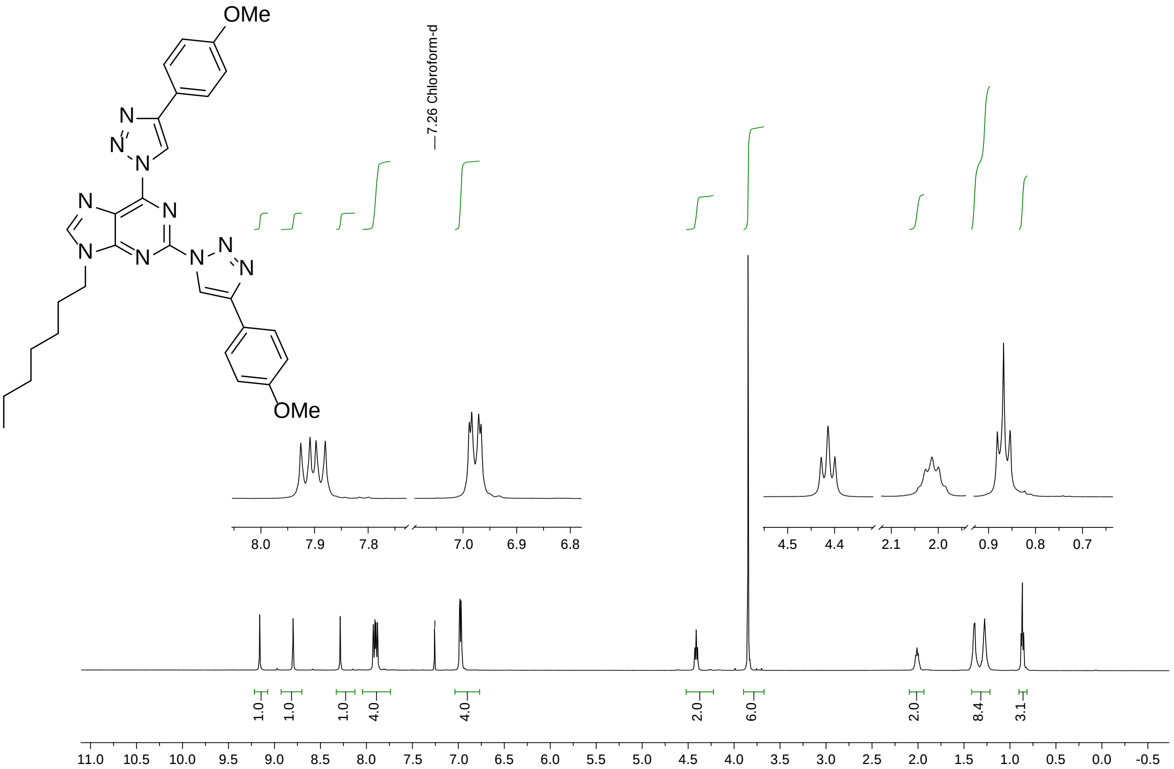

Figure S29: ^{1}H NMR (500 MHz, $CDCl_3$) spectrum.

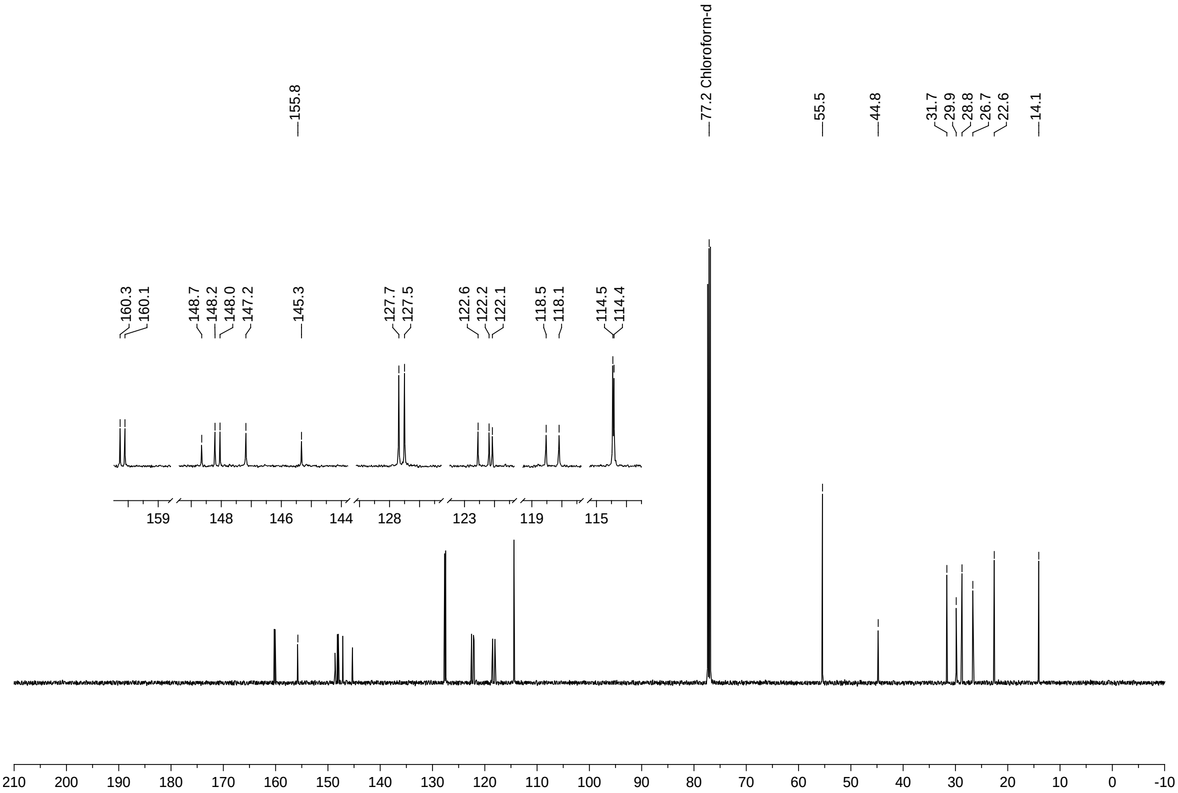

Figure S30: ^{13}C NMR (125.7 MHz, $CDCl_3$) spectrum.

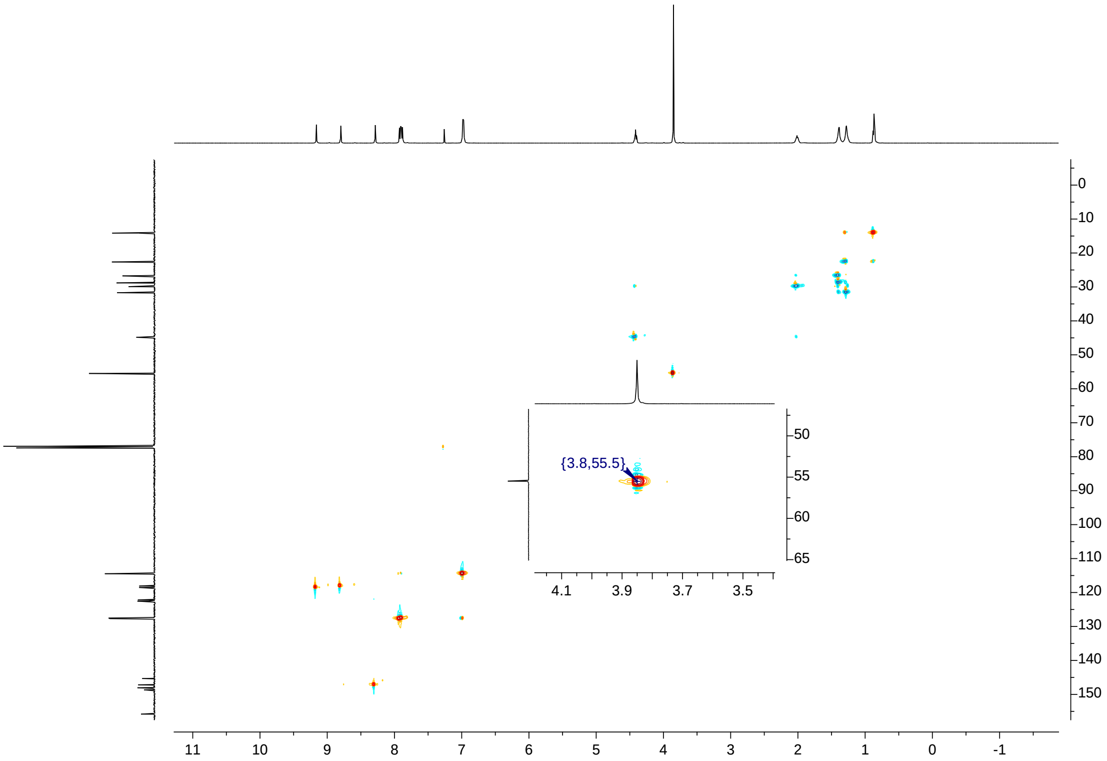

Figure S31: ^{1}H-^{13}C HSQC spectrum of compound **6f**.

2,6-Bis(4-(4-chlorophenyl)-1*H*-1,2,3-triazol-1-yl)-9-heptyl-9*H*-purine (6g)

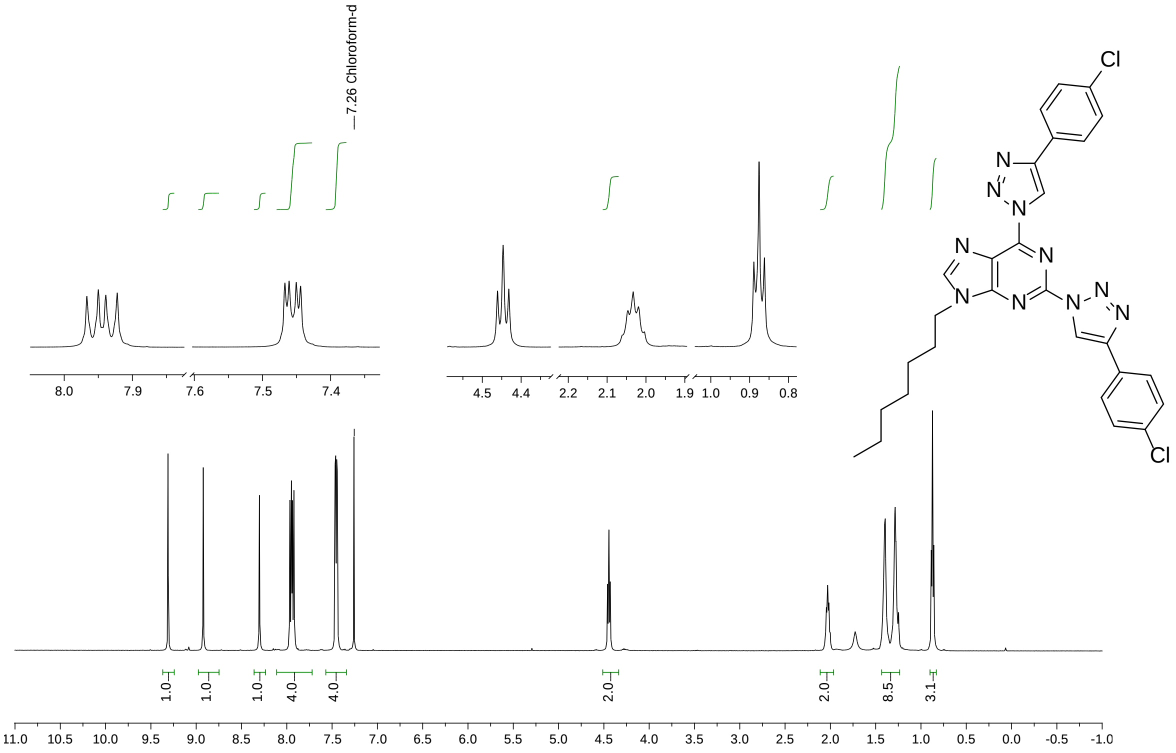

Figure S32: ^{1}H NMR (500 MHz, $CDCl_3$) spectrum.

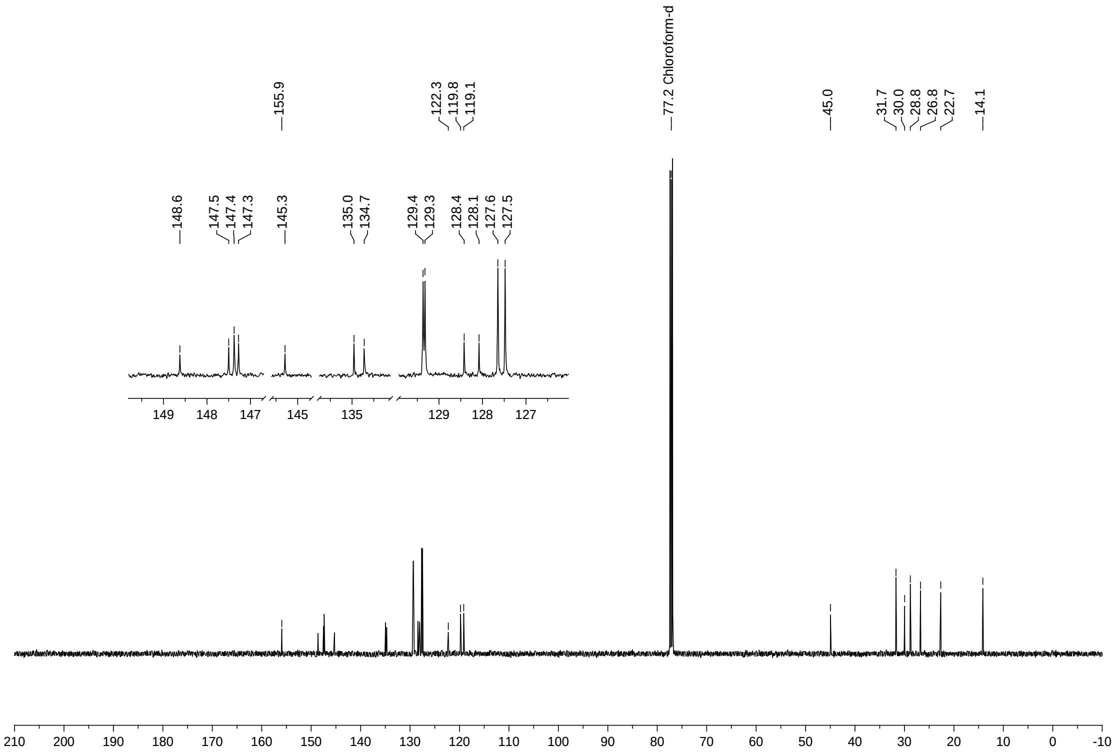

Figure S33: ^{13}C NMR (125.7 MHz, $CDCl_3$) spectrum.

2,6-Bis(4-(2-fluorophenyl)-1*H*-1,2,3-triazol-1-yl)-9-heptyl-9*H*-purine (6h)

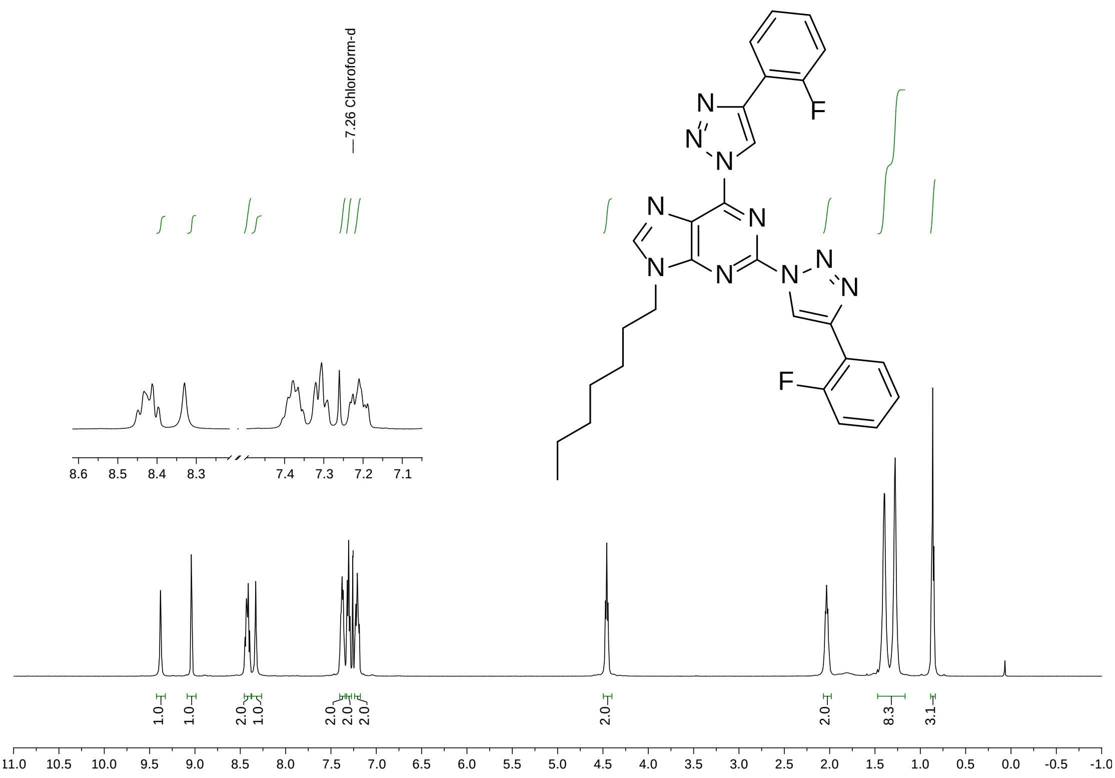

Figure S34: ^{1}H NMR (500 MHz, $CDCl_3$) spectrum.

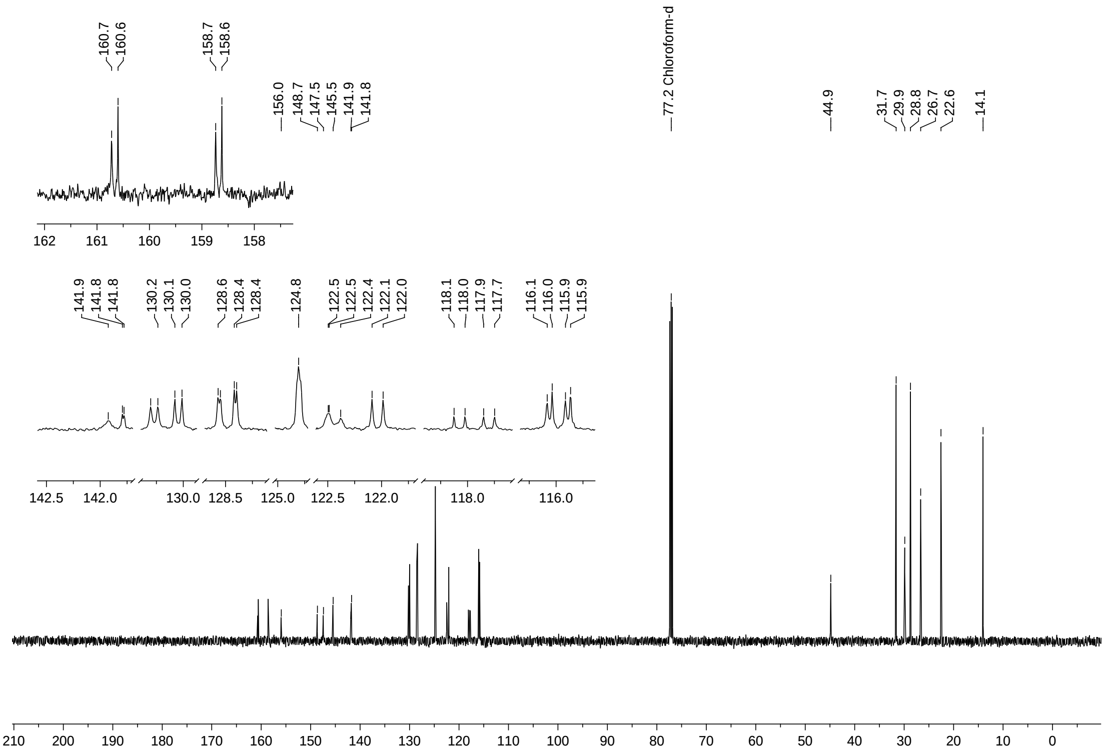

Figure S35: ^{13}C NMR (125.7 MHz, $CDCl_3$) spectrum.

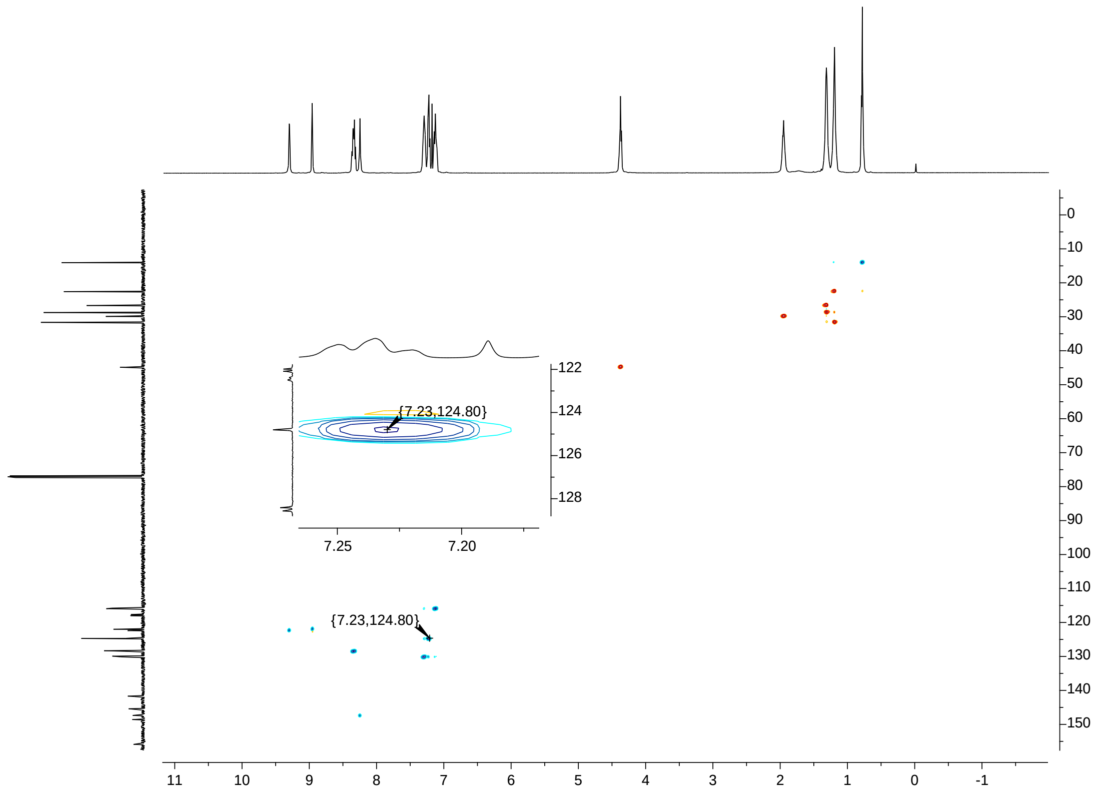

Figure S36: ^{1}H-^{13}C HSQC spectrum of compound **6h**.

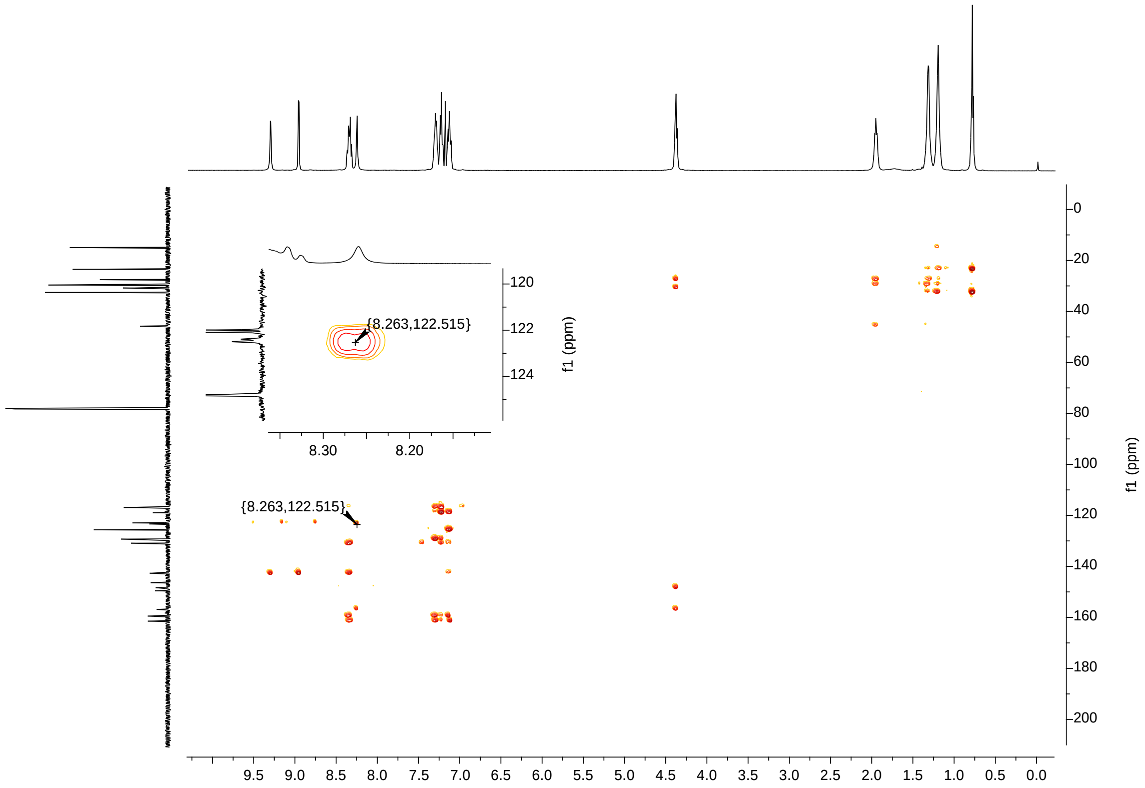

Figure S37: ^{1}H-^{13}C HMBC spectrum of compound **6h**.

9-Heptyl-2,6-bis(4-(pyridin-2-yl)-1*H*-1,2,3-triazol-1-yl)-9*H*-purine (6i)

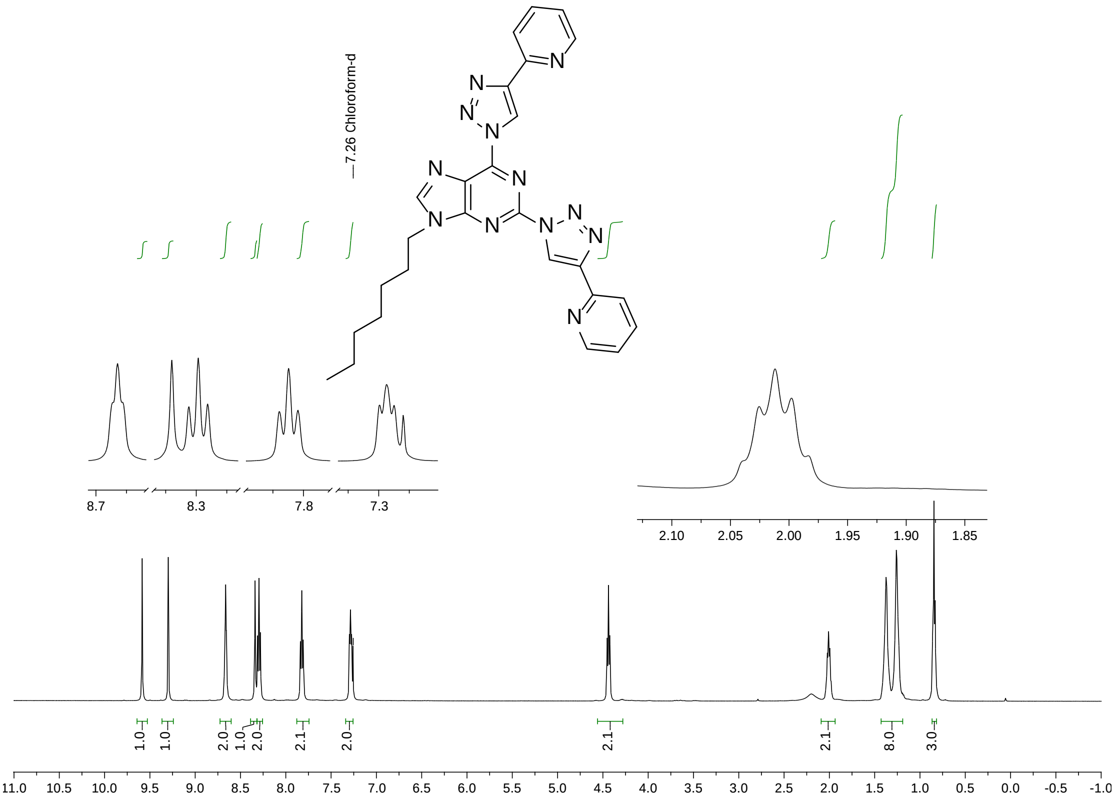

Figure S38: ^{1}H NMR (500 MHz, $CDCl_3$) spectrum.

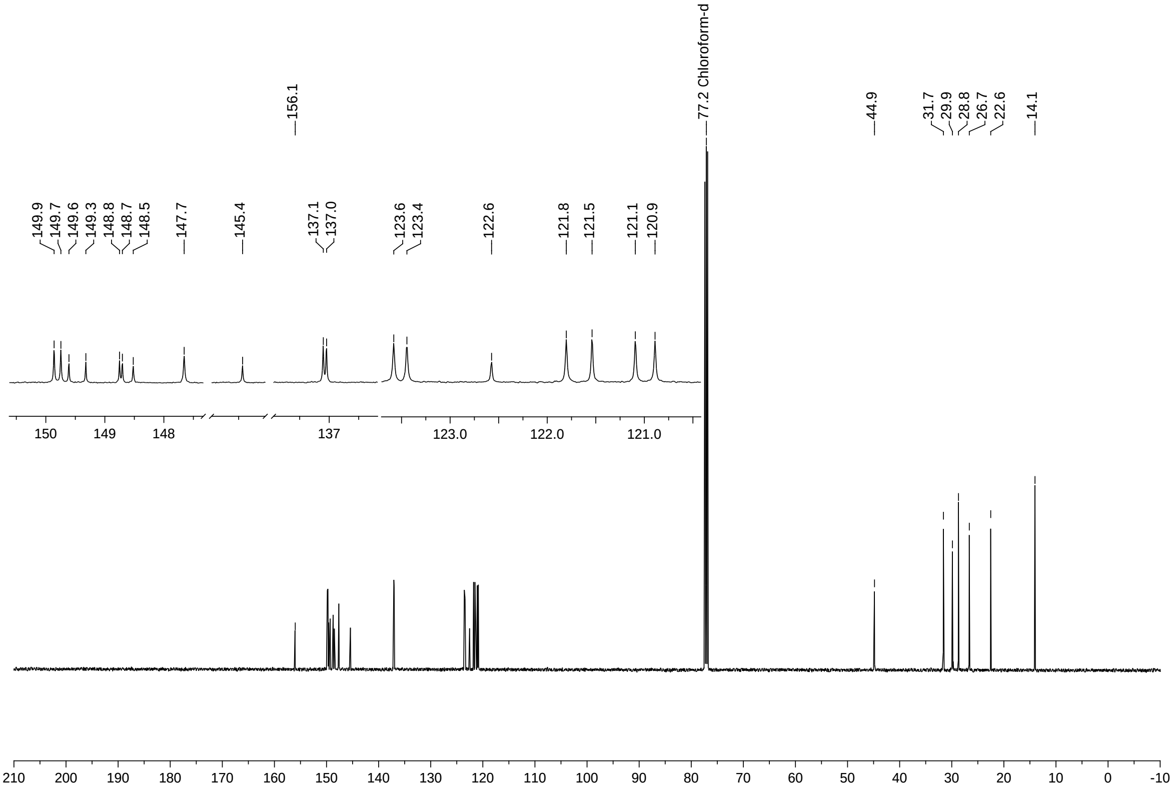

Figure S39: ^{13}C NMR (125.7 MHz, $CDCl_3$) spectrum.

Methyl 1-(6-(diethoxyphosphoryl)-9-heptyl-9*H*-purin-2-yl)-1*H*-1,2,3-triazole-4-carboxylate (4a)

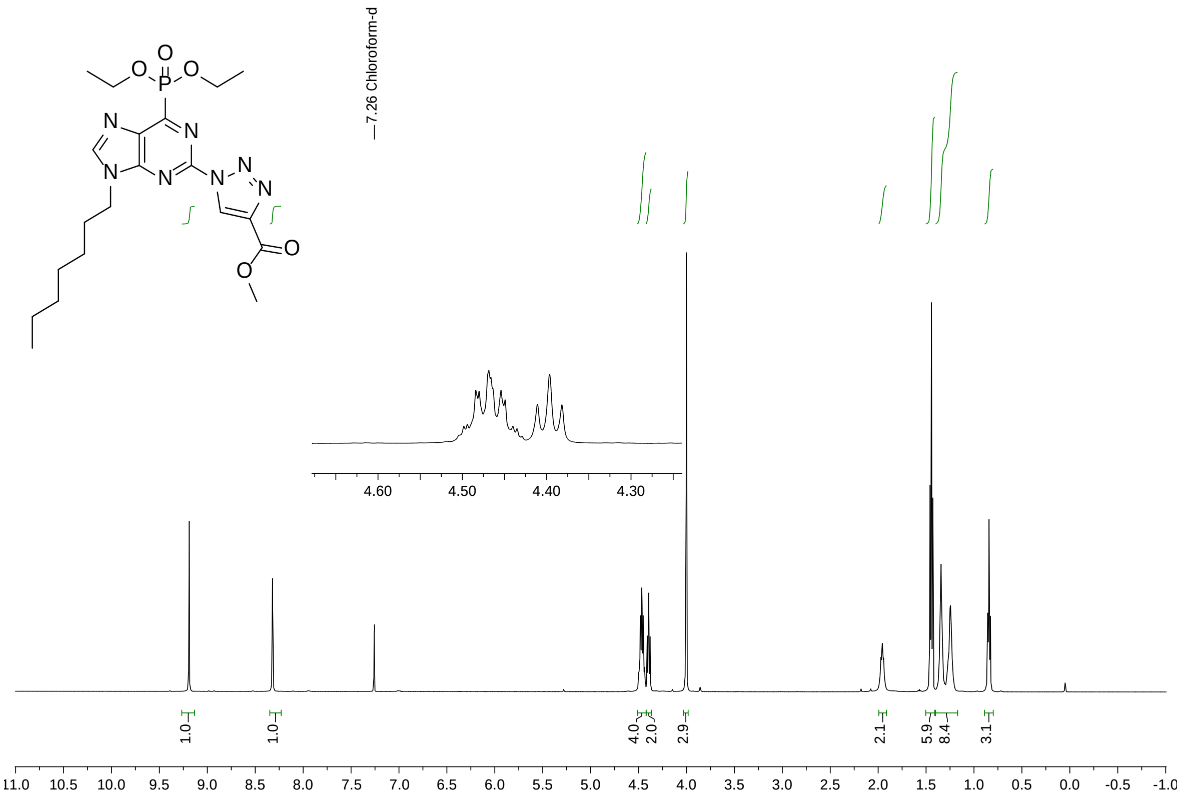

Figure S40: ^{1}H NMR (500 MHz, $CDCl_3$) spectrum.

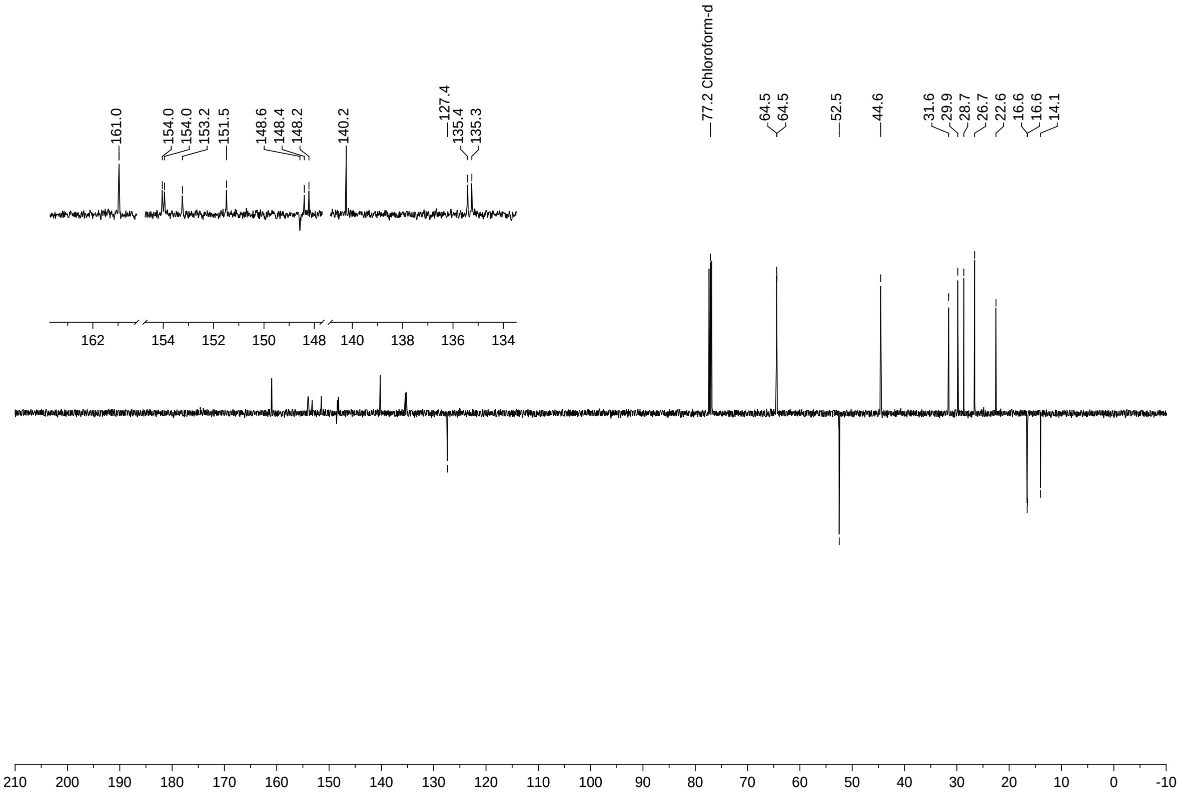

Figure S41: ^{13}C NMR (125.7 MHz, $CDCl_3$) spectrum.

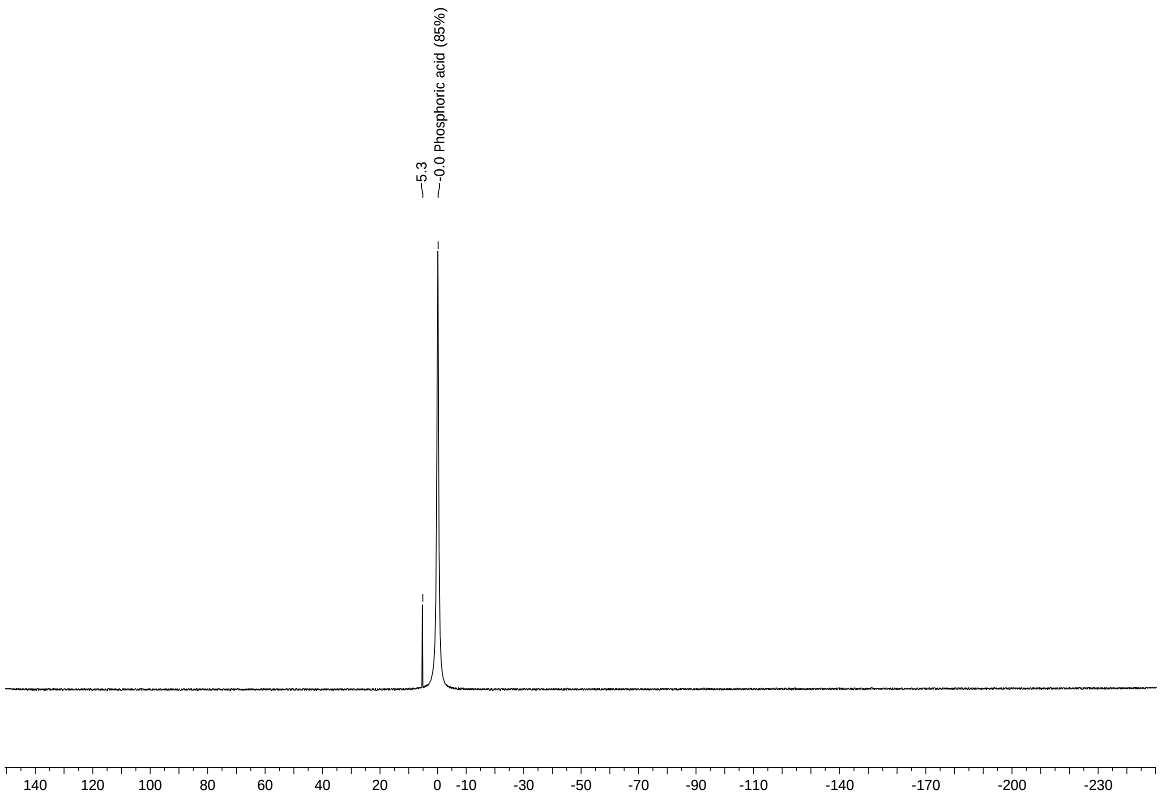

Figure S42: ^{31}P NMR (121 MHz, $CDCl_3$) spectrum.

Diethyl (2-(4-(3-cyanopropyl)-1*H*-1,2,3-triazol-1-yl)-9-heptyl-9*H*-purin-6-yl)phosphonate (4b)

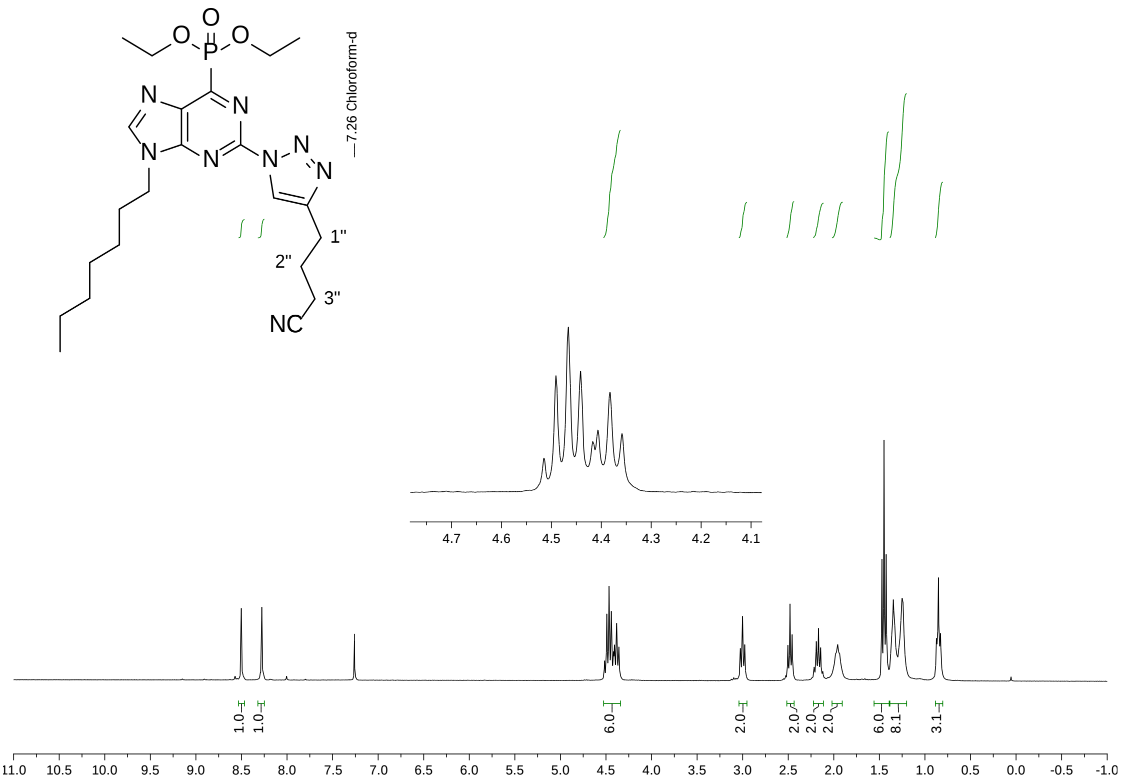

Figure S43: ^{1}H NMR (300 MHz, $CDCl_3$) spectrum.

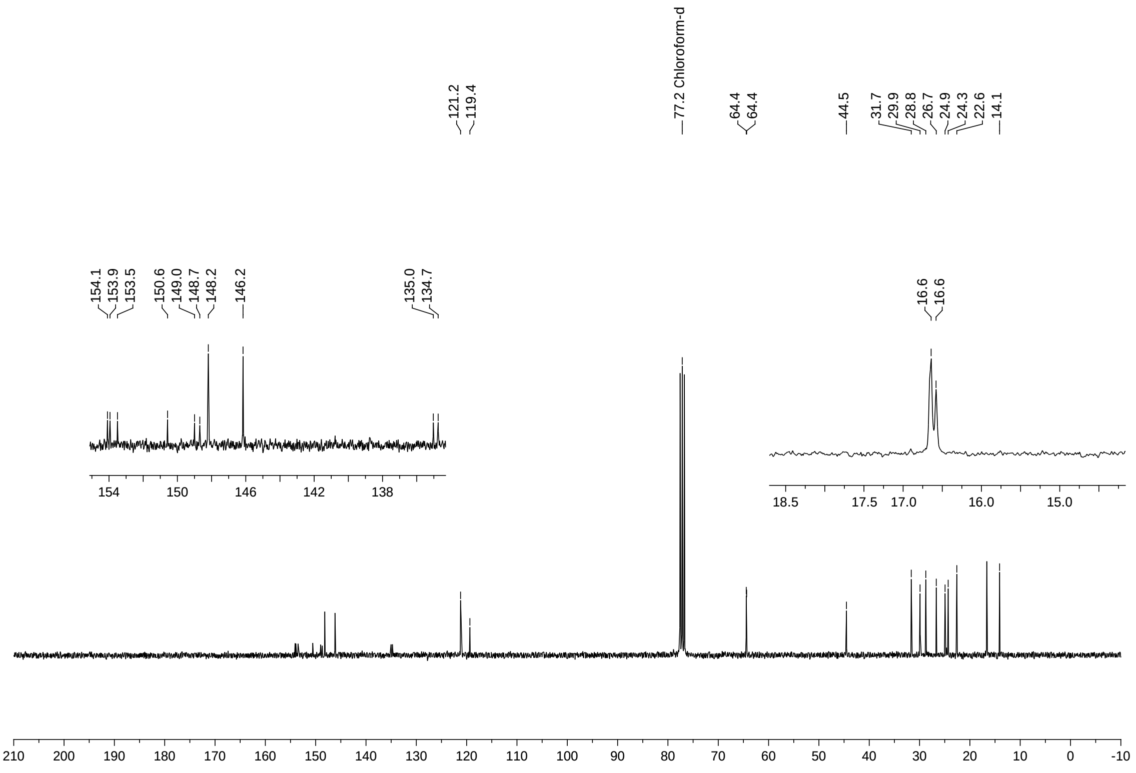

Figure S44: ^{13}C NMR (75.5 MHz, $CDCl_3$) spectrum.

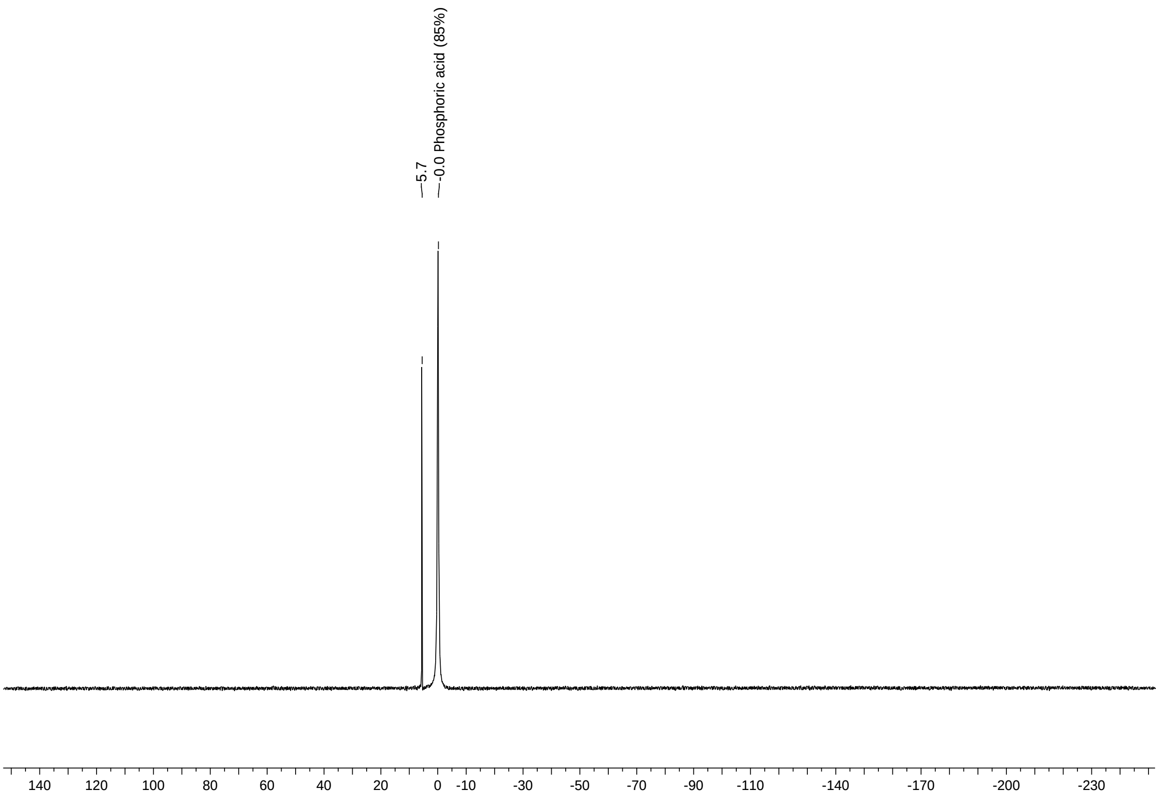

Figure S45: ^{31}P NMR (121 MHz, $CDCl_3$) spectrum.

Diethyl (2-(4-butyl-1*H*-1,2,3-triazol-1-yl)-9-heptyl-9*H*-purin-6-yl)phosphonate (4c)

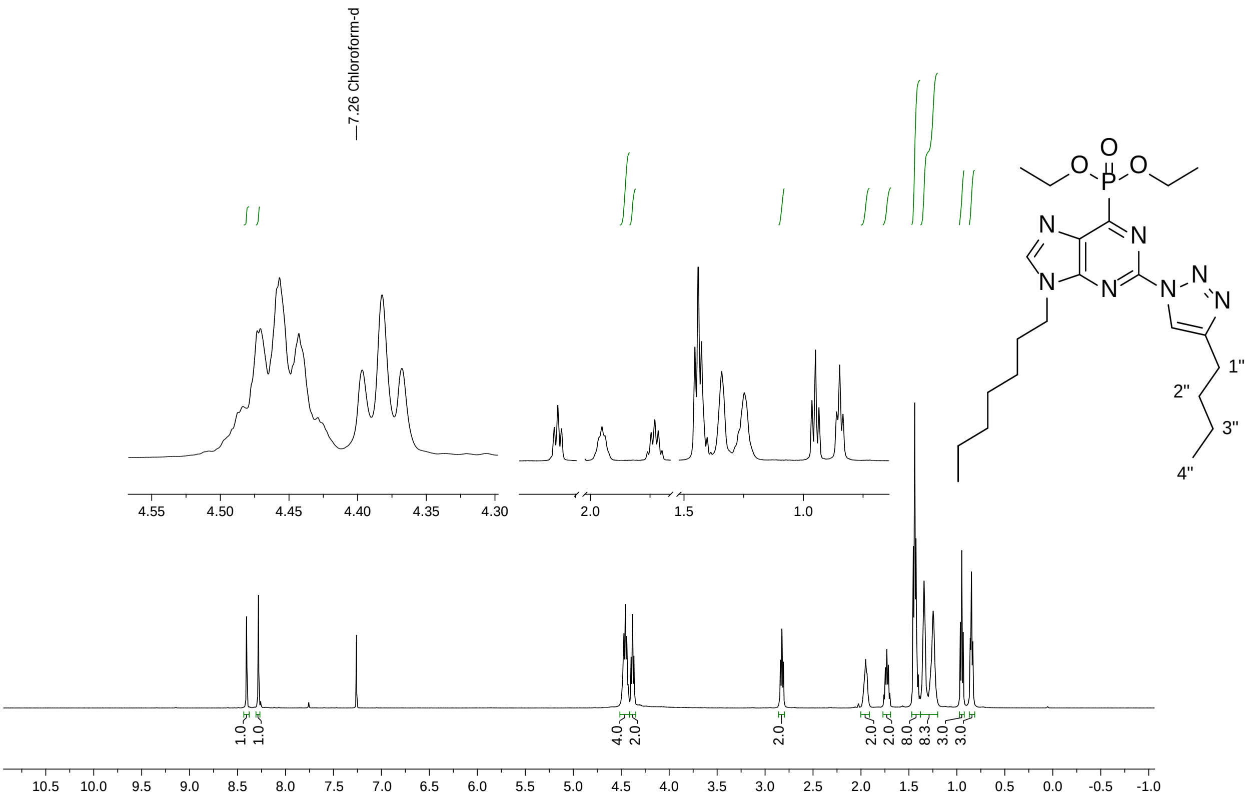

Figure S46: ^{1}H NMR (500 MHz, $CDCl_3$) spectrum.

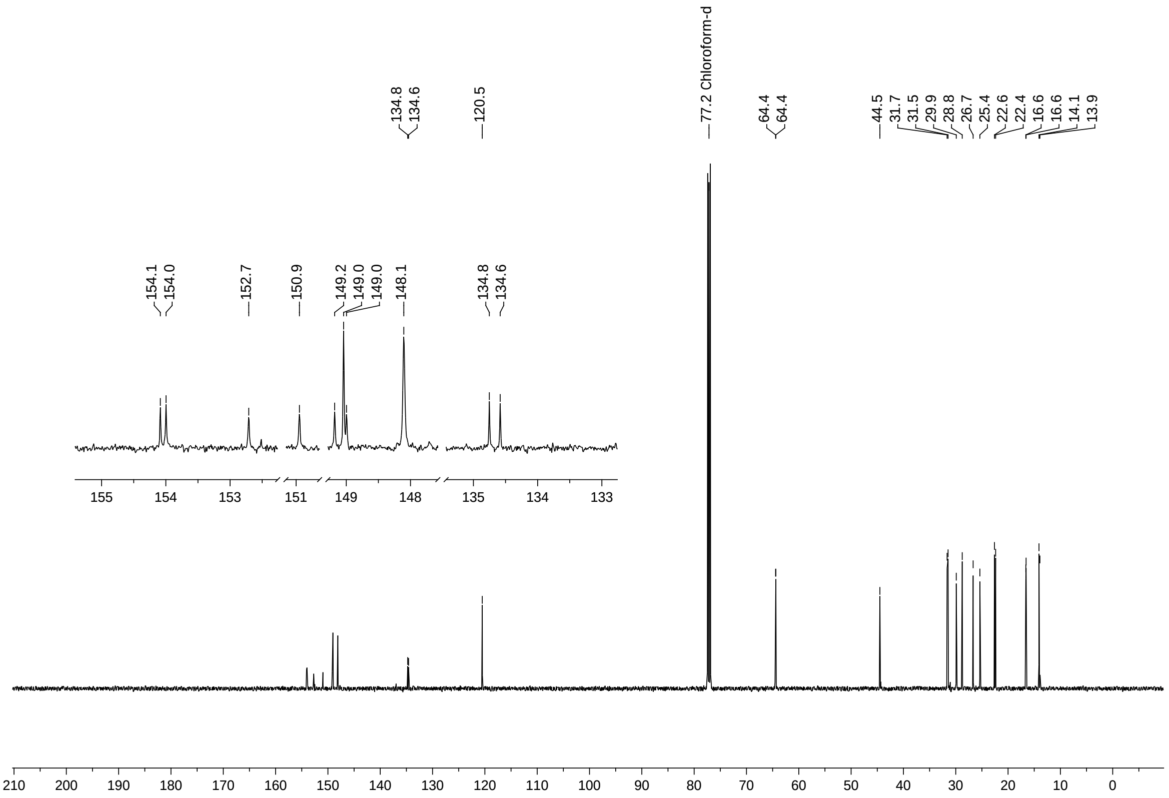

Figure S47: ^{13}C NMR (125.7 MHz, $CDCl_3$) spectrum.

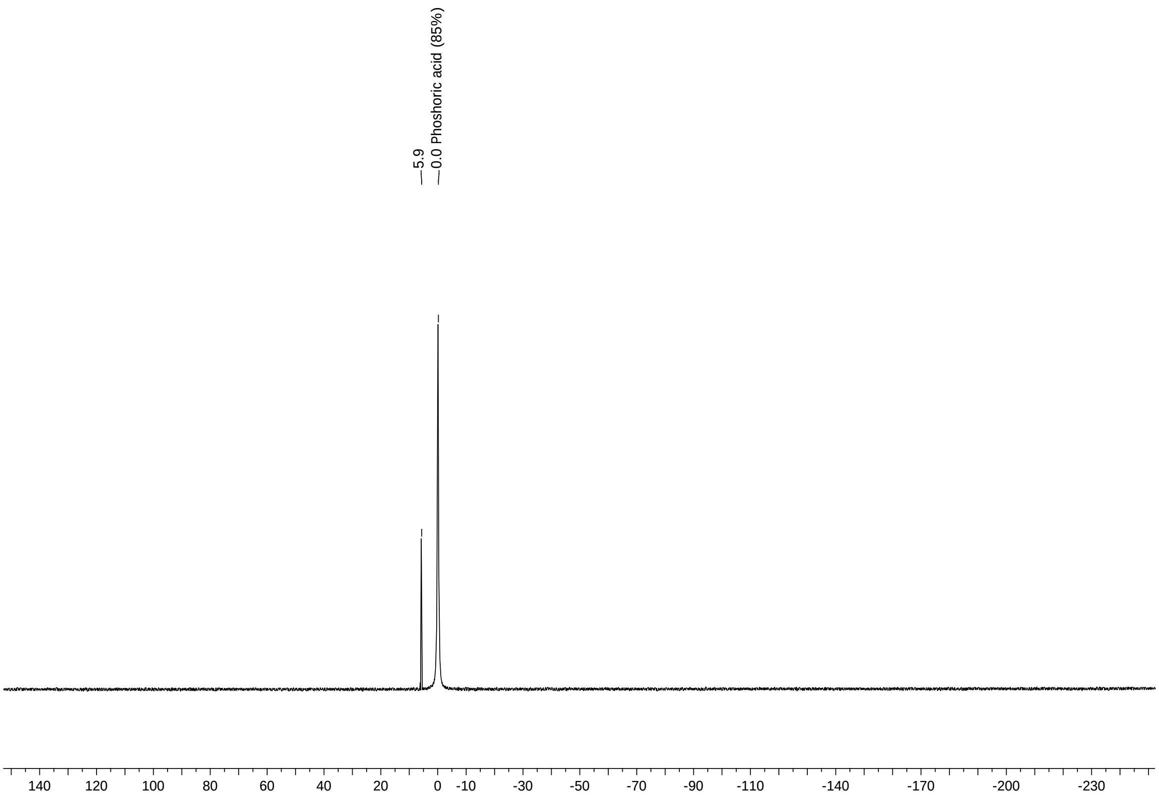

Figure S48: ^{31}P NMR (202 MHz, $CDCl_3$) spectrum.

Diethyl (2-(4-phenyl)-1*H*-1,2,3-triazol-1-yl)-9-heptyl-9*H*-purin-6-yl)phosphonate (4d)

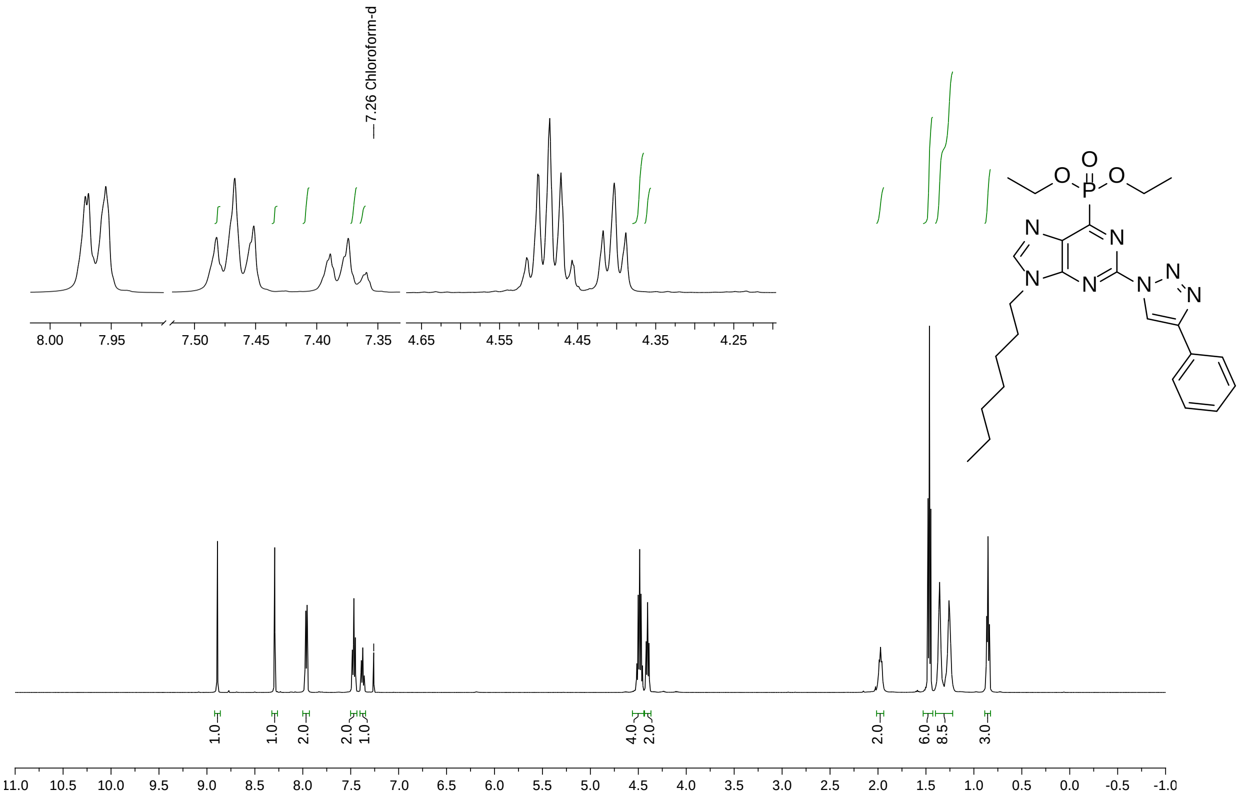

Figure S49: ^{1}H NMR (500 MHz, $CDCl_3$) spectrum.

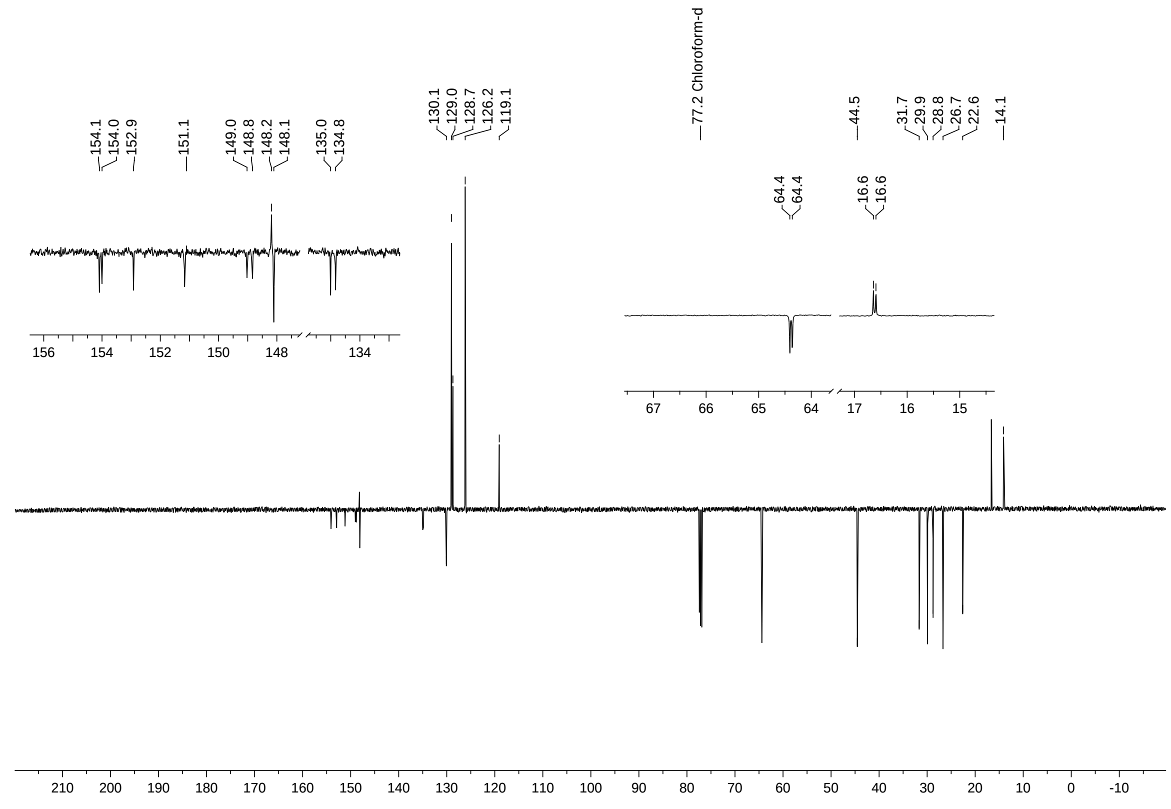

Figure S50: ^{13}C NMR (125.7 MHz, $CDCl_3$) spectrum.

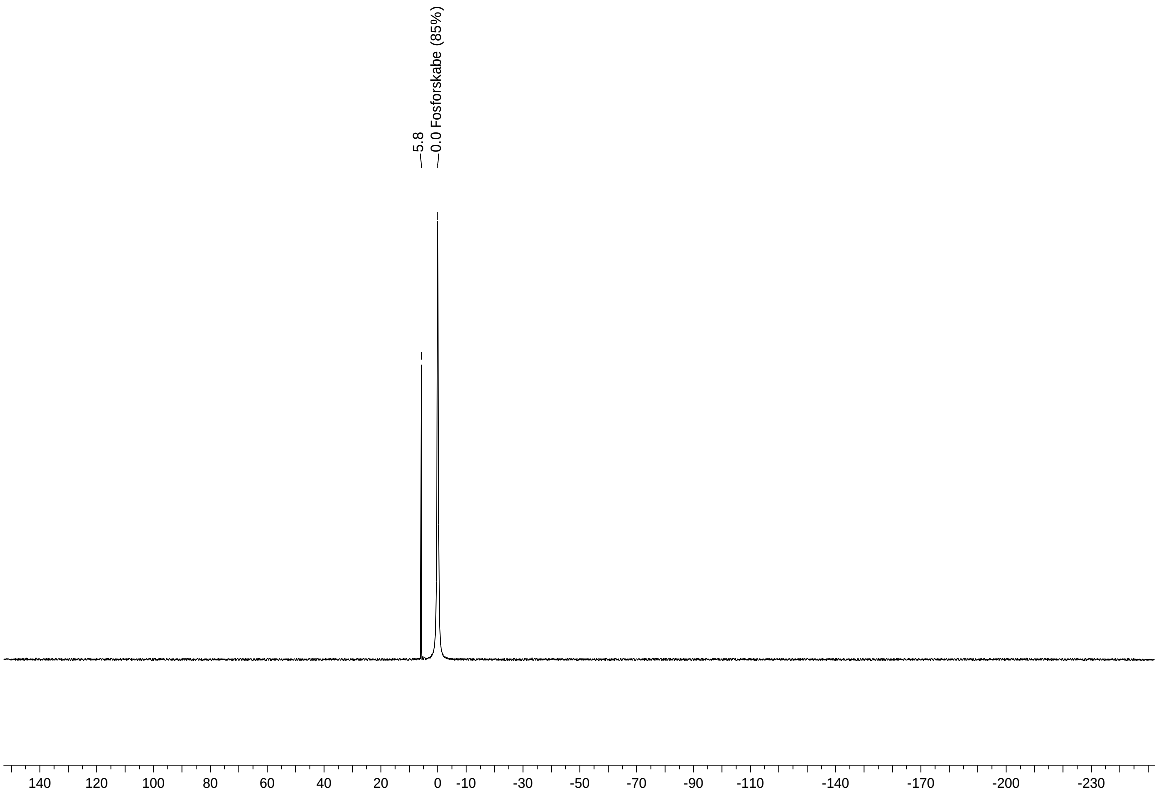

Figure S51: ^{31}P NMR (121 MHz, $CDCl_3$) spectrum.

Diethyl (2-(4-(4-cyanophenyl)-1*H*-1,2,3-triazol-1-yl)-9-heptyl-9*H*-purin-6-yl)phosphonate (4e)

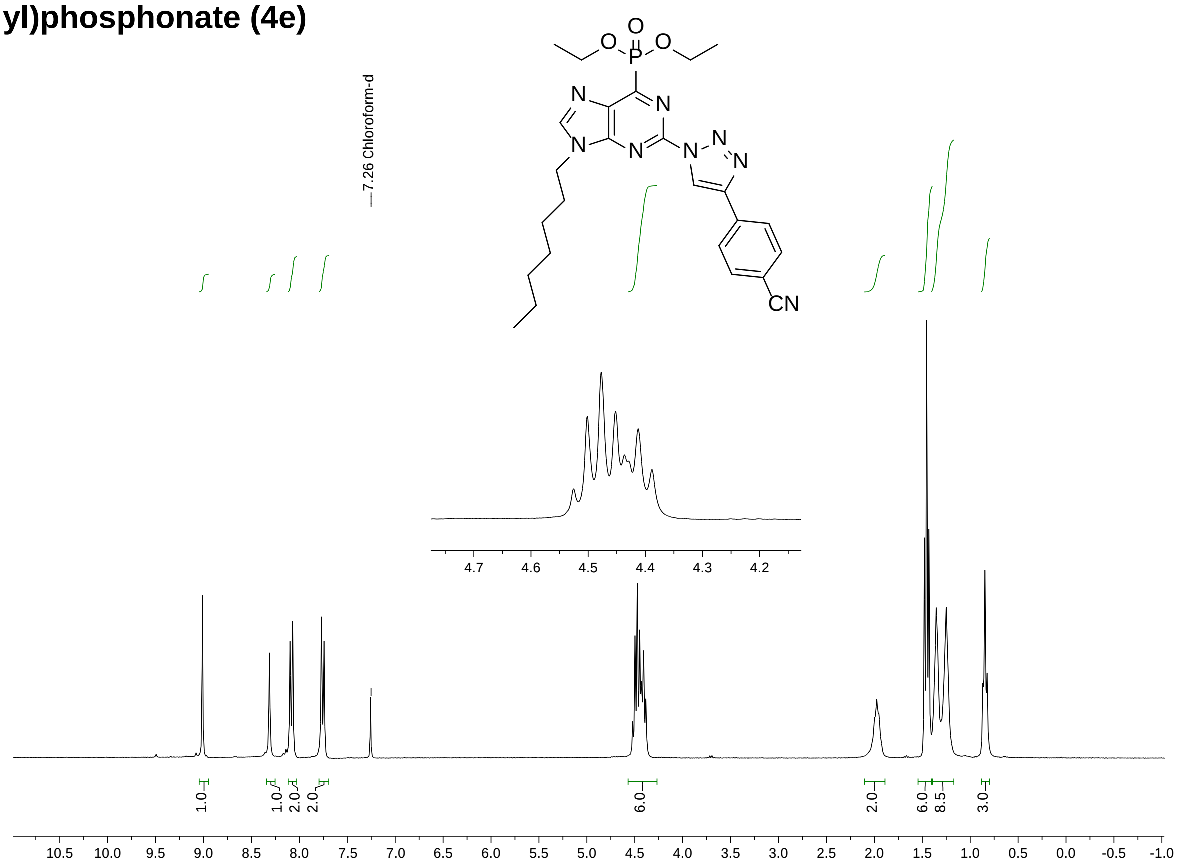

Figure S52: ^{1}H NMR (500 MHz, $CDCl_3$) spectrum.

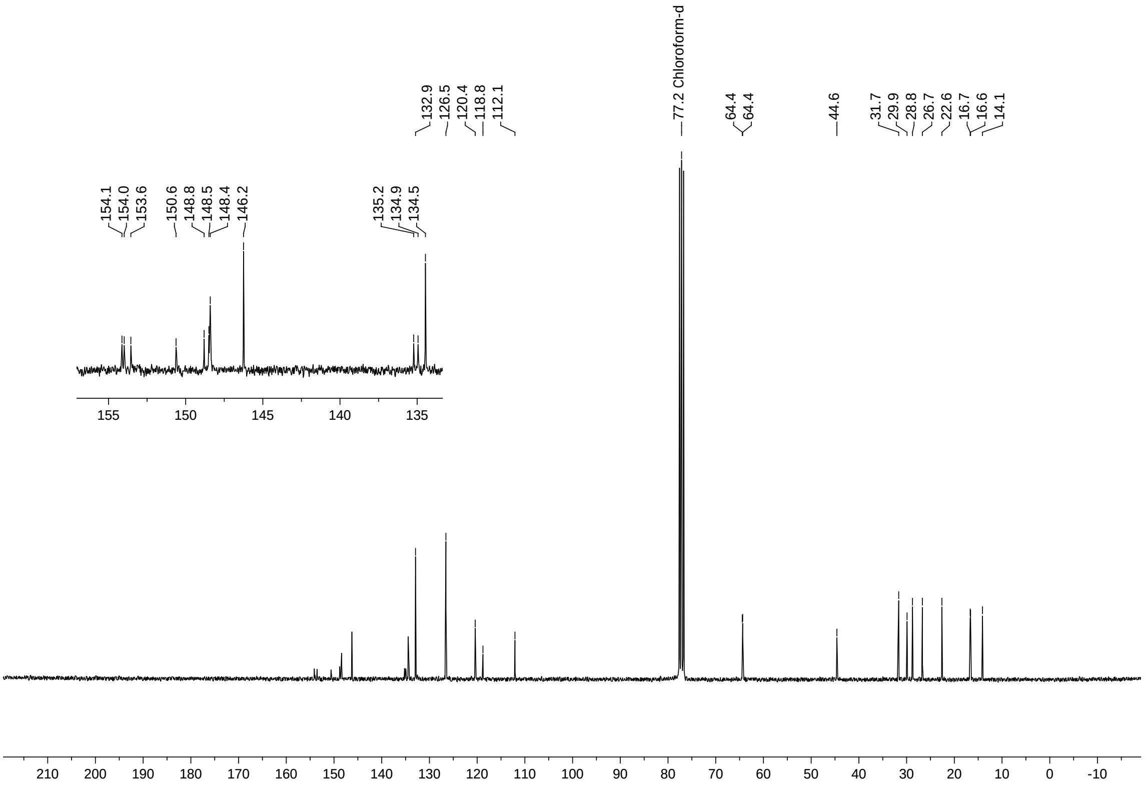

Figure S53: ^{13}C NMR (125.7 MHz, $CDCl_3$) spectrum

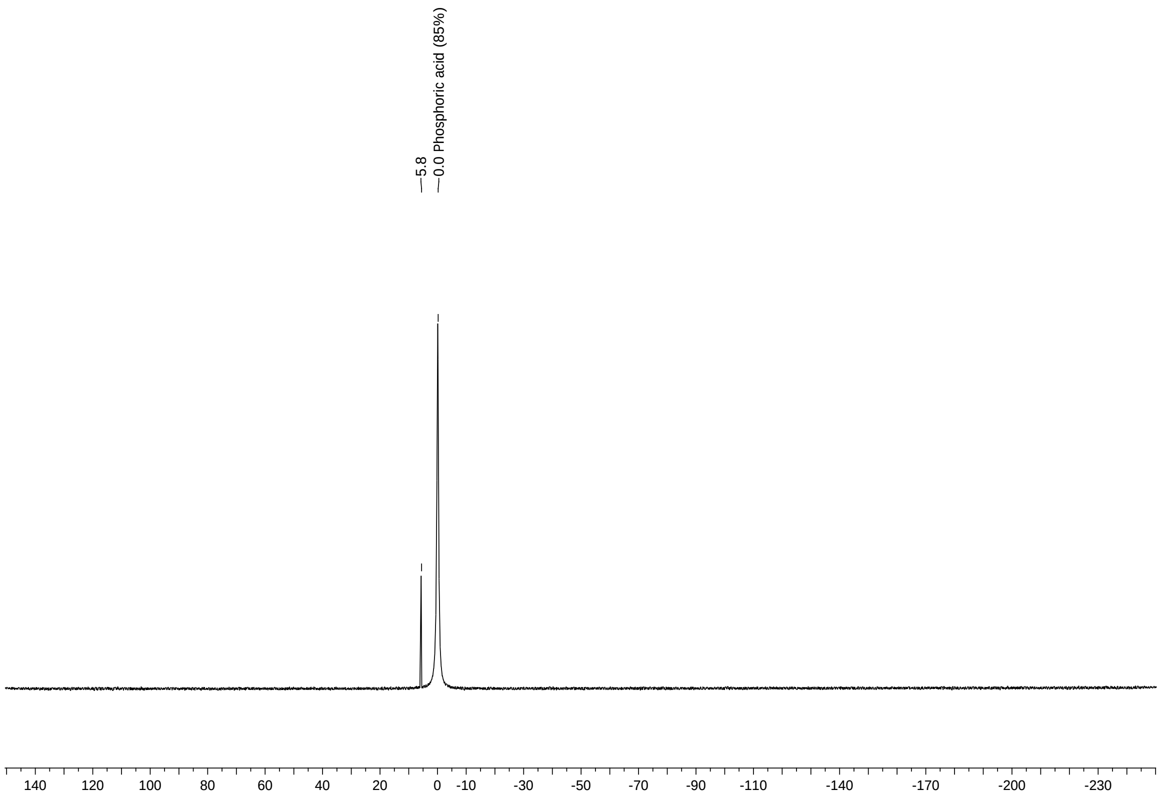

Figure S54: ^{31}P NMR (121 MHz, $CDCl_3$) spectrum.

Diethyl (9-heptyl-2-(4-(4-methoxyphenyl)-1*H*-1,2,3-triazol-1-yl)-9*H*-purin-6-yl)phosphonate (4f)

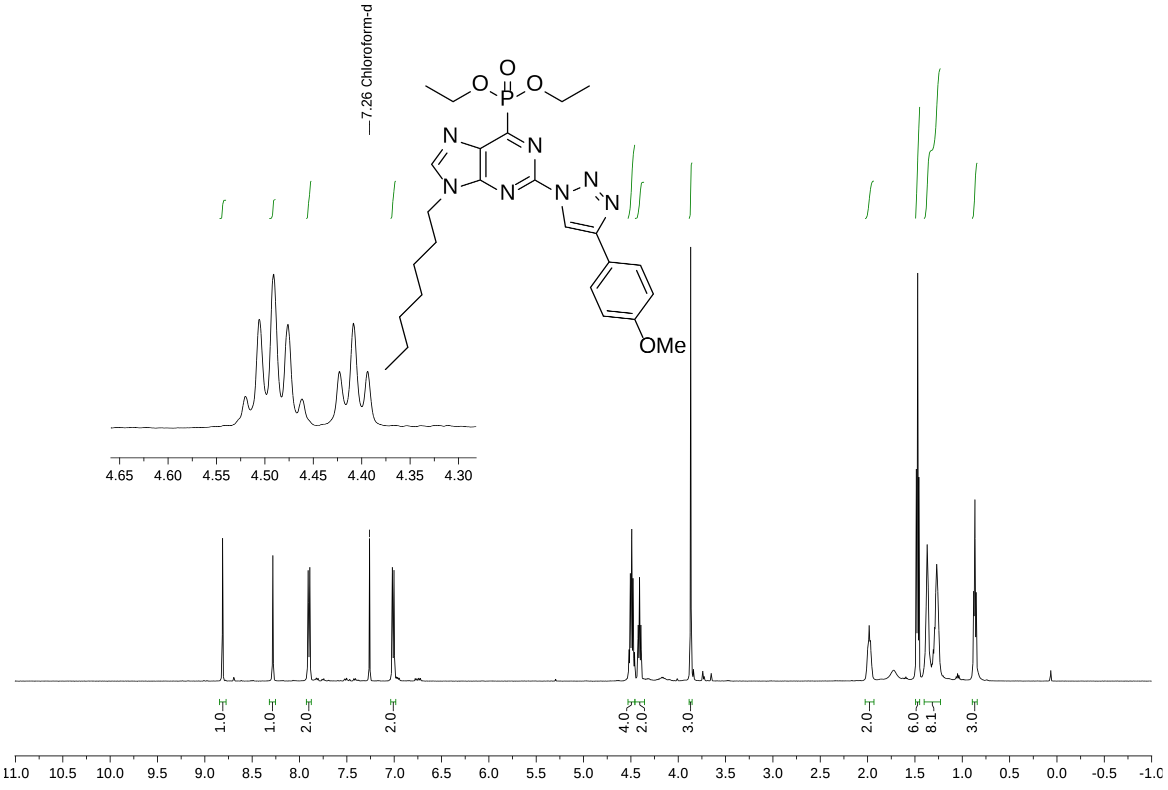

Figure S55: ^{1}H NMR (500 MHz, $CDCl_3$) spectrum

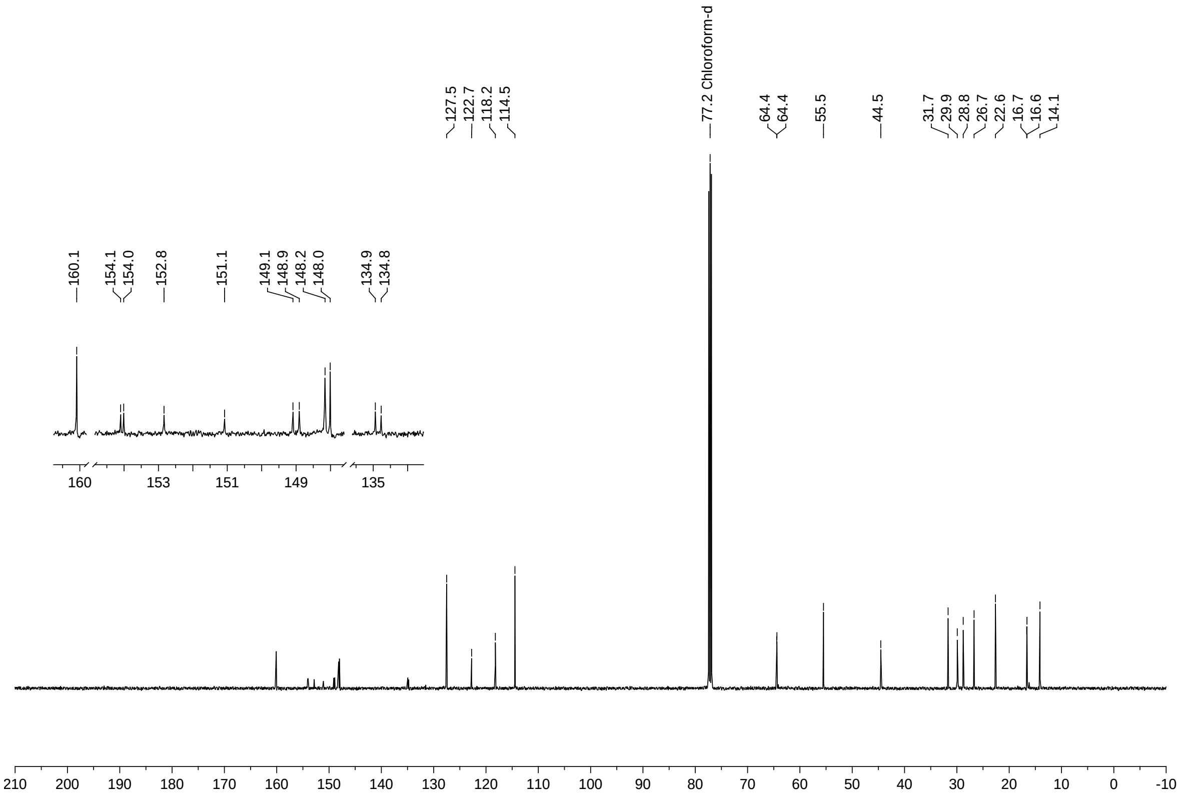

Figure S56: ^{13}C NMR (125.7 MHz, $CDCl_3$) spectrum

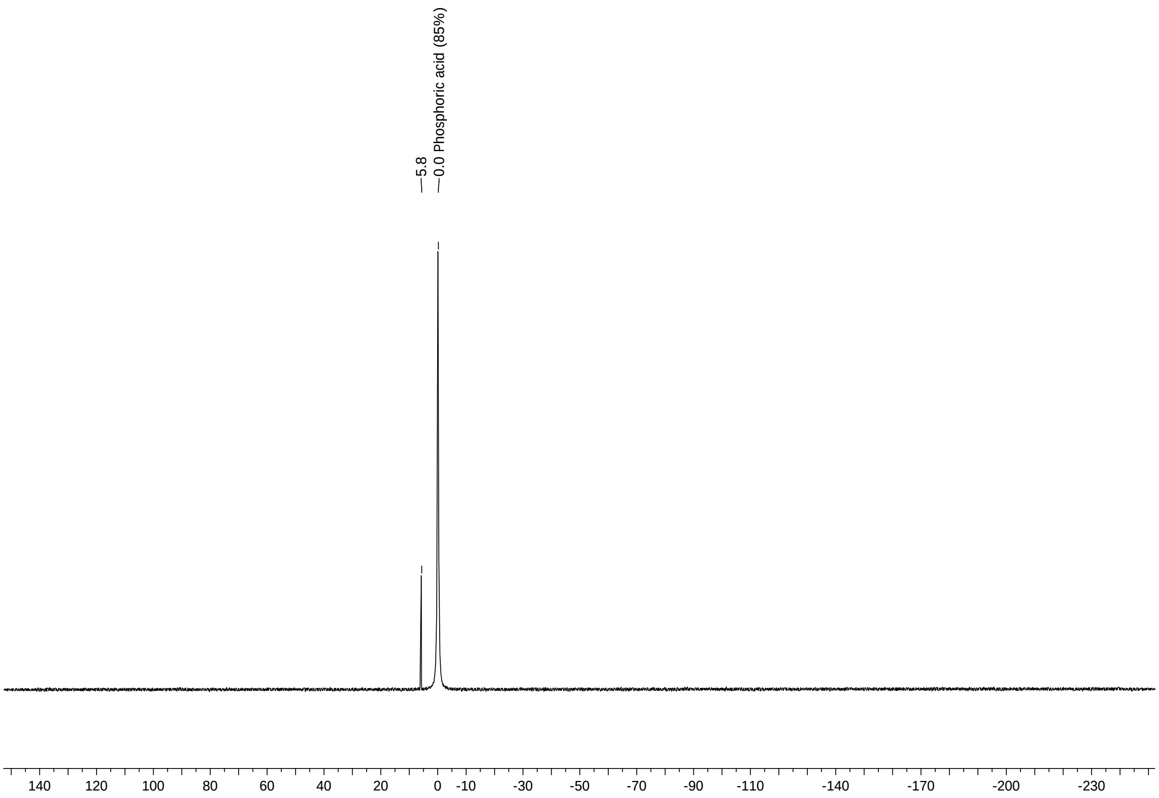

Figure S57: ^{31}P NMR (202 MHz, $CDCl_3$) spectrum.

Diethyl (2-(4-(4-chlorophenyl)-1*H*-1,2,3-triazol-1-yl)-9-heptyl-9*H*-purin-6-yl)phosphonate (4g)

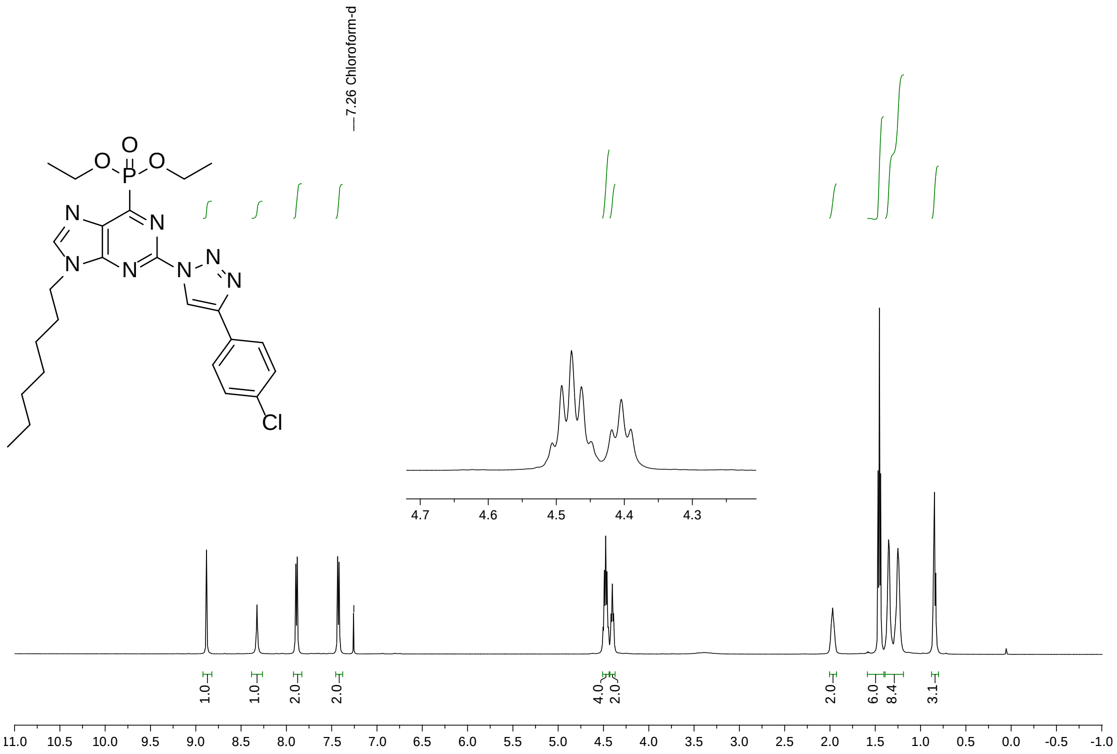

Figure S58: ^{1}H NMR (500 MHz, $CDCl_3$) spectrum

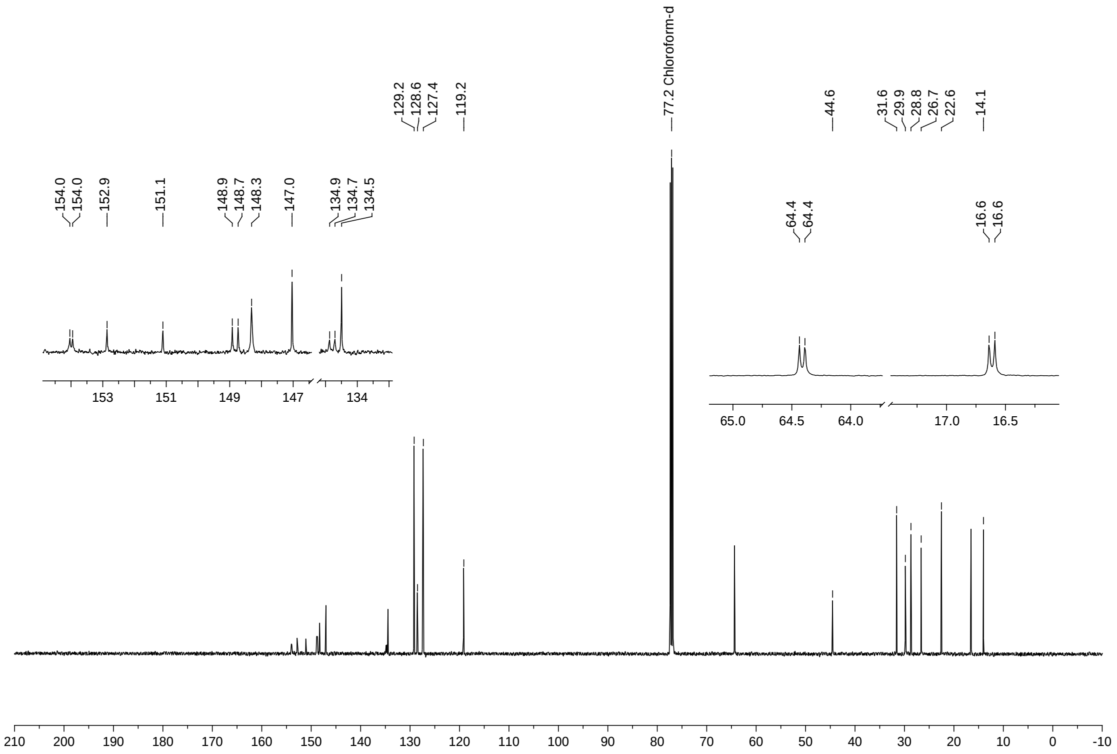

Figure S59: ^{13}C NMR (125.7 MHz, $CDCl_3$) spectrum

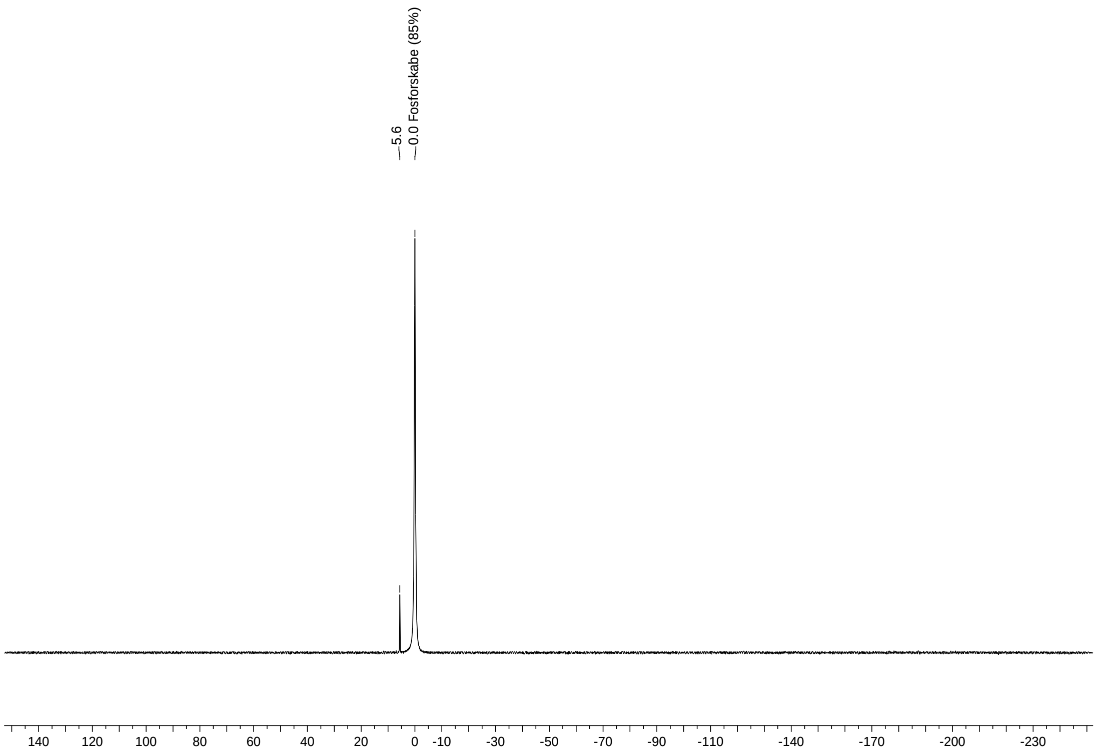

Figure S60: ^{31}P NMR (202 MHz, $CDCl_3$) spectrum.

Diethyl (2-(4-(2-fluorophenyl-1*H*-1,2,3-triazol-1-yl)-9-heptyl-9*H*-purin-6-yl)phosphonate (4h)

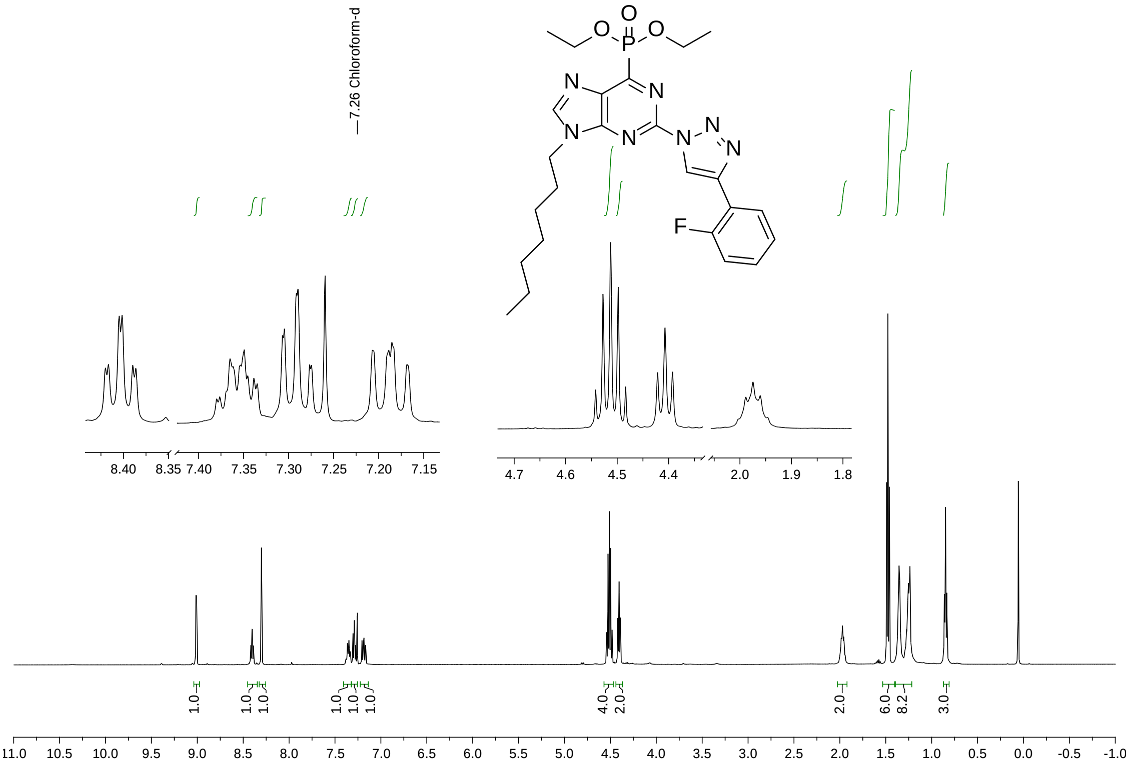

Figure S61: ^{1}H NMR (500 MHz, $CDCl_3$) spectrum

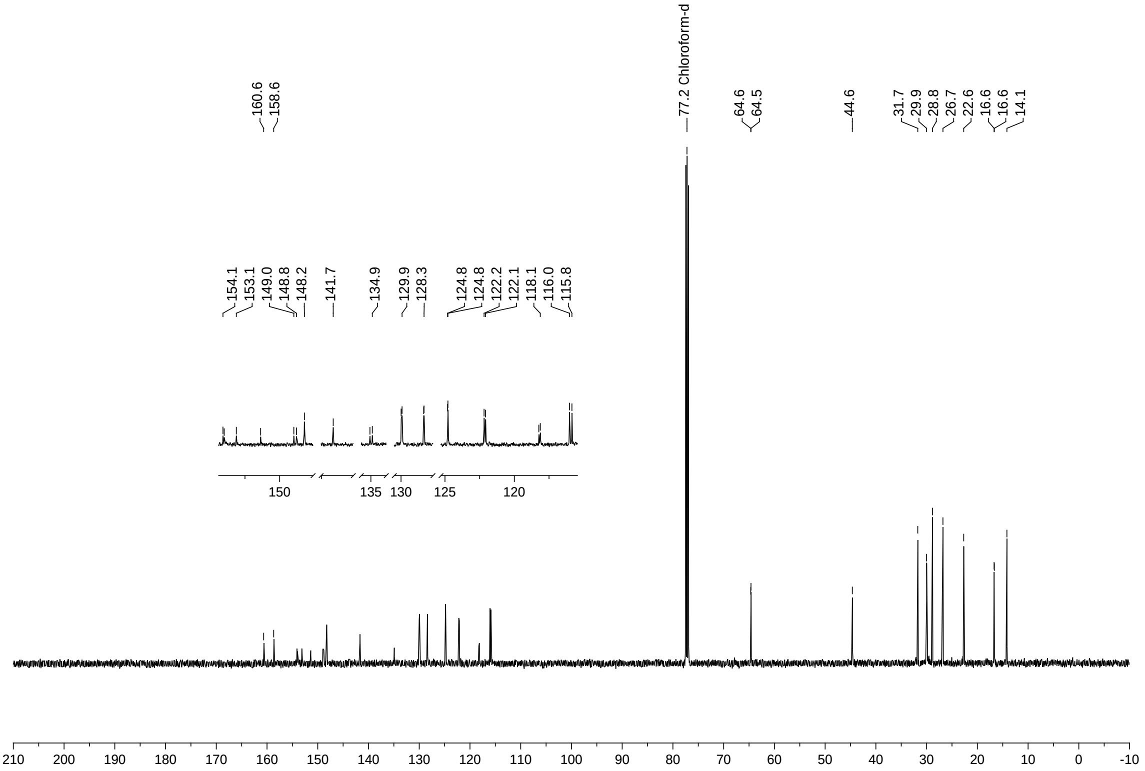

Figure S62: ^{13}C NMR (125.7 MHz, $CDCl_3$) spectrum

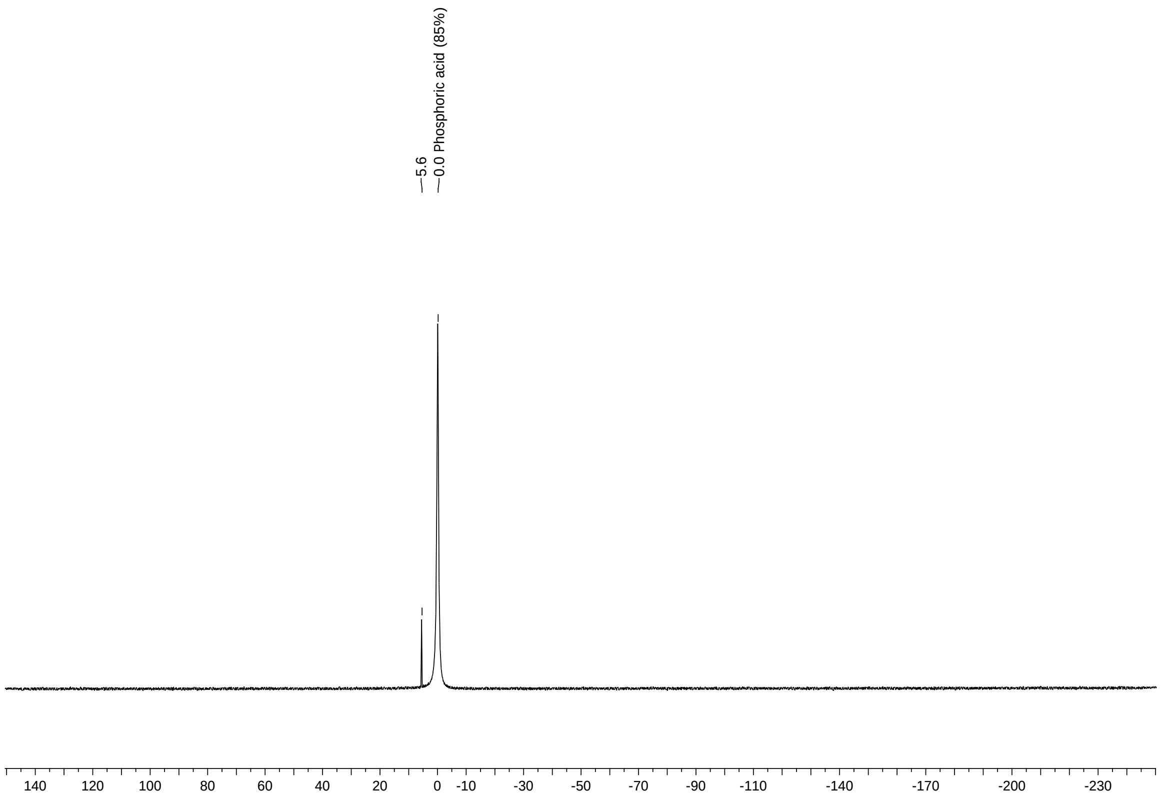

Figure S63: ^{31}P NMR (202 MHz, $CDCl_3$) spectrum.

Diethyl (9-heptyl-2-(4-(pyrdin-2-yl)-1*H*-1,2,3-triazol-1-yl)-9*H*-purin-6-yl)phosphonate (4i)

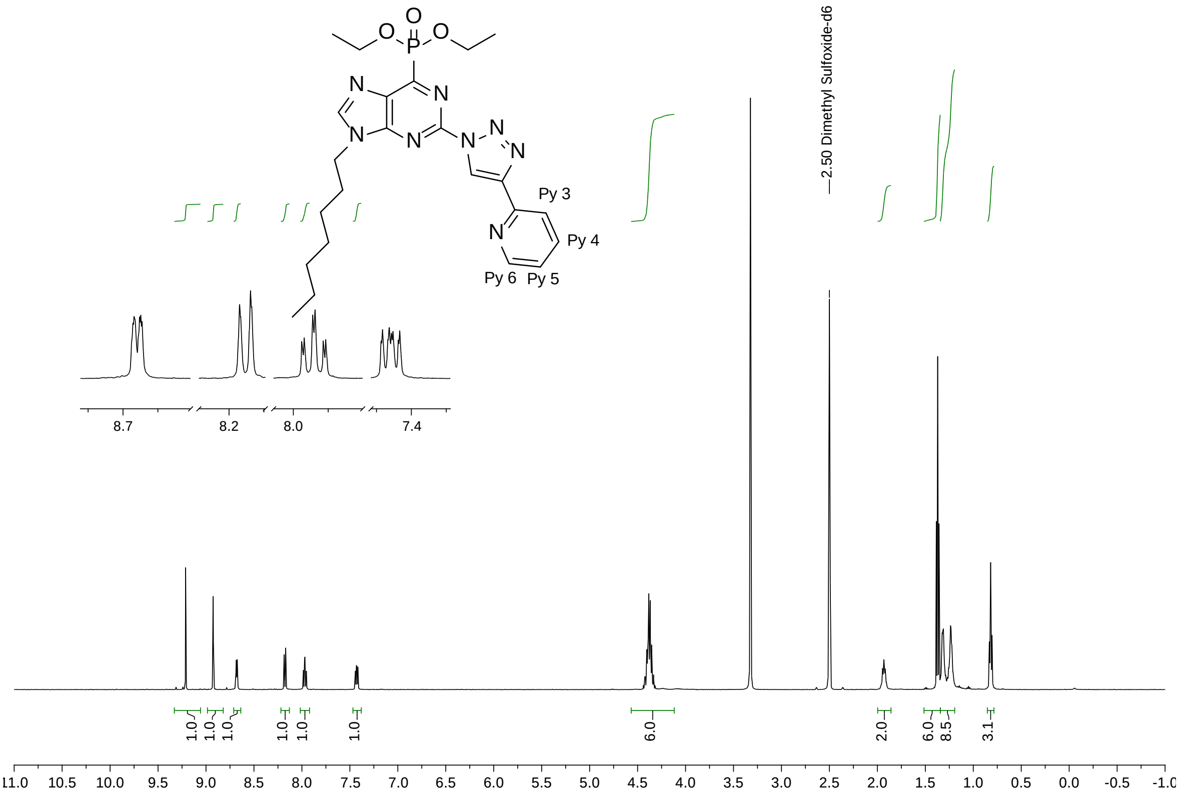

Figure S64: ^{1}H NMR (500 MHz, DMSO-d_6) spectrum

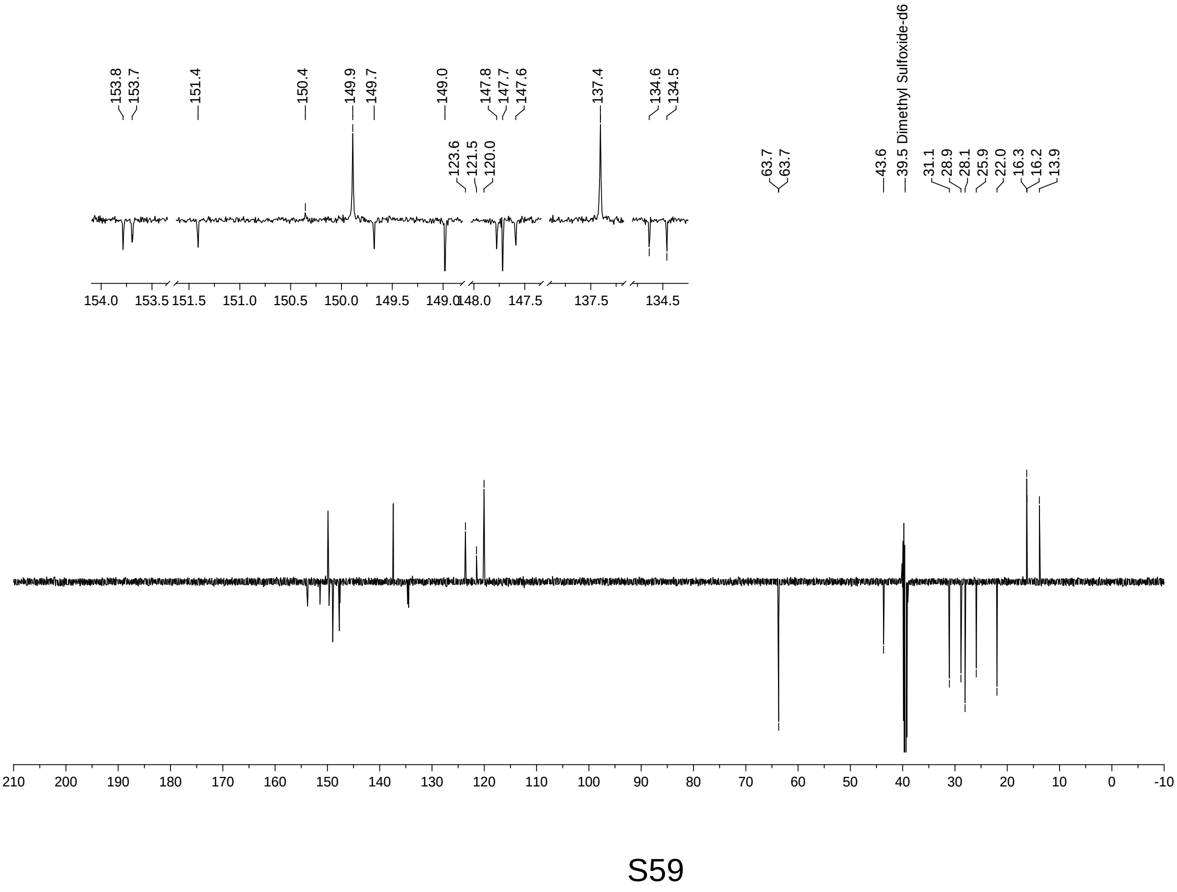

Figure S65: ^{13}C NMR (125.7 MHz, DMSO-d_6) spectrum

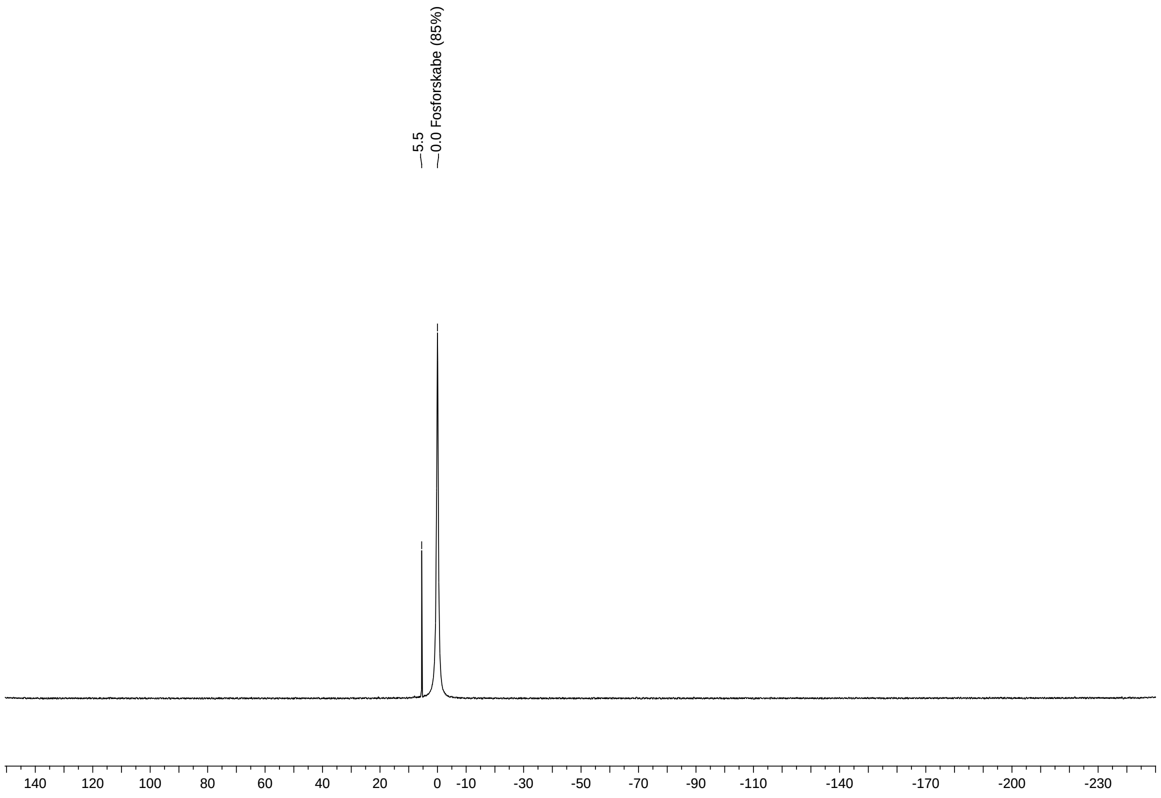

Figure S66: ^{31}P NMR (202 MHz, DMSO-d_6) spectrum.

Check CIF for compound 4d

checkCIF/PLATON report

Structure factors have been supplied for datablock(s) kek_70b

THIS REPORT IS FOR GUIDANCE ONLY. IF USED AS PART OF A REVIEW PROCEDURE FOR PUBLICATION, IT SHOULD NOT REPLACE THE EXPERTISE OF AN EXPERIENCED CRYSTALLOGRAPHIC REFEREE.

No syntax errors found. CIF dictionary Interpreting this report

Datablock: kek_70b

```
Bond precision:   C-C = 0.0063 A          Wavelength=1.54184

Cell:             a=15.7906(2)     b=15.1059(2)        c=21.9456(3)
                  alpha=90         beta=106.461(1)     gamma=90
Temperature:      160 K

                  Calculated                  Reported
Volume            5020.16(12)                 5020.15(12)
Space group       P 21/n                      P 21/n
Hall group        -P 2yn                      -P 2yn
Moiety formula    C24 H32 N7 O3 P             C24 H32 N7 O3 P
Sum formula       C24 H32 N7 O3 P             C24 H32 N7 O3 P
Mr                497.54                      497.54
Dx,g cm-3         1.317                       1.317
Z                 8                           8
Mu (mm-1)         1.304                       1.304
F000              2112.0                      2112.0
F000'             2120.28
h,k,lmax          19,19,27                    19,19,27
Nref              10631                       10318
Tmin,Tmax         0.925,0.949                 0.867,1.000
Tmin'             0.770

Correction method= # Reported T Limits: Tmin=0.867 Tmax=1.000
AbsCorr = MULTI-SCAN

Data completeness= 0.971          Theta(max)= 77.157

R(reflections)= 0.0801( 9670)     wR2(reflections)= 0.2723( 10318)

S = 1.177                  Npar= 638
```

```
The following ALERTS were generated. Each ALERT has the format
       test-name_ALERT_alert-type_alert-level.
Click on the hyperlinks for more details of the test.
```

Alert level B

```
PLAT930_ALERT_2_B FCF-based   Twin Law ( 0 0 1)[ 2 0 5] Est.d BASF       0.22 Check
```

Author Response: TWIN instruction with recommended twin Law (0 0 1)[2 0 5] (Est.d BASF = 0.22) was included in the refinement process but gave a limited improvement of the structure. The refined value of BASF was 0.0378.

Alert level C

```
DIFMX02_ALERT_1_C  The maximum difference density is > 0.1*ZMAX*0.75
            The relevant atom site should be identified.
PLAT084_ALERT_3_C High wR2 Value (i.e. > 0.25) ..................       0.27 Report
PLAT094_ALERT_2_C Ratio of Maximum / Minimum Residual Density ....       2.79 Report
PLAT097_ALERT_2_C Large Reported Max.  (Positive) Residual Density       1.32 eA-3
PLAT340_ALERT_3_C Low Bond Precision on  C-C Bonds ..............    0.00633 Ang.
PLAT911_ALERT_3_C Missing FCF Refl Between Thmin & STh/L=    0.600         17 Report
PLAT918_ALERT_3_C Reflection(s) with I(obs) much Smaller I(calc) .          5 Check
PLAT939_ALERT_3_C Large Value of Not (SHELXL) Weight Optimized S .      10.17 Check
```

Alert level G

```
PLAT083_ALERT_2_G SHELXL Second Parameter in WGHT  Unusually Large      13.54 Why ?
PLAT432_ALERT_2_G Short Inter X...Y Contact  O28       ..C41             3.00 Ang.
                                                      x,y,z  =      1_555 Check
PLAT870_ALERT_4_G ALERTS Related to Twinning Effects Suppressed ..          ! Info
PLAT883_ALERT_1_G No Info/Value for _atom_sites_solution_primary .     Please Do !
PLAT910_ALERT_3_G Missing # of FCF Reflection(s) Below Theta(Min).          1 Note
PLAT912_ALERT_4_G Missing # of FCF Reflections Above STh/L=  0.600        372 Note
PLAT913_ALERT_3_G Missing # of Very Strong Reflections in FCF ....          3 Note
PLAT931_ALERT_5_G CIFcalcFCF  Twin Law ( 0 0 1)        Est.d BASF       0.23 Check
PLAT941_ALERT_3_G Average HKL Measurement Multiplicity ..........        4.8 Low
PLAT965_ALERT_2_G The SHELXL WEIGHT Optimisation has not Converged     Please Check
```

```
  0 ALERT level A = Most likely a serious problem - resolve or explain
  1 ALERT level B = A potentially serious problem, consider carefully
  8 ALERT level C = Check. Ensure it is not caused by an omission or oversight
 10 ALERT level G = General information/check it is not something unexpected

  2 ALERT type 1 CIF construction/syntax error, inconsistent or missing data
  6 ALERT type 2 Indicator that the structure model may be wrong or deficient
  8 ALERT type 3 Indicator that the structure quality may be low
  2 ALERT type 4 Improvement, methodology, query or suggestion
  1 ALERT type 5 Informative message, check
```

It is advisable to attempt to resolve as many as possible of the alerts in all categories. Often the minor alerts point to easily fixed oversights, errors and omissions in your CIF or refinement strategy, so attention to these fine details can be worthwhile. In order to resolve some of the more serious problems it may be necessary to carry out additional measurements or structure refinements. However, the purpose of your study may justify the reported deviations and the more serious of these should normally be commented upon in the discussion or experimental section of a paper or in the "special_details" fields of the CIF. checkCIF was carefully designed to identify outliers and unusual parameters, but every test has its limitations and alerts that are not important in a particular case may appear. Conversely, the absence of alerts does not guarantee there are no aspects of the results needing attention. It is up to the individual to critically assess their own results and, if necessary, seek expert advice.

Publication of your CIF in IUCr journals

A basic structural check has been run on your CIF. These basic checks will be run on all CIFs submitted for publication in IUCr journals (*Acta Crystallographica*, *Journal of Applied Crystallography*, *Journal of Synchrotron Radiation*); however, if you intend to submit to *Acta Crystallographica Section C* or *E* or *IUCrData*, you should make sure that full publication checks are run on the final version of your CIF prior to submission.

Publication of your CIF in other journals

Please refer to the *Notes for Authors* of the relevant journal for any special instructions relating to CIF submission.

PLATON version of 18/09/2020; check.def file version of 20/08/2020

Datablock kek_70b - ellipsoid plot

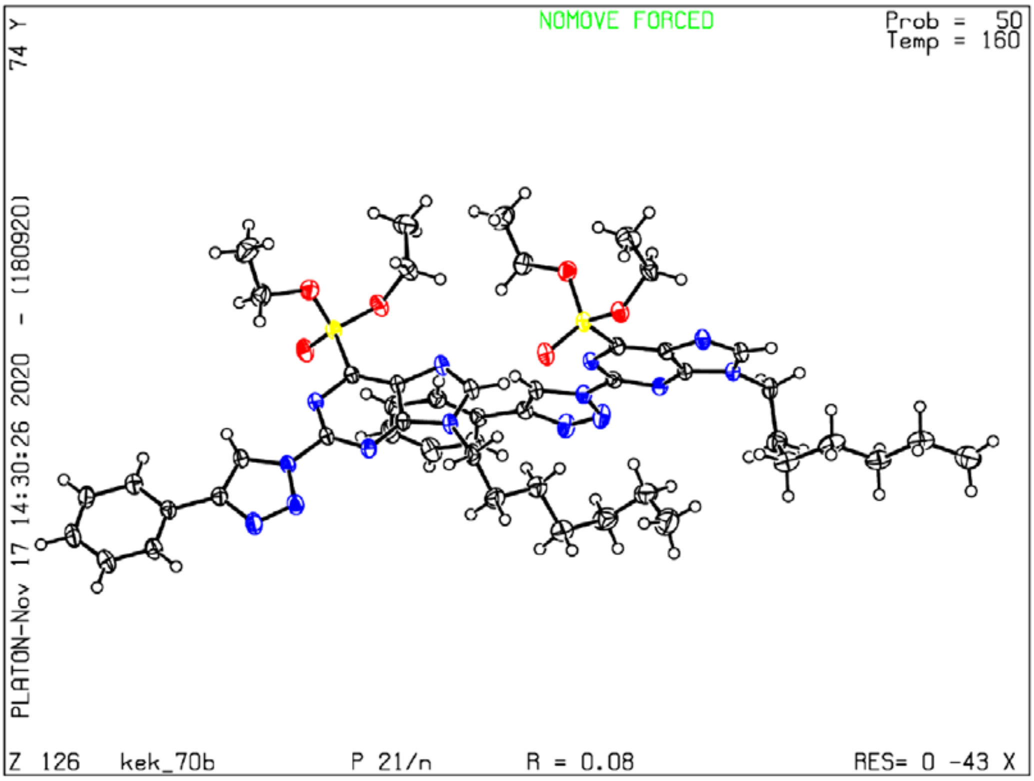

References

1. Šišuļins, A.; Bucevičius, J.; Tseng, Y.-T.; Novosjolova, I.; Traskovskis, K.; Bizdēna, Ē.; Chang, H.-T.; Tumkevičius, S.; Turks, M. *Beilstein J. Org. Chem.* **2019**, *15*, 474–489. doi:10.3762/bjoc.15.41
